# Supplementary material for: Inhibition of SUV39H1 reduces tumor angiogenesis via Notch1 in oral squamous cell carcinoma
Source: PeerJ. 2024 Apr 19;12:e17222. doi: 10.7717/peerj.17222 (PMC11034493; doi:10.7717/peerj.17222)
Supplement: Supplemental Information 1 — The expression of NOTCH1 was positively correlated with that of CD31. [file peerj-12-17222-s001.pdf]

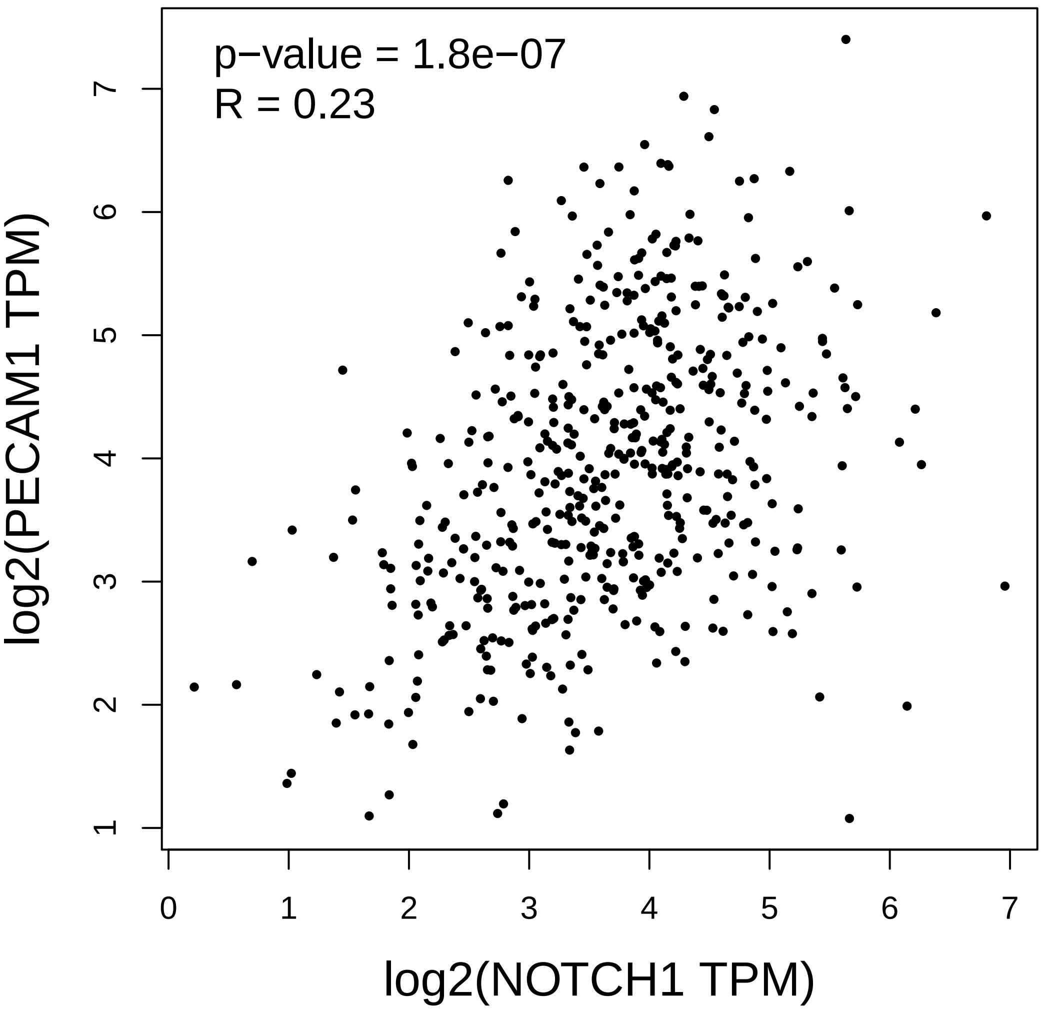

p−value = 1.8e−07
R = 0.23
log2(PECAM1 TPM)
log2(NOTCH1 TPM)
1
2
3
4
5
6
7
0
1
2
3
4
5
6
7
