# Supplementary material for: Inhibition of SUV39H1 reduces tumor angiogenesis via Notch1 in oral squamous cell carcinoma
Source: PeerJ. 2024 Apr 19;12:e17222. doi: 10.7717/peerj.17222 (PMC11034493; doi:10.7717/peerj.17222)
Supplement: Supplemental Information 4 [file peerj-12-17222-s004.pdf]

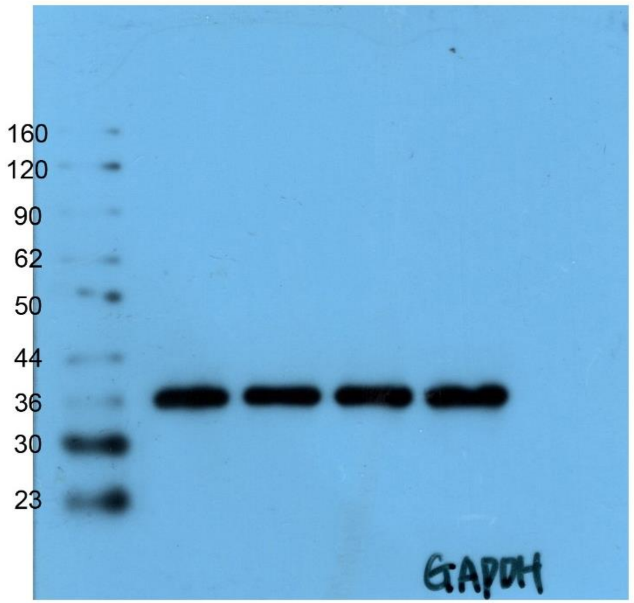
160
120
90
62
50
44
36
30
23
GAPDH

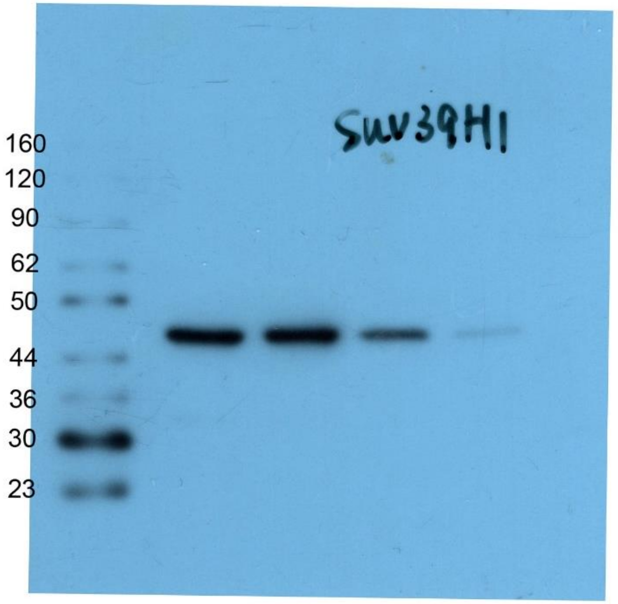
Suv39H1
160
120
90
62
50
44
36
30
23

Tube formation

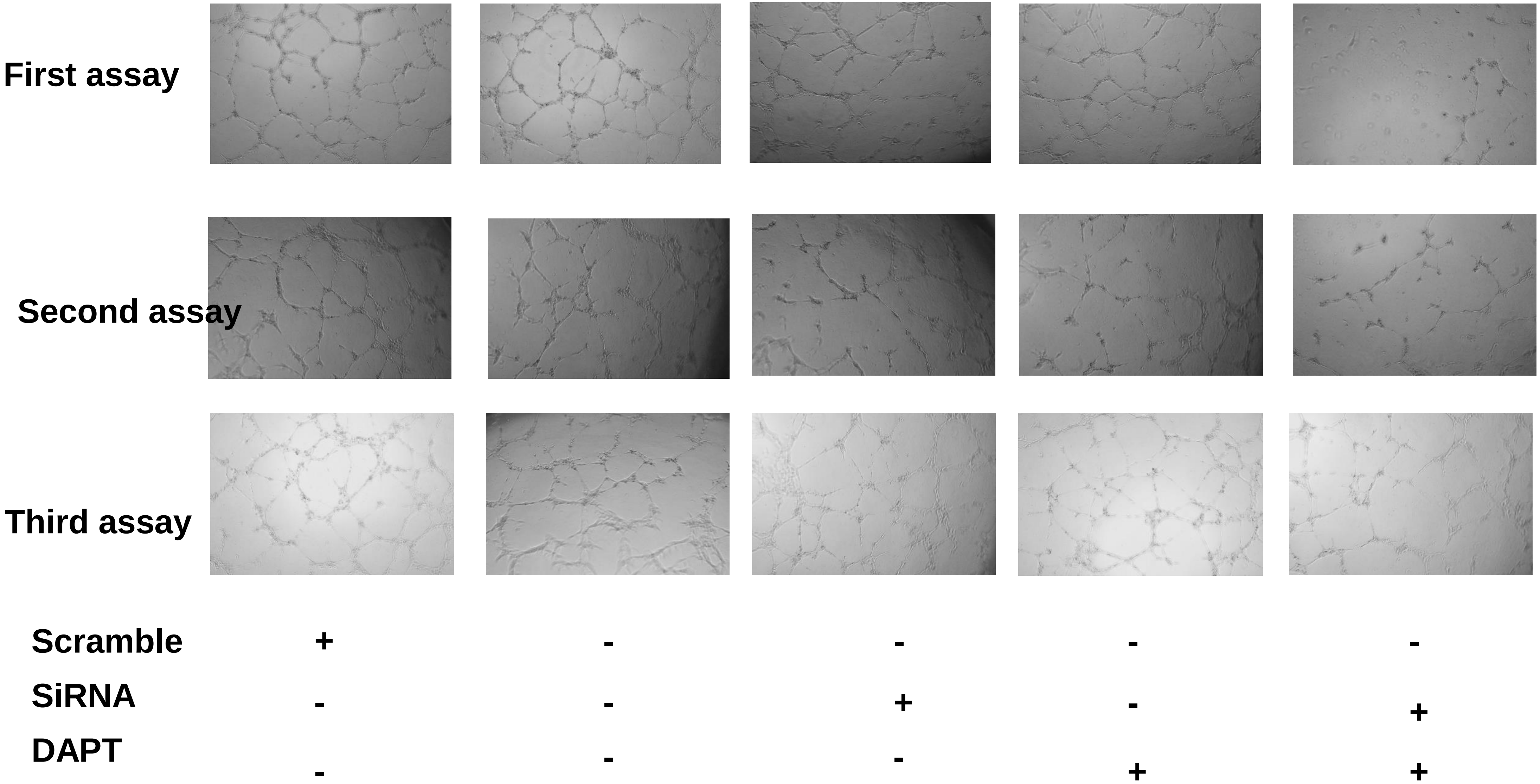

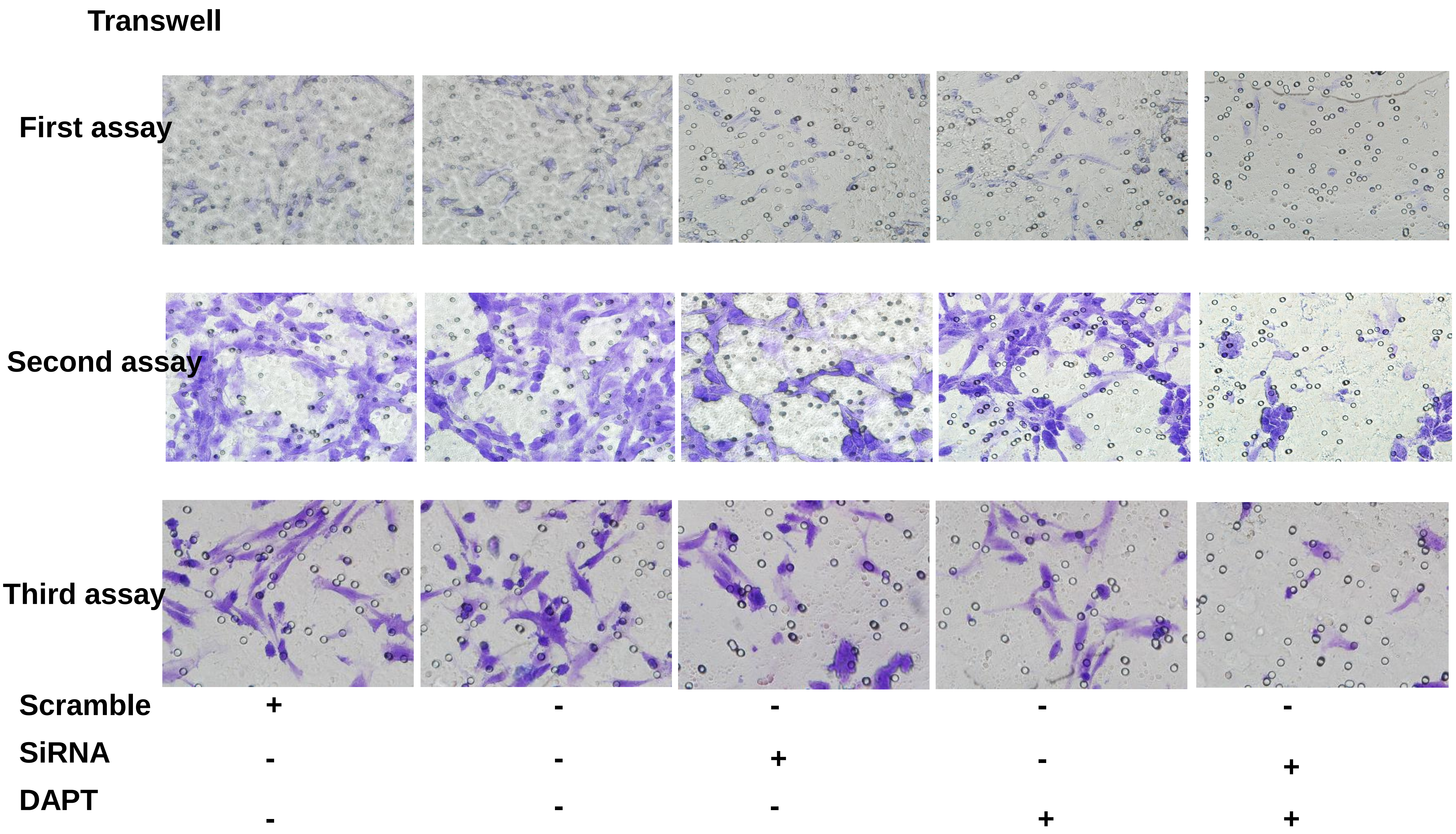
Transwell
First assay
Second assay
Third assay
Scramble + - - - -
SiRNA - - + - +
DAPT - - - + +

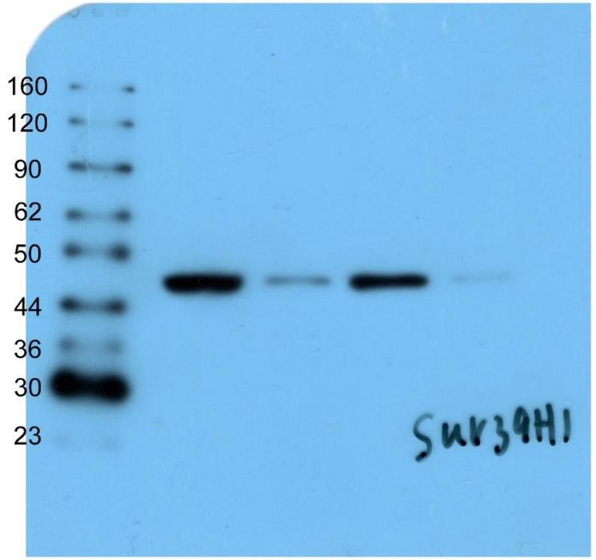
160
120
90
62
50
44
36
30
23
Suv39H1

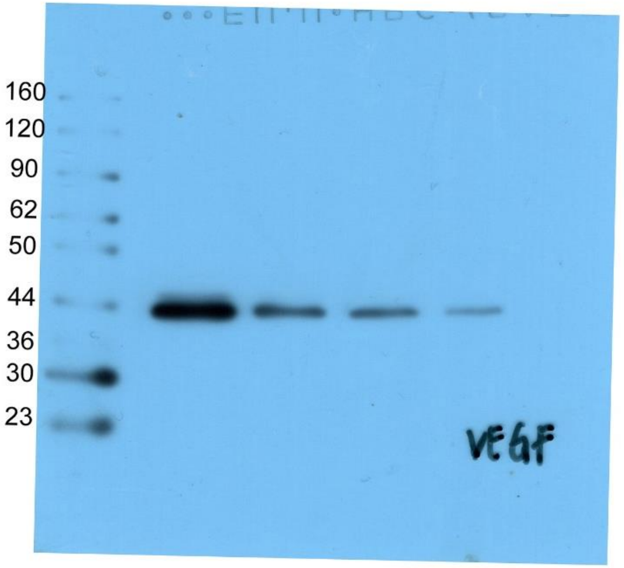
160
120
90
62
50
44
36
30
23
VEGF

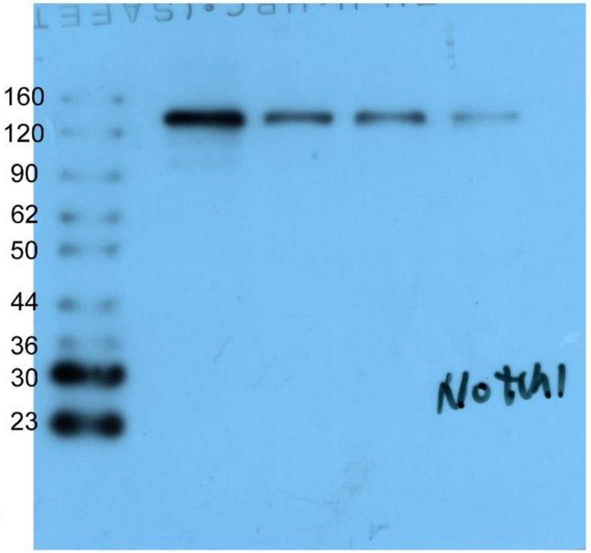
160
120
90
62
50
44
36
30
23
Notch1

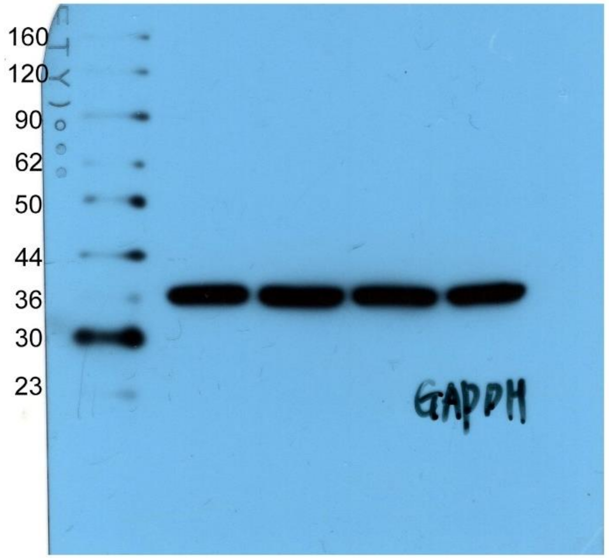
160
120
90
62
50
44
36
30
23
GAPDH

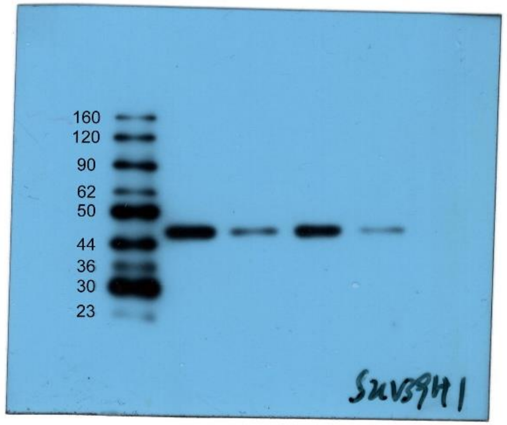
160
120
90
62
50
44
36
30
23
Suv39H1

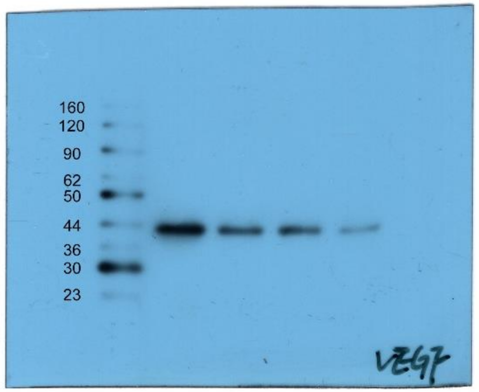
160
120
90
62
50
44
36
30
23
VEGF

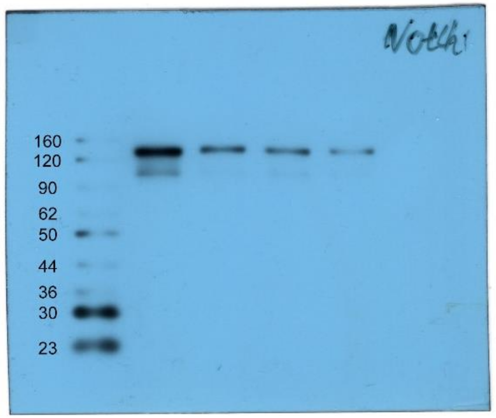
Notch1
160
120
90
62
50
44
36
30
23

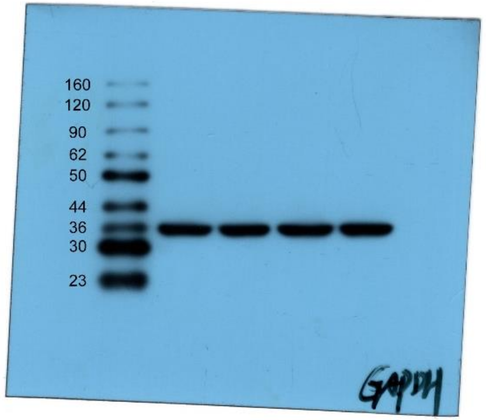
160
120
90
62
50
44
36
30
23
GAPDH
