# Supplementary material for: Inhibition of SUV39H1 reduces tumor angiogenesis via Notch1 in oral squamous cell carcinoma
Source: PeerJ. 2024 Apr 19;12:e17222. doi: 10.7717/peerj.17222 (PMC11034493; doi:10.7717/peerj.17222)

Vehicle **Chaetocin**

0 day

18 days

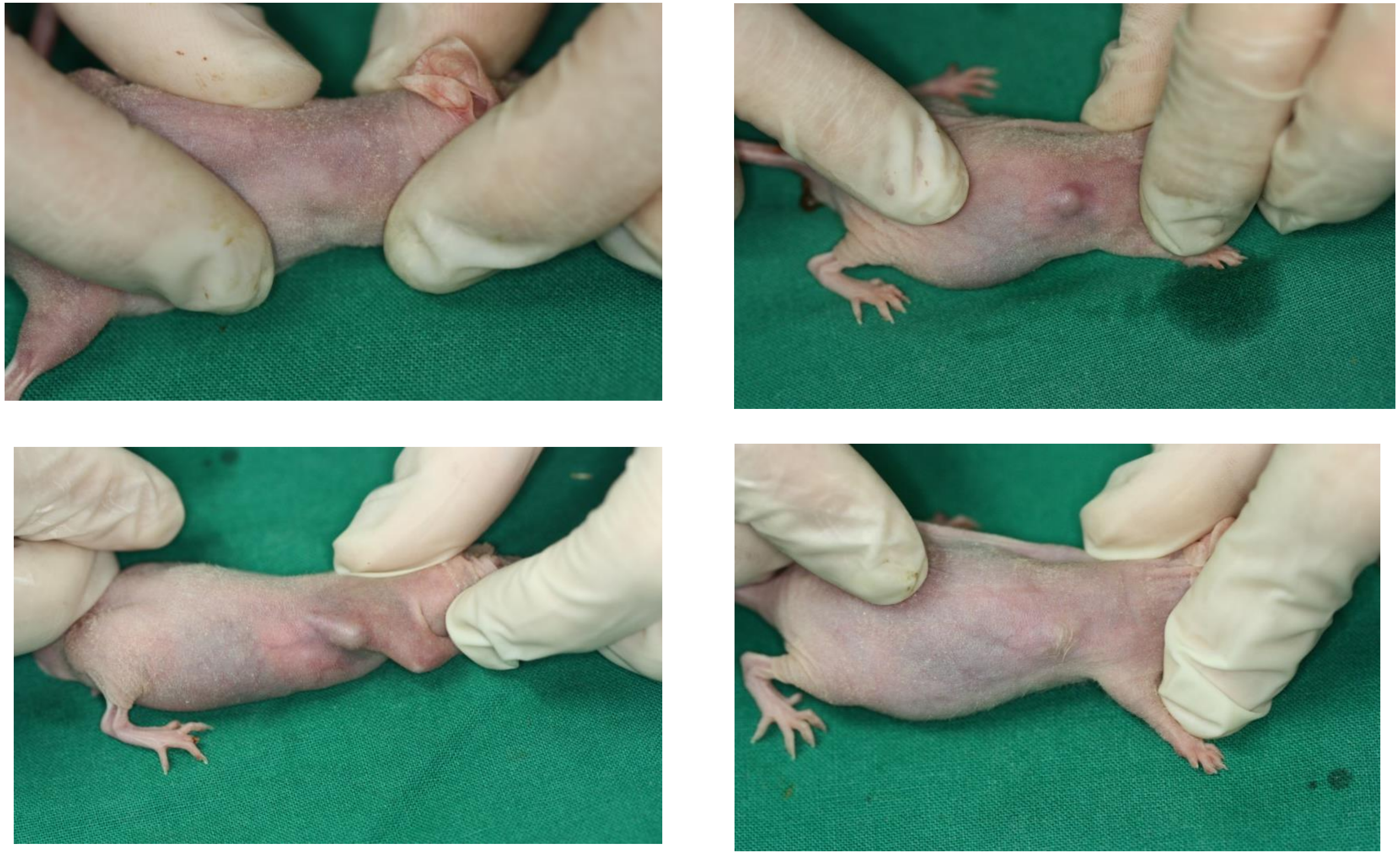

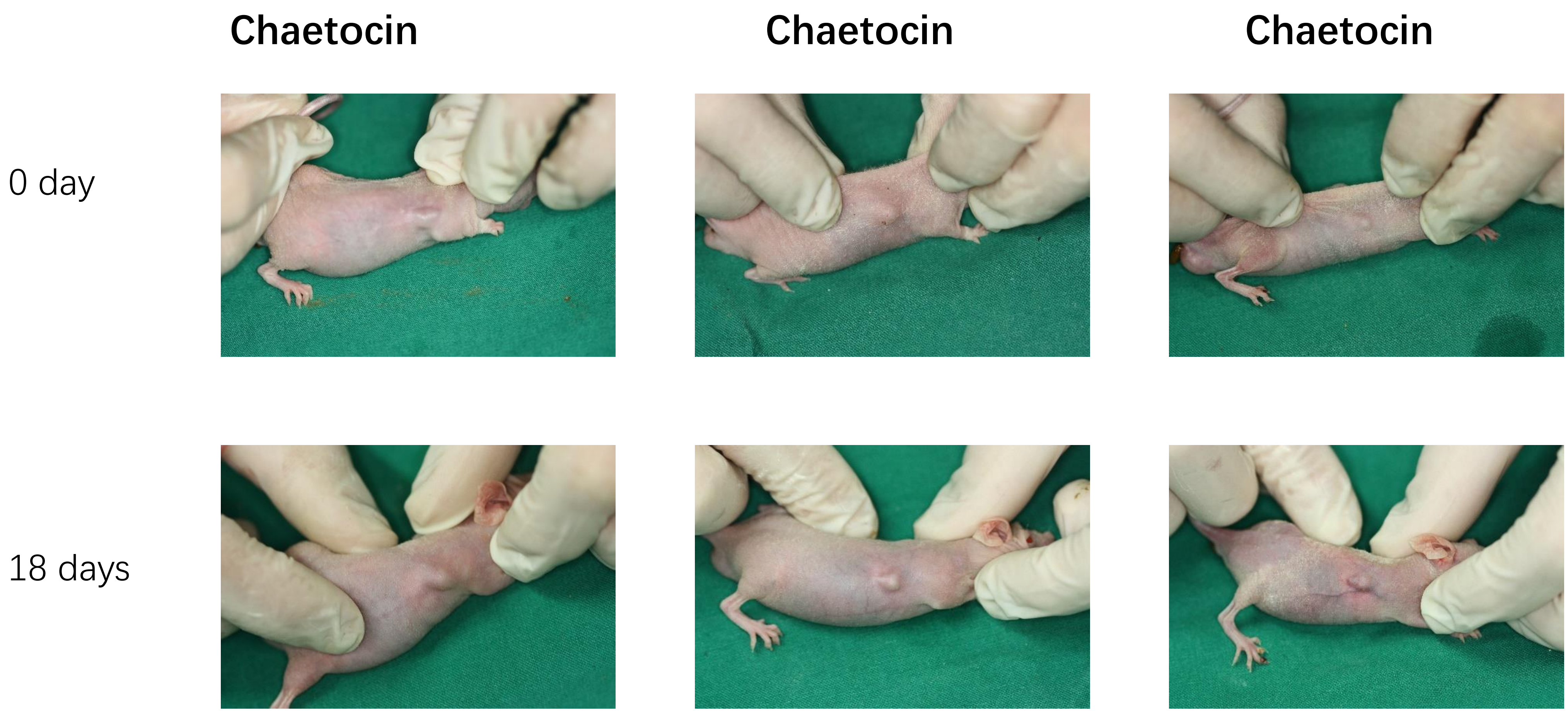
Chaetocin
Chaetocin
Chaetocin
0 day
18 days

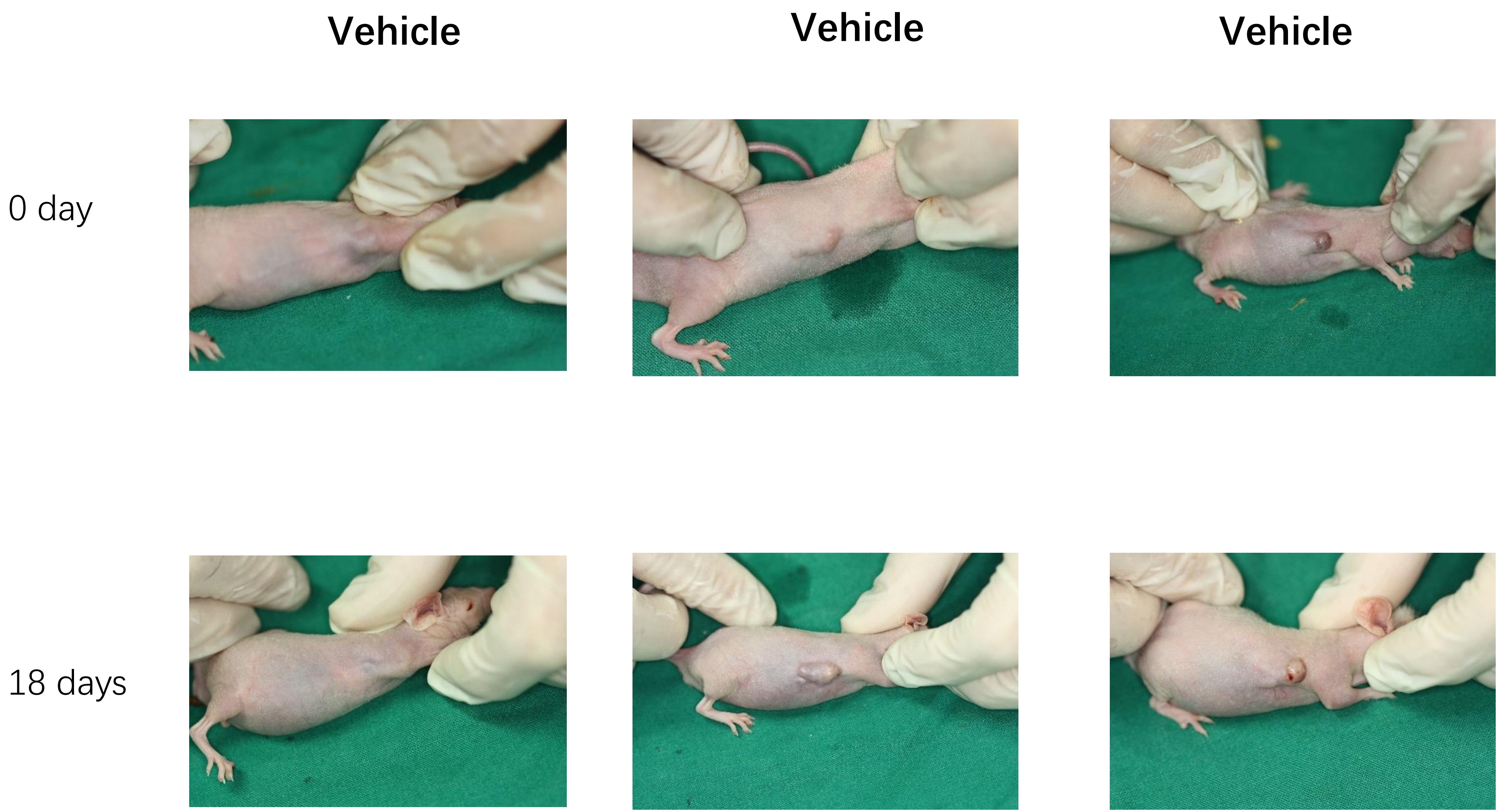
Vehicle
Vehicle
Vehicle
0 day
18 days

SUV39H1 Vehicle

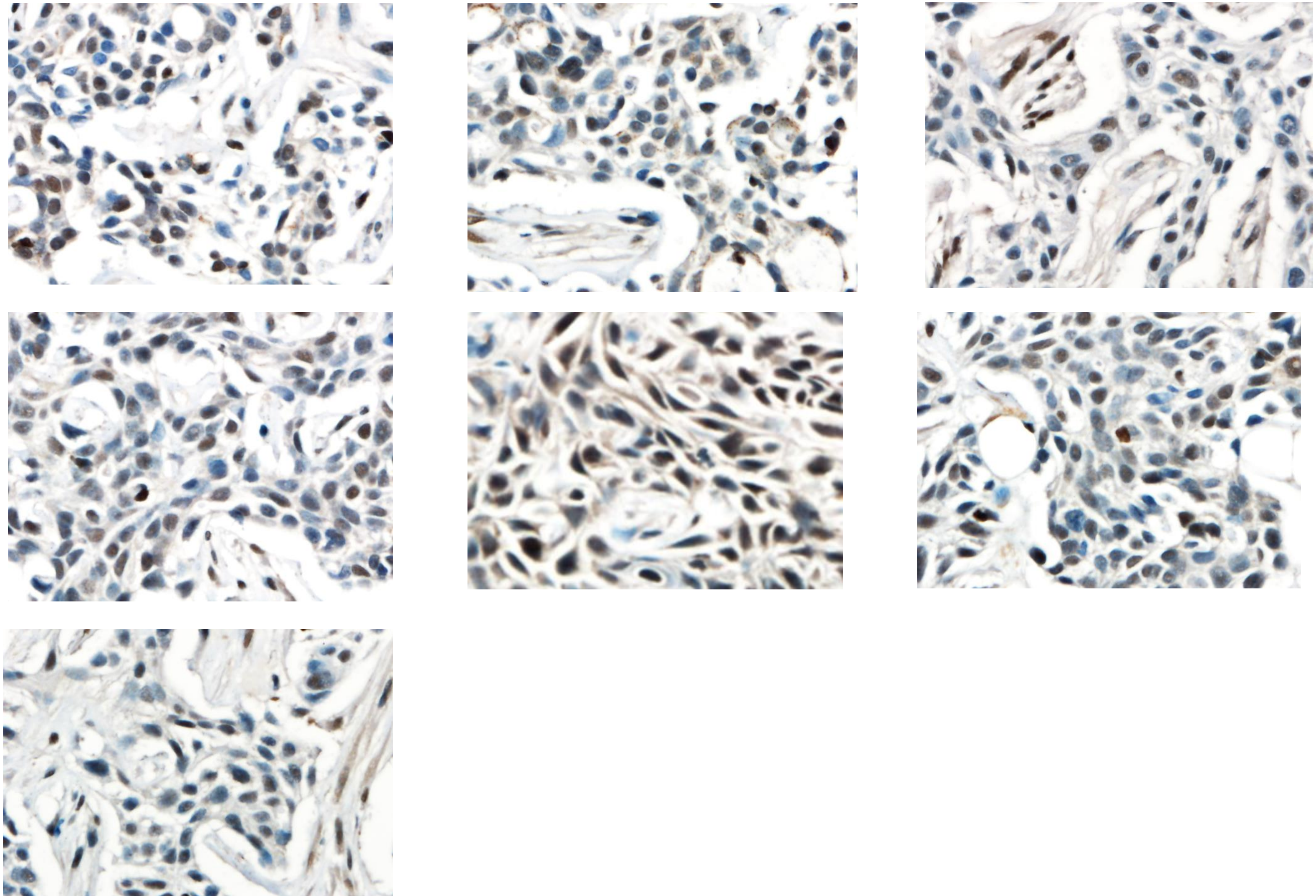

SUV39H1 Chaetocin

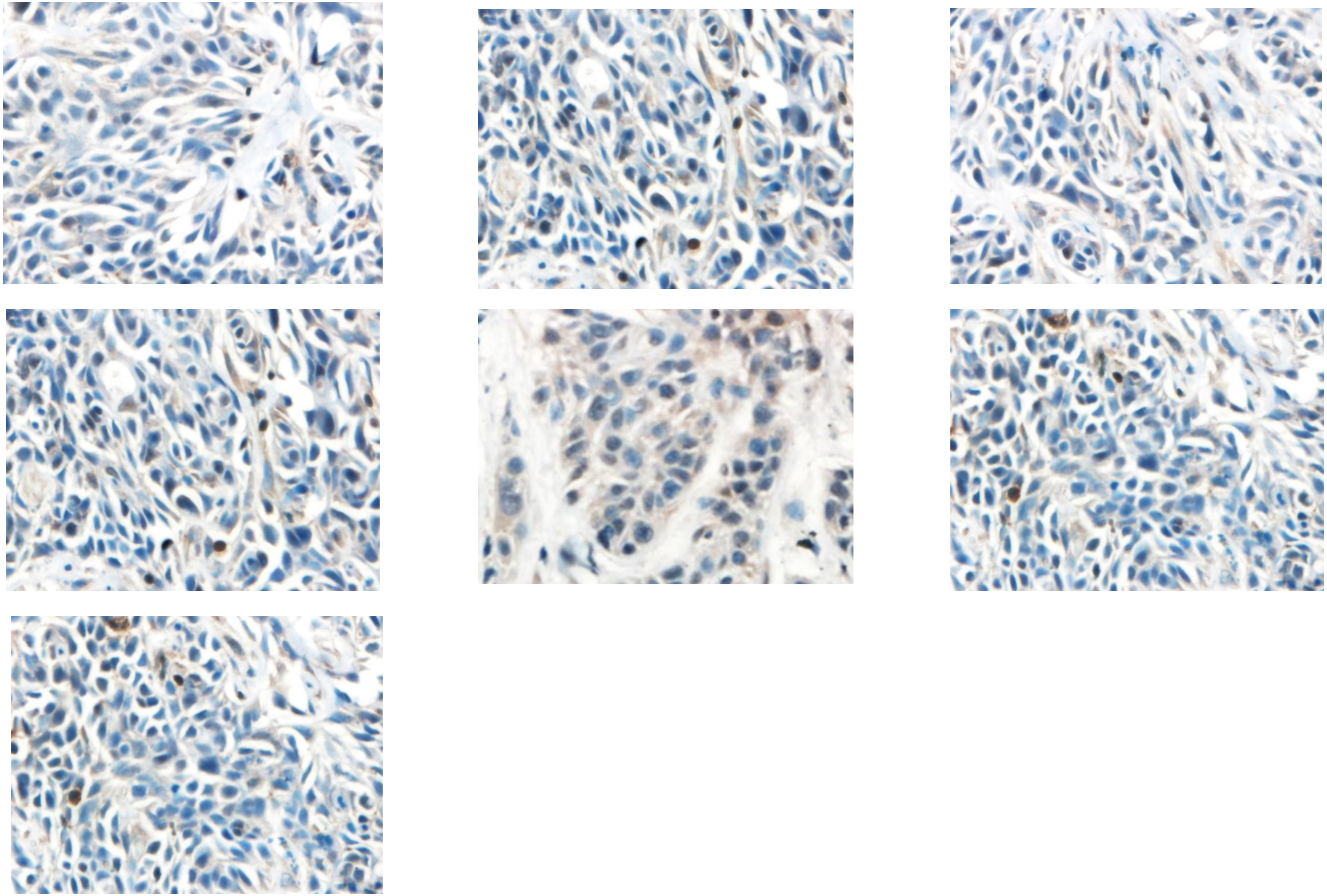

CD31 Vehicle

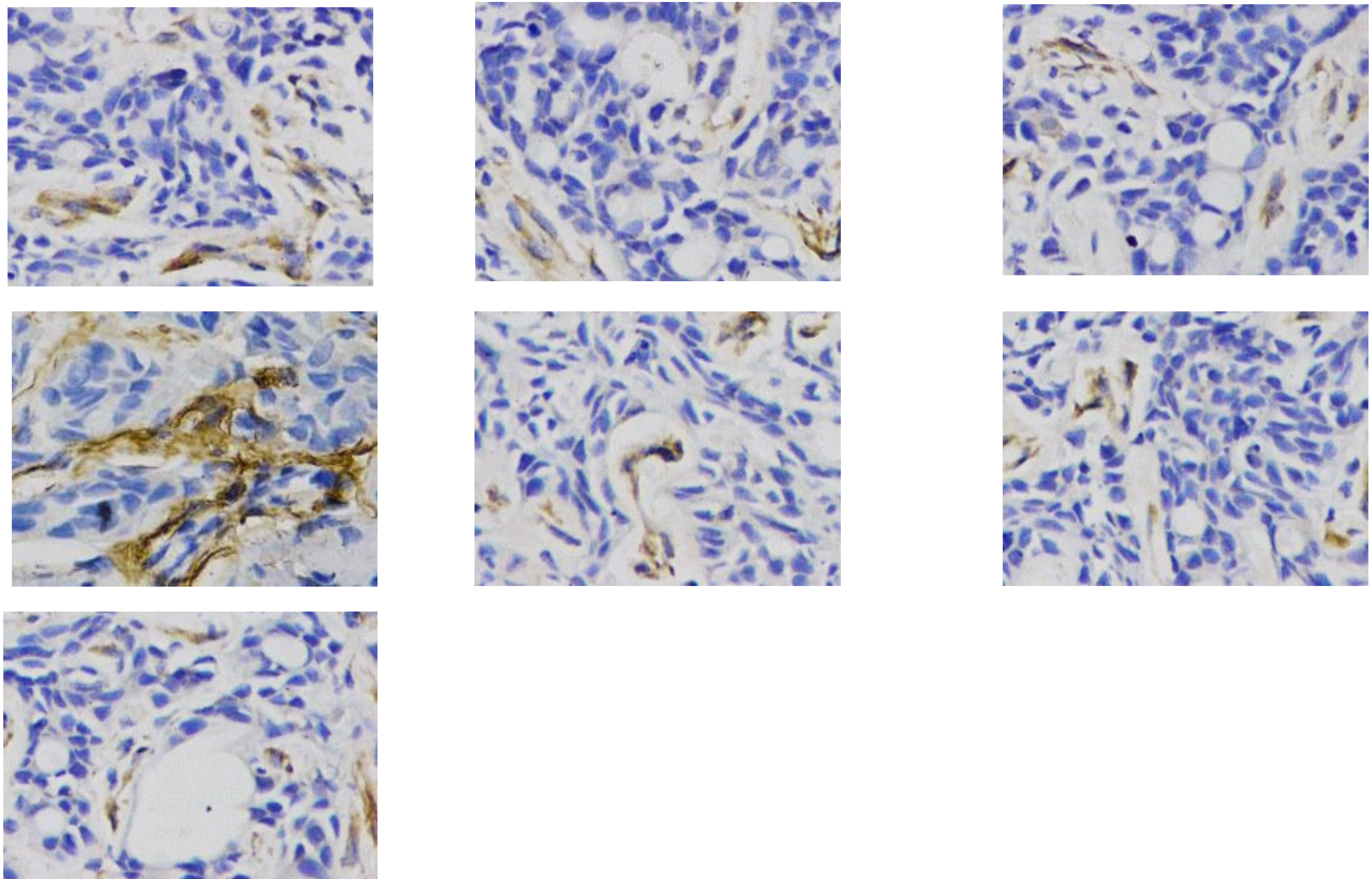

CD31 Chaetocin

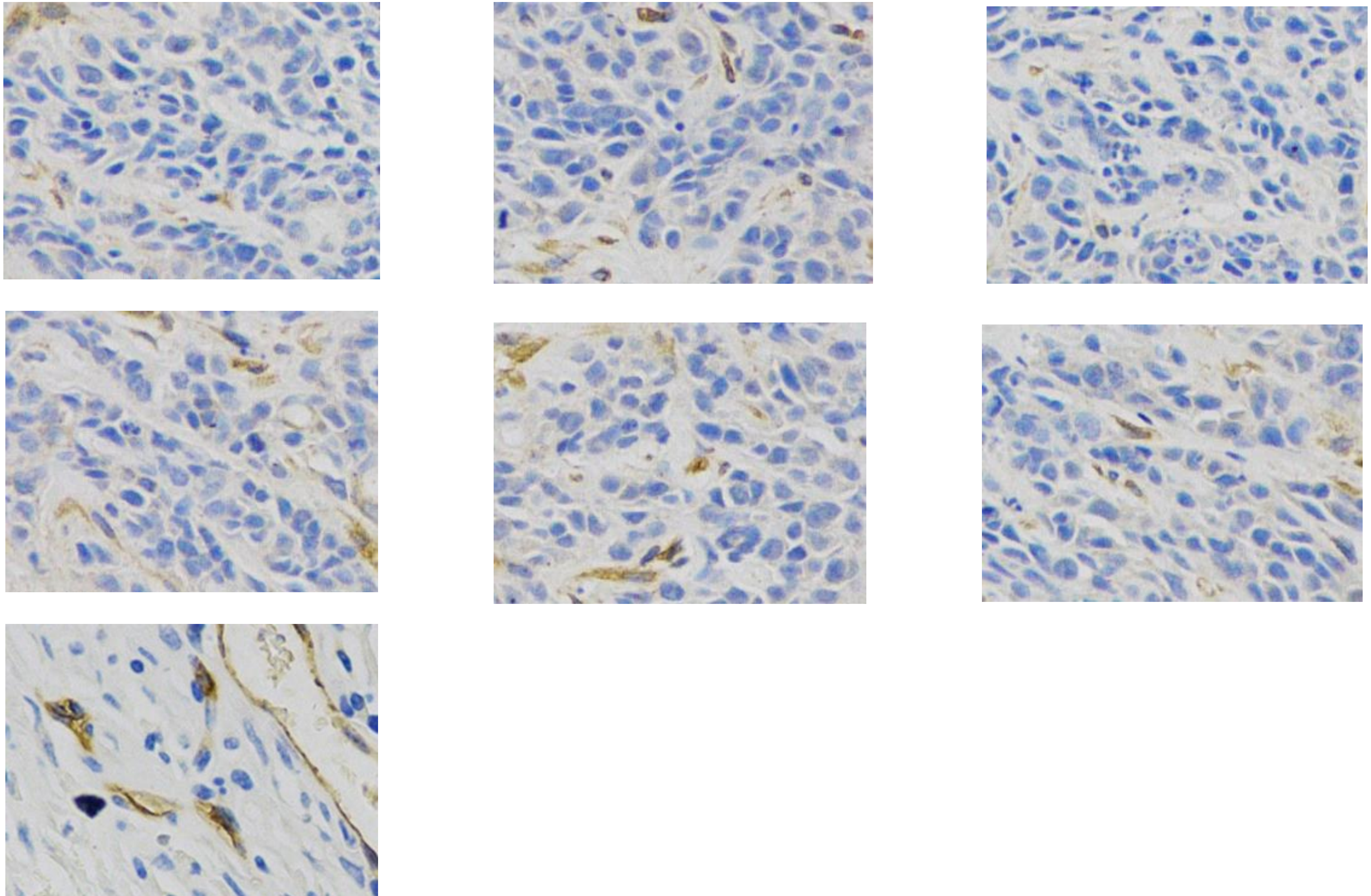

NOTCH1 Vehicle

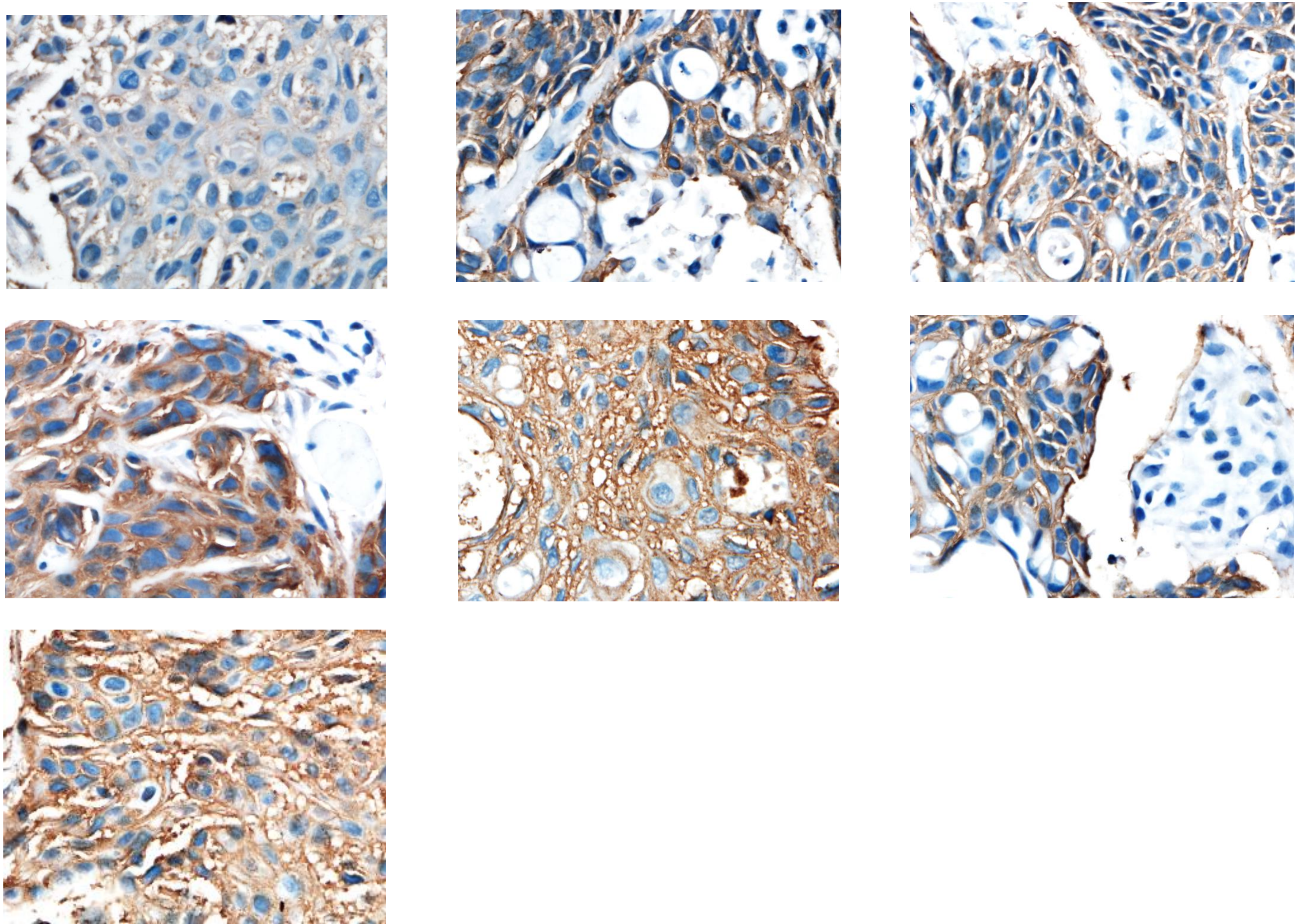

NOTCH1 Chaetocin

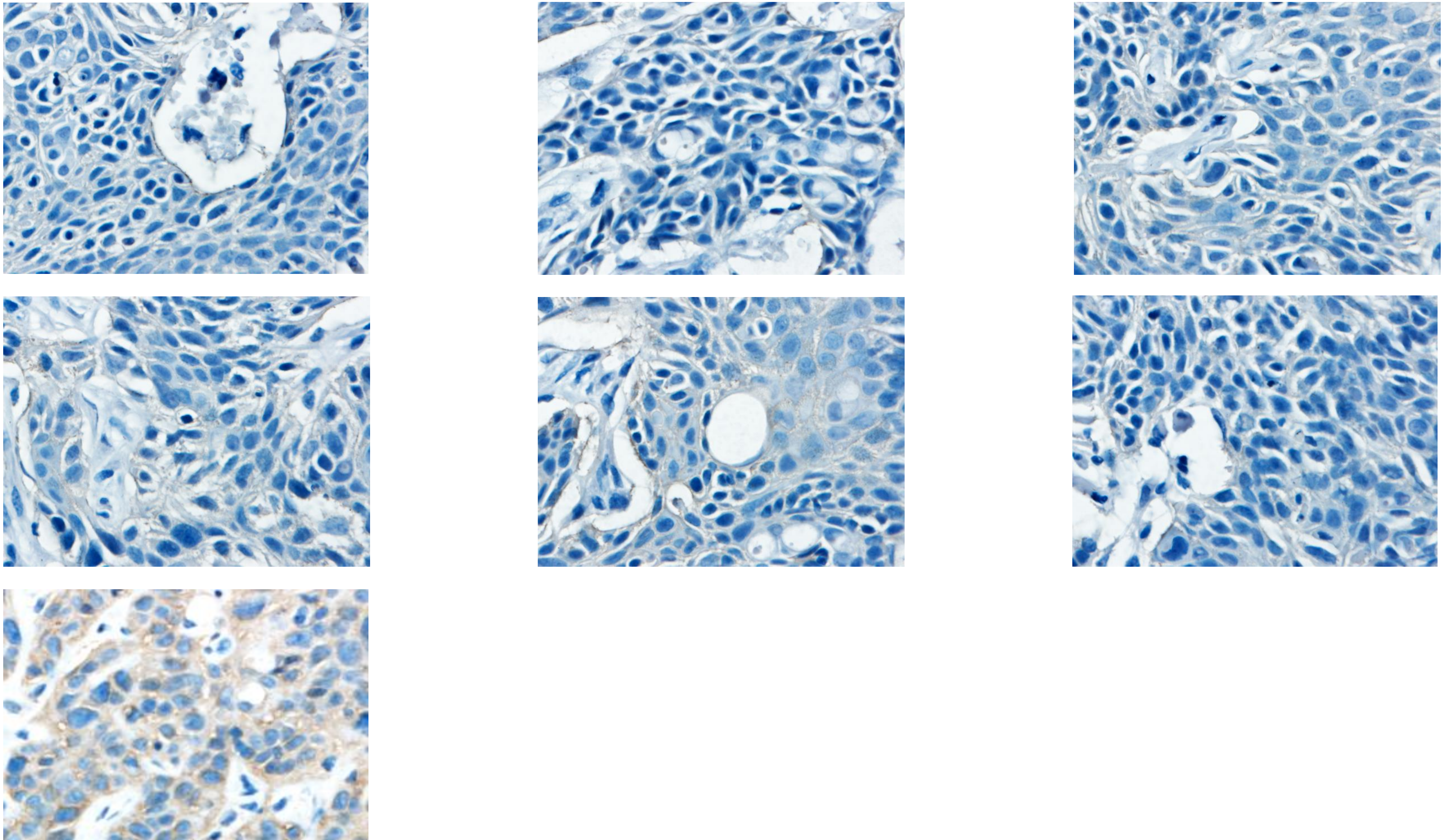

Ki67 vehicle

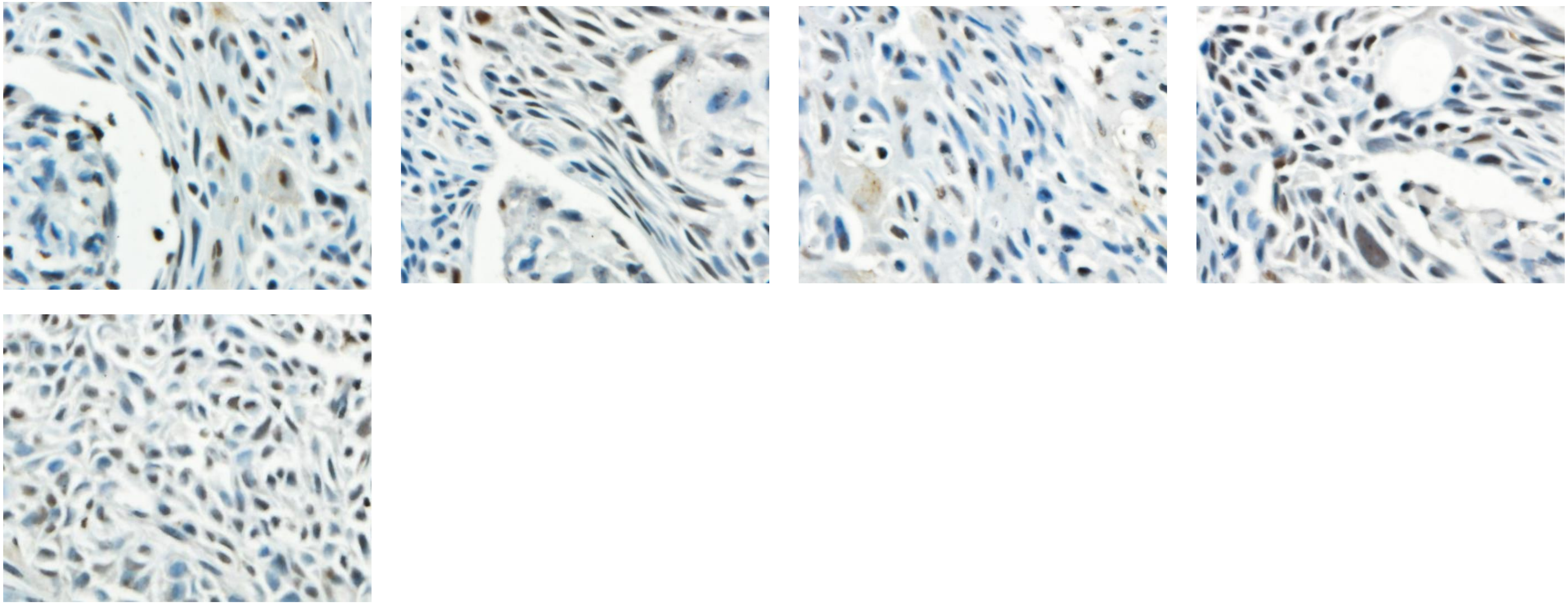

Ki67 Chaetocin

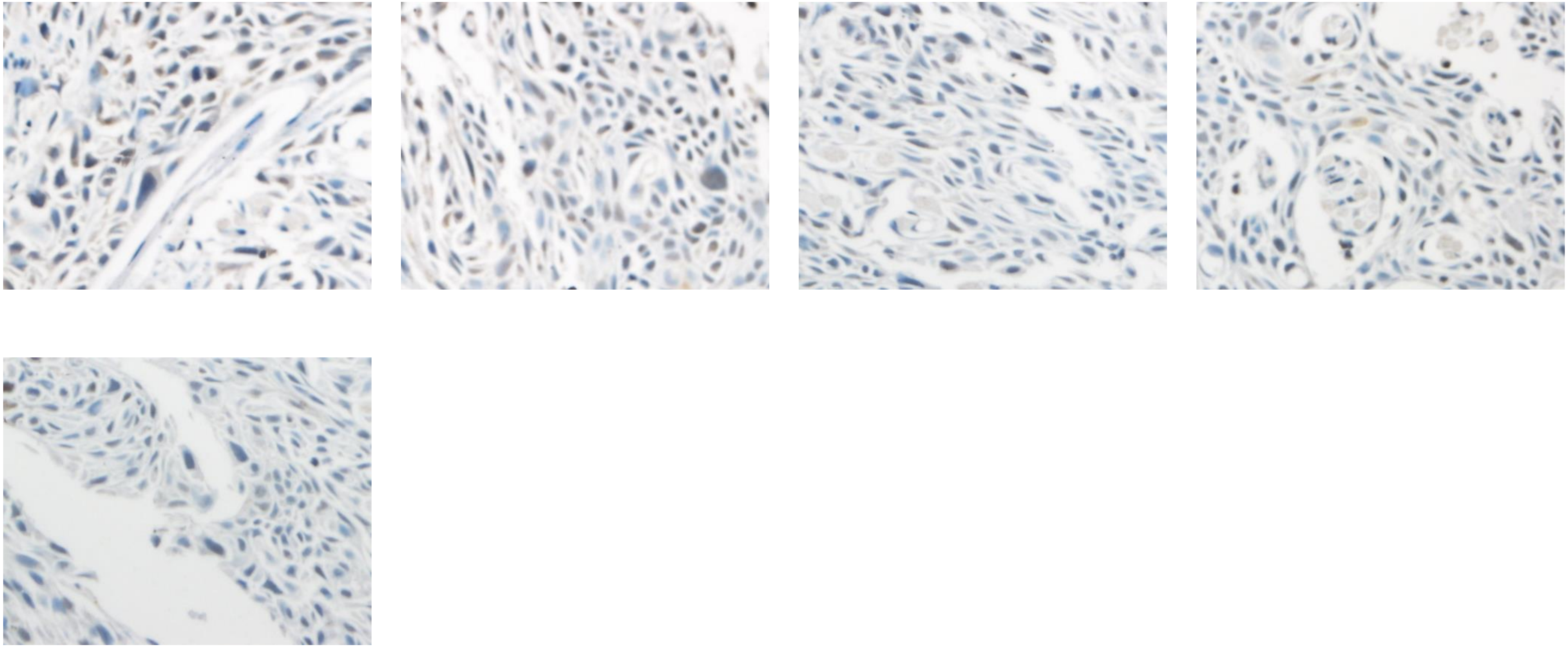

Supplement: Supplemental Information 5 [file peerj-12-17222-s005.pdf]
