# Supplementary material for: Inhibition of SUV39H1 reduces tumor angiogenesis via Notch1 in oral squamous cell carcinoma
Source: PeerJ. 2024 Apr 19;12:e17222. doi: 10.7717/peerj.17222 (PMC11034493; doi:10.7717/peerj.17222)

SUV39H1 Figure1 raw picture

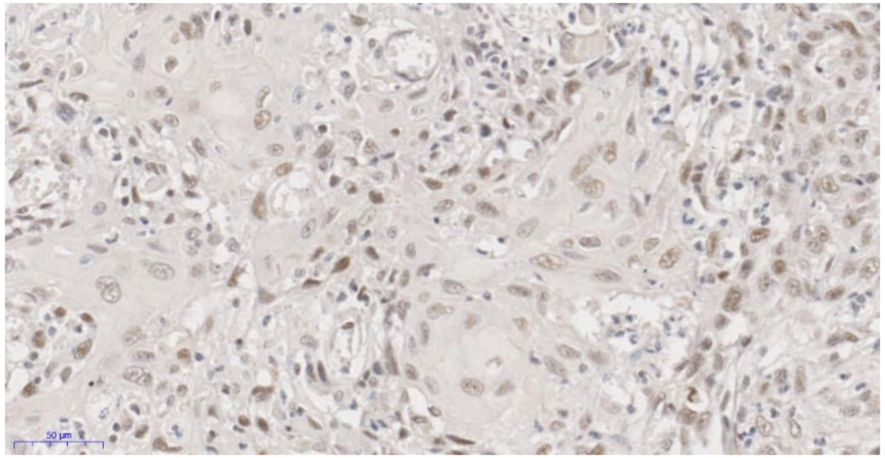

OSCC

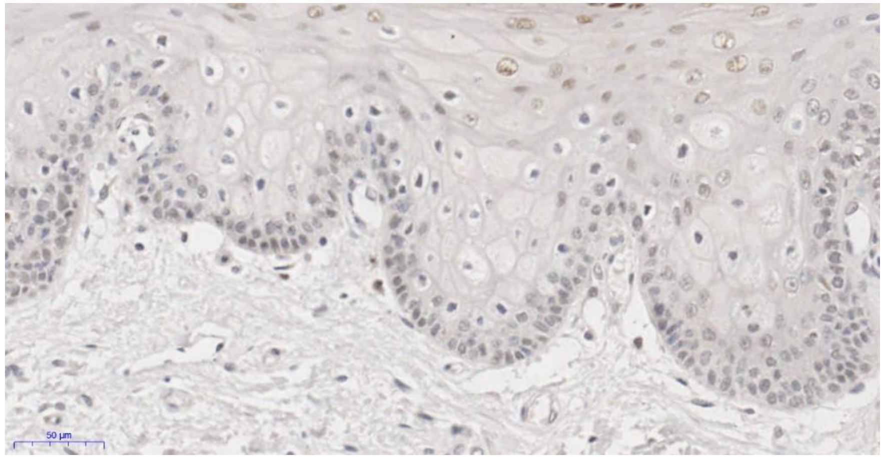

NM

Figure2 raw picture

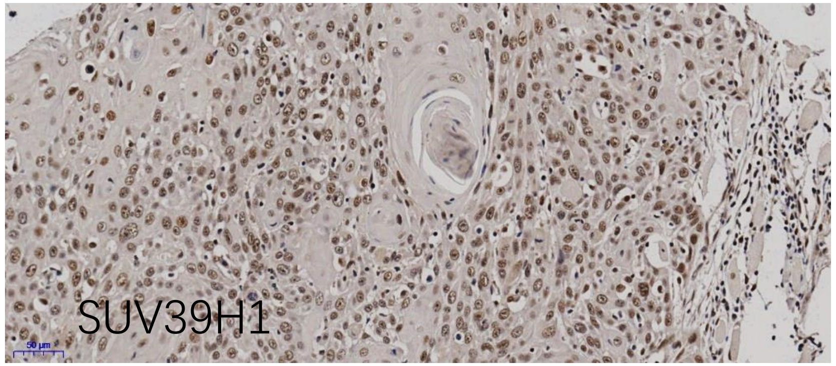

OSCC

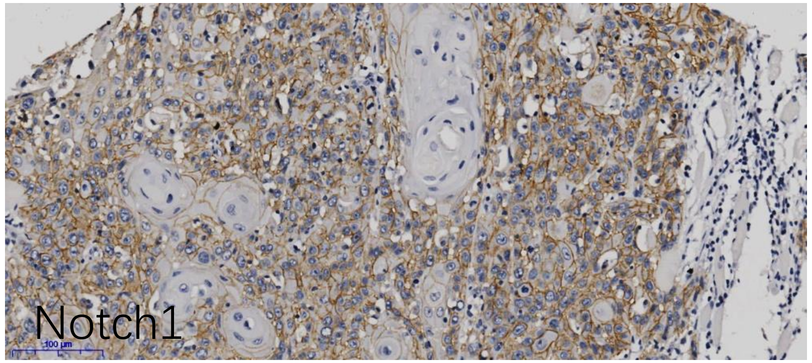

OSCC

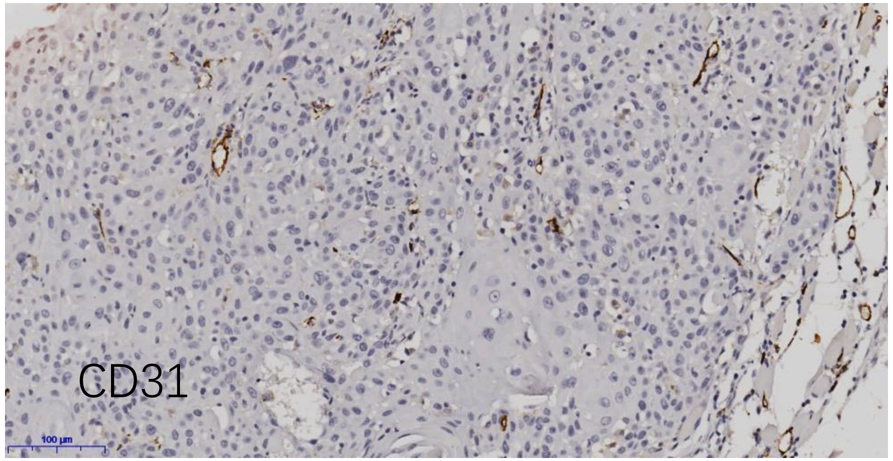

OSCC

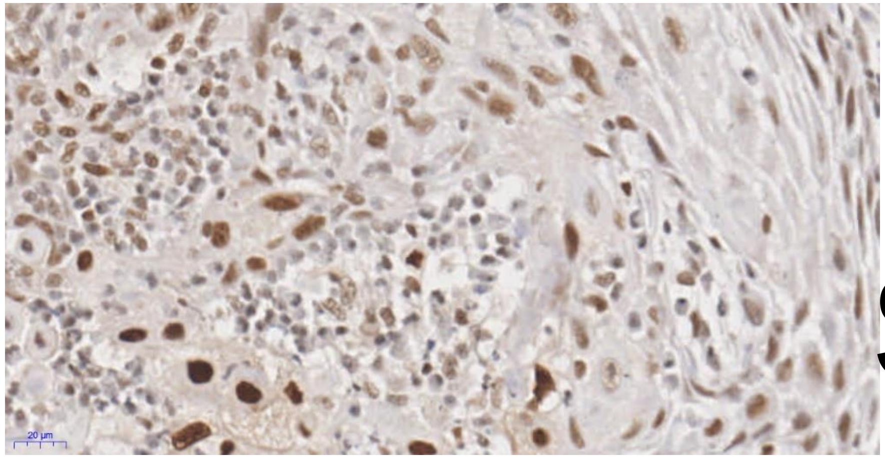

OSCC
SUV39H1

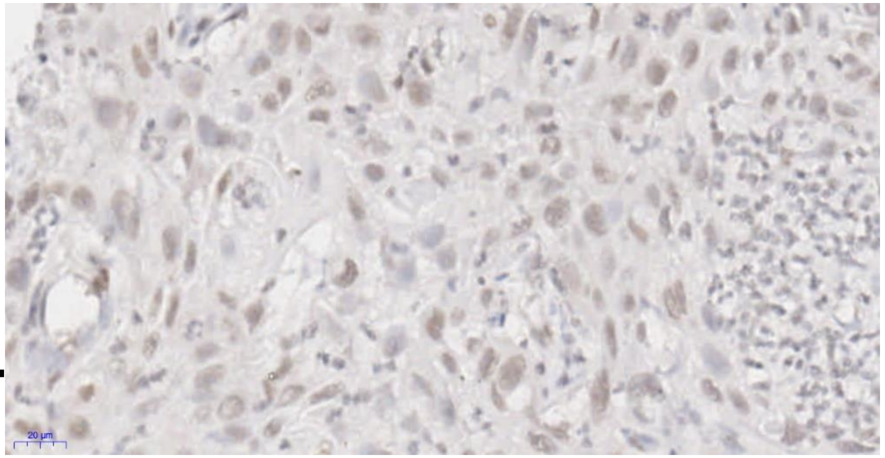

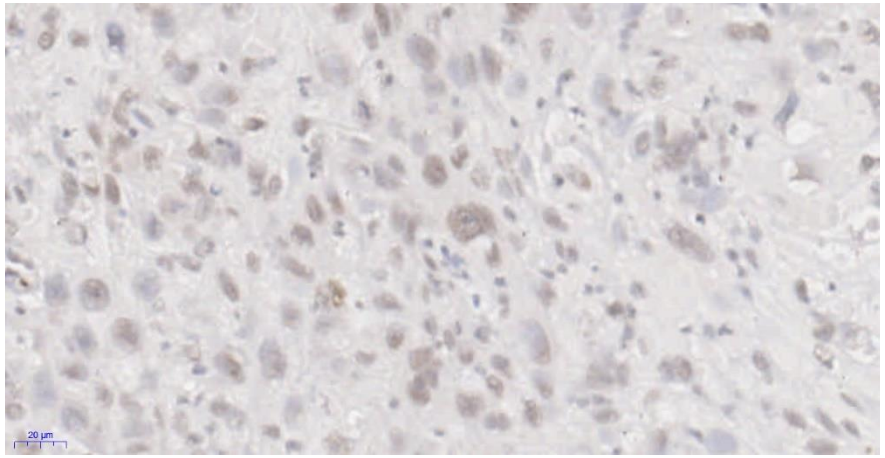

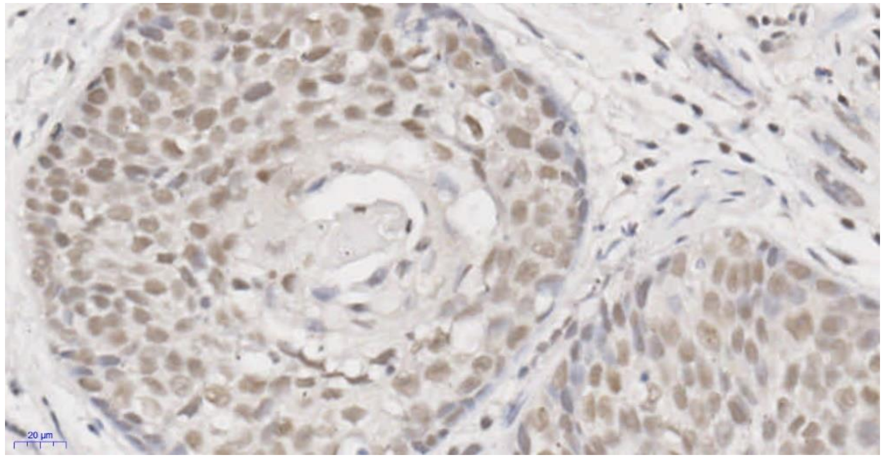

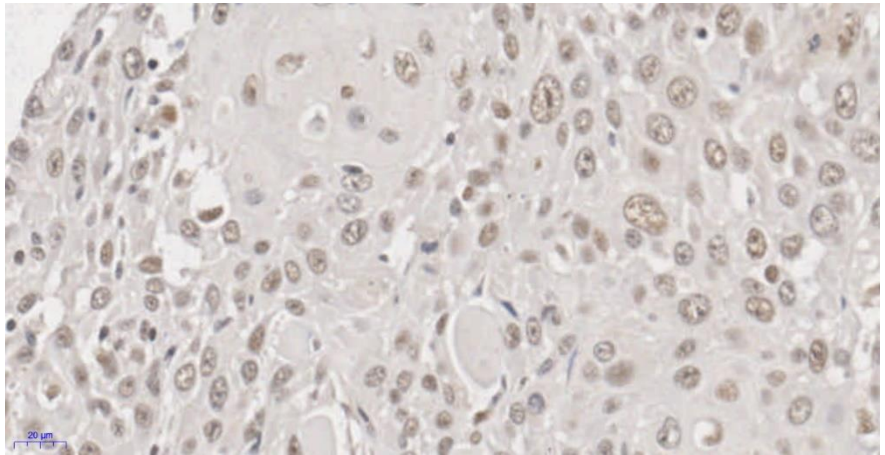

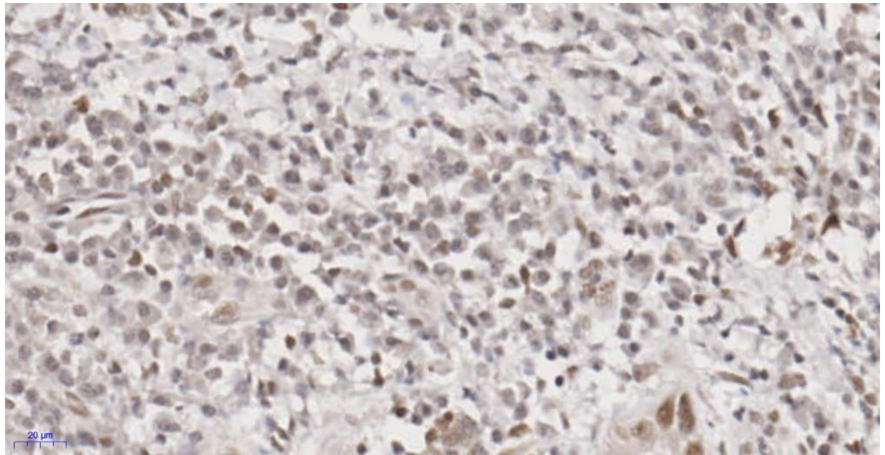

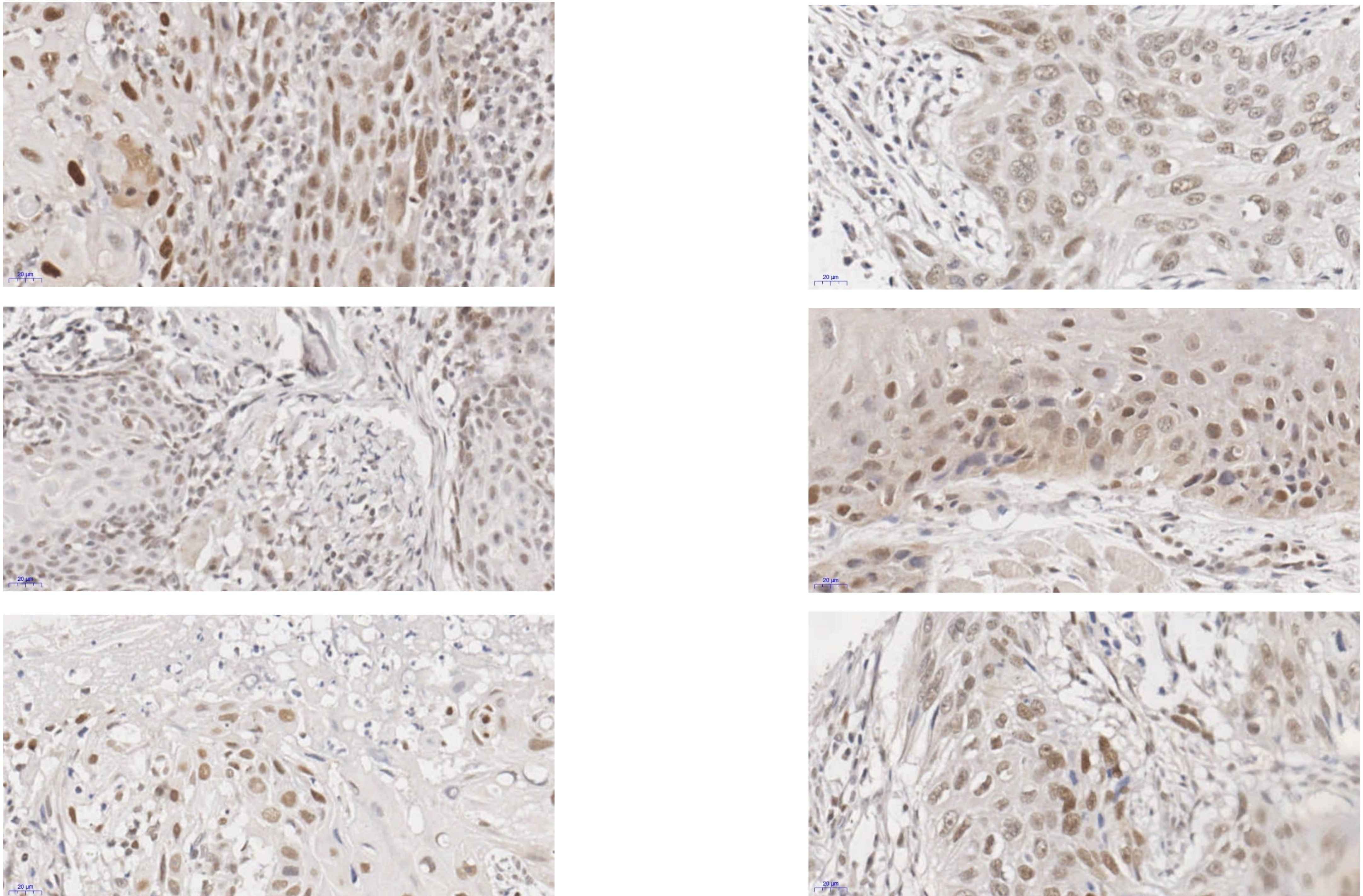
20 µm
20 µm
20 µm
20 µm
20 µm
20 µm

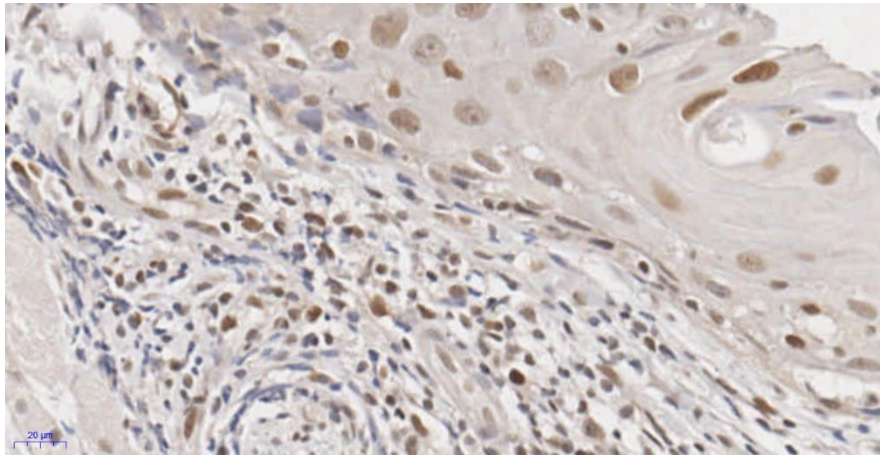
20 µm
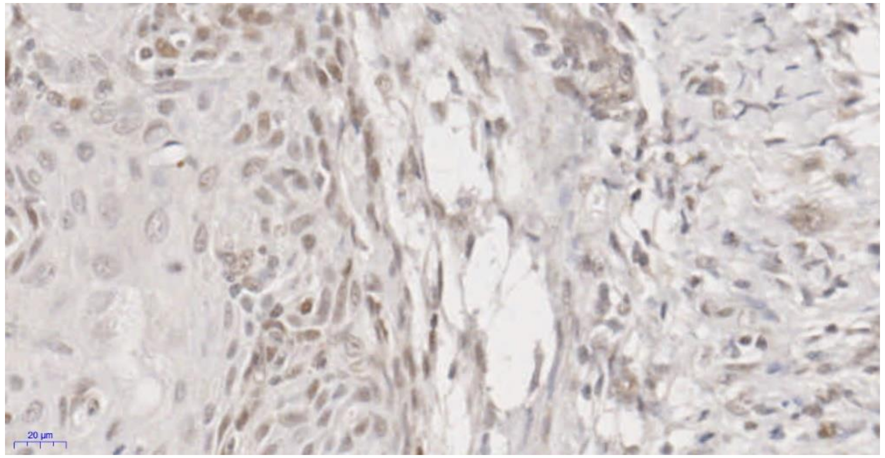
20 µm
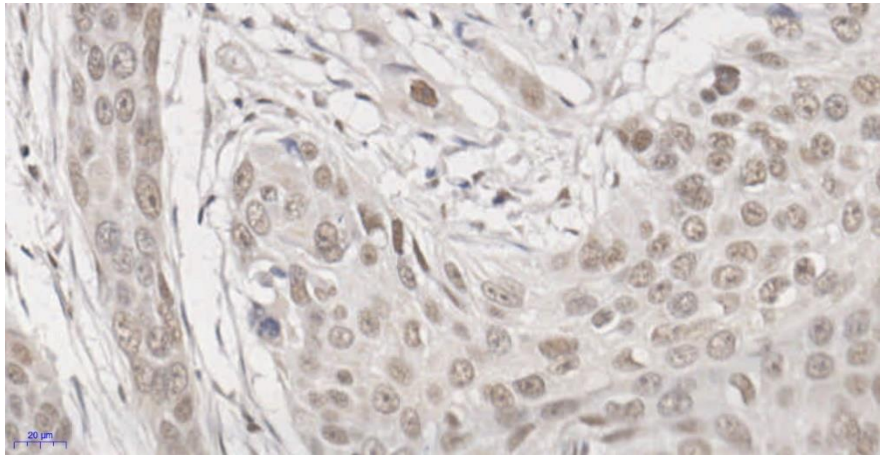
20 µm
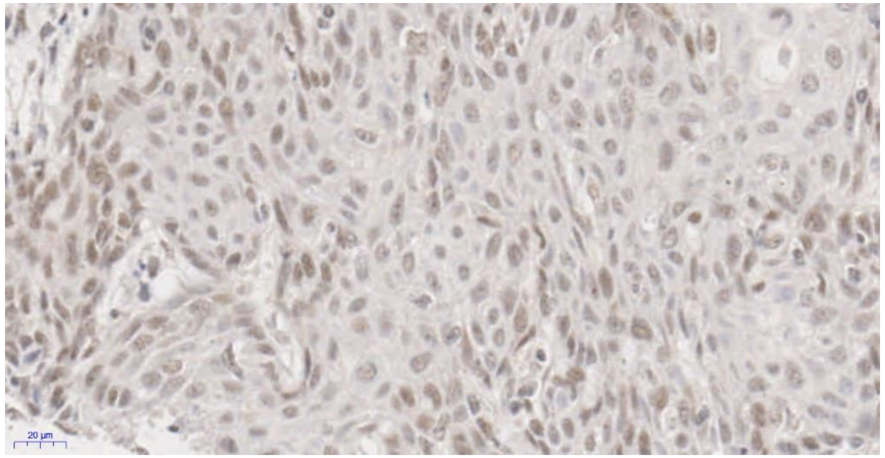
20 µm
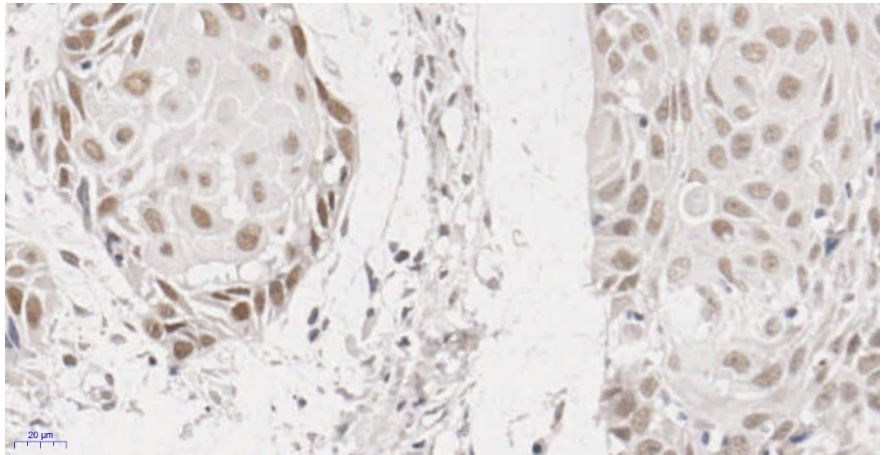
20 µm
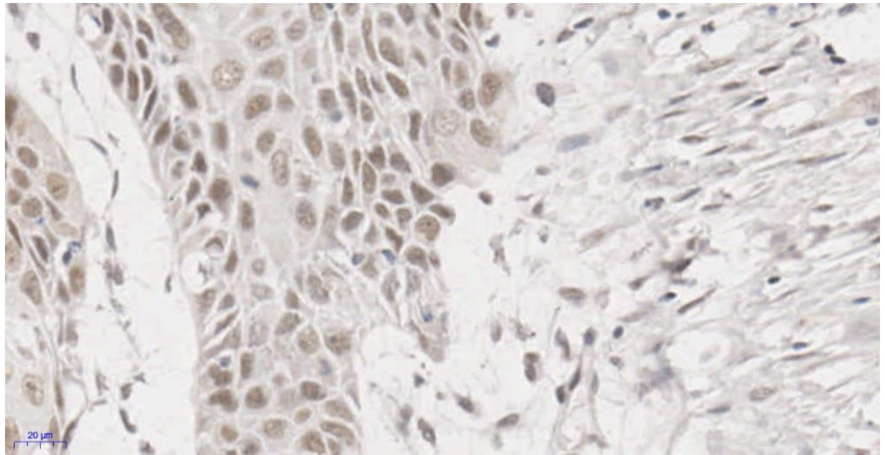
20 µm

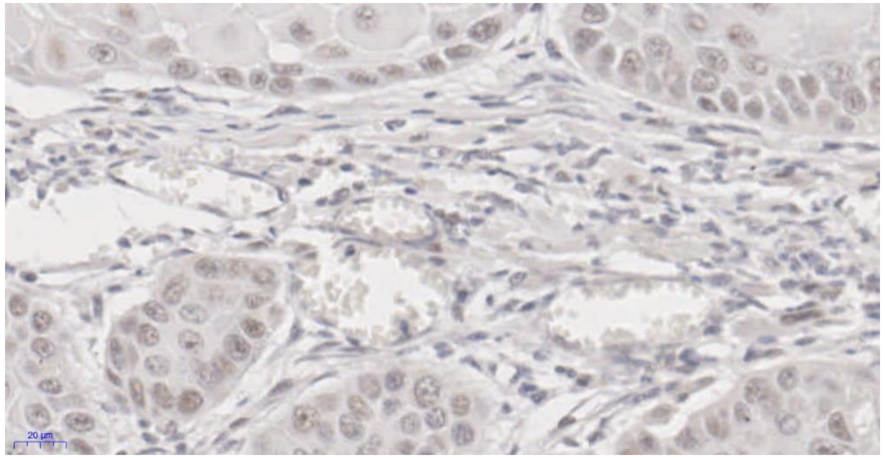
20 µm
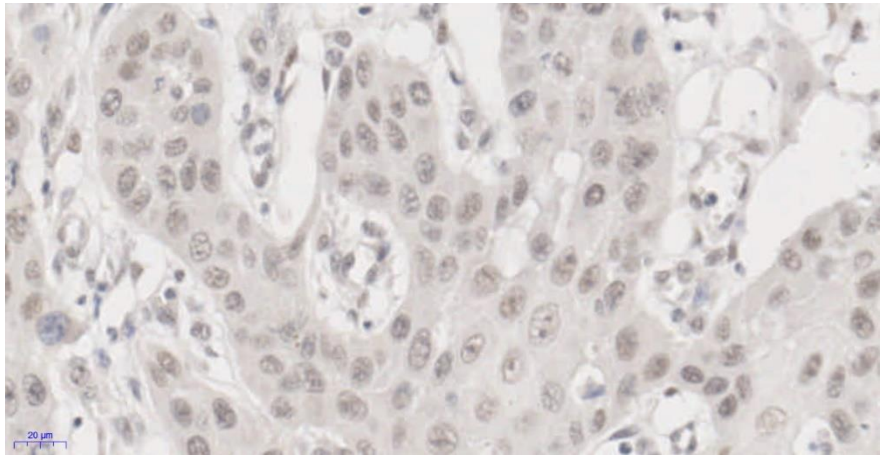
20 µm
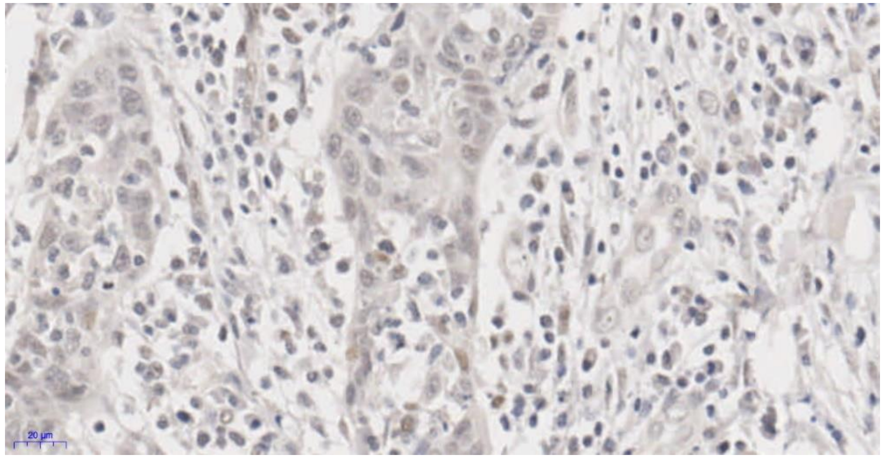
20 µm
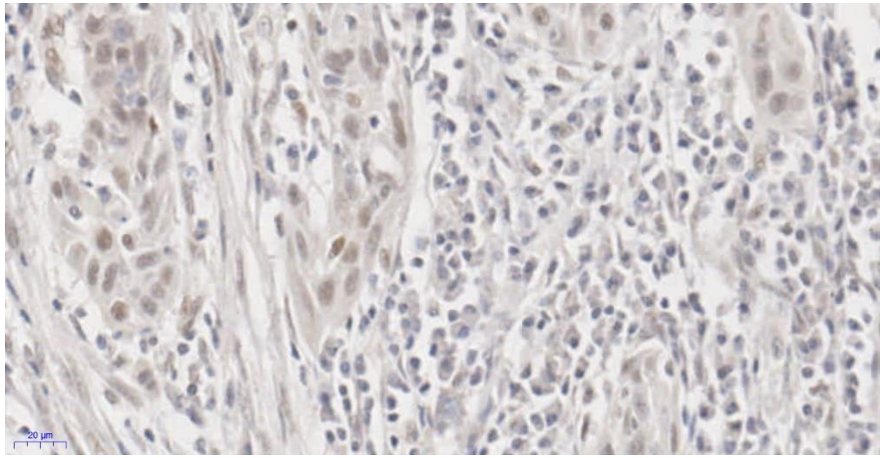
20 µm
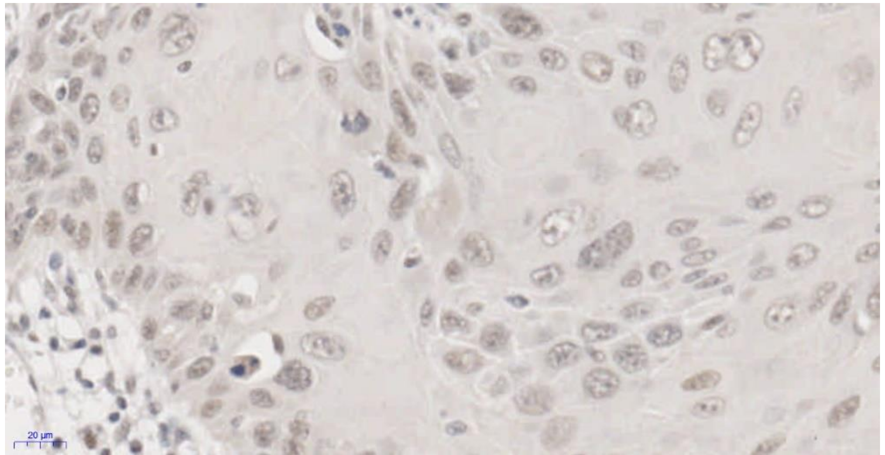
20 µm
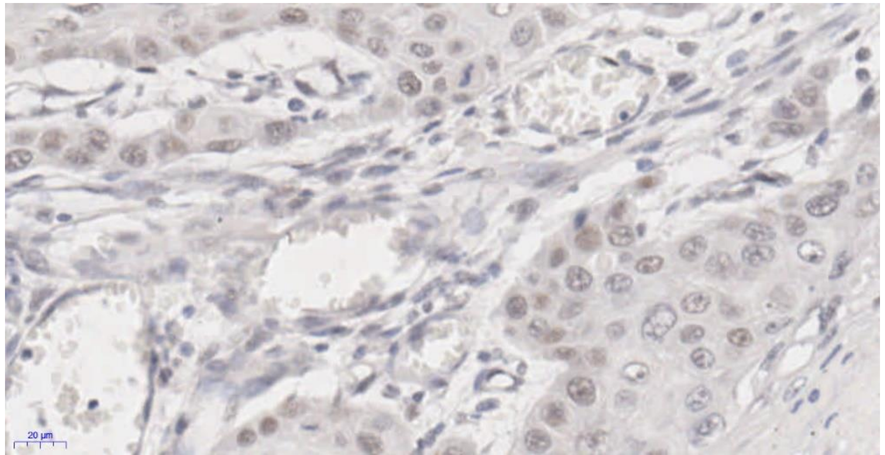
20 µm

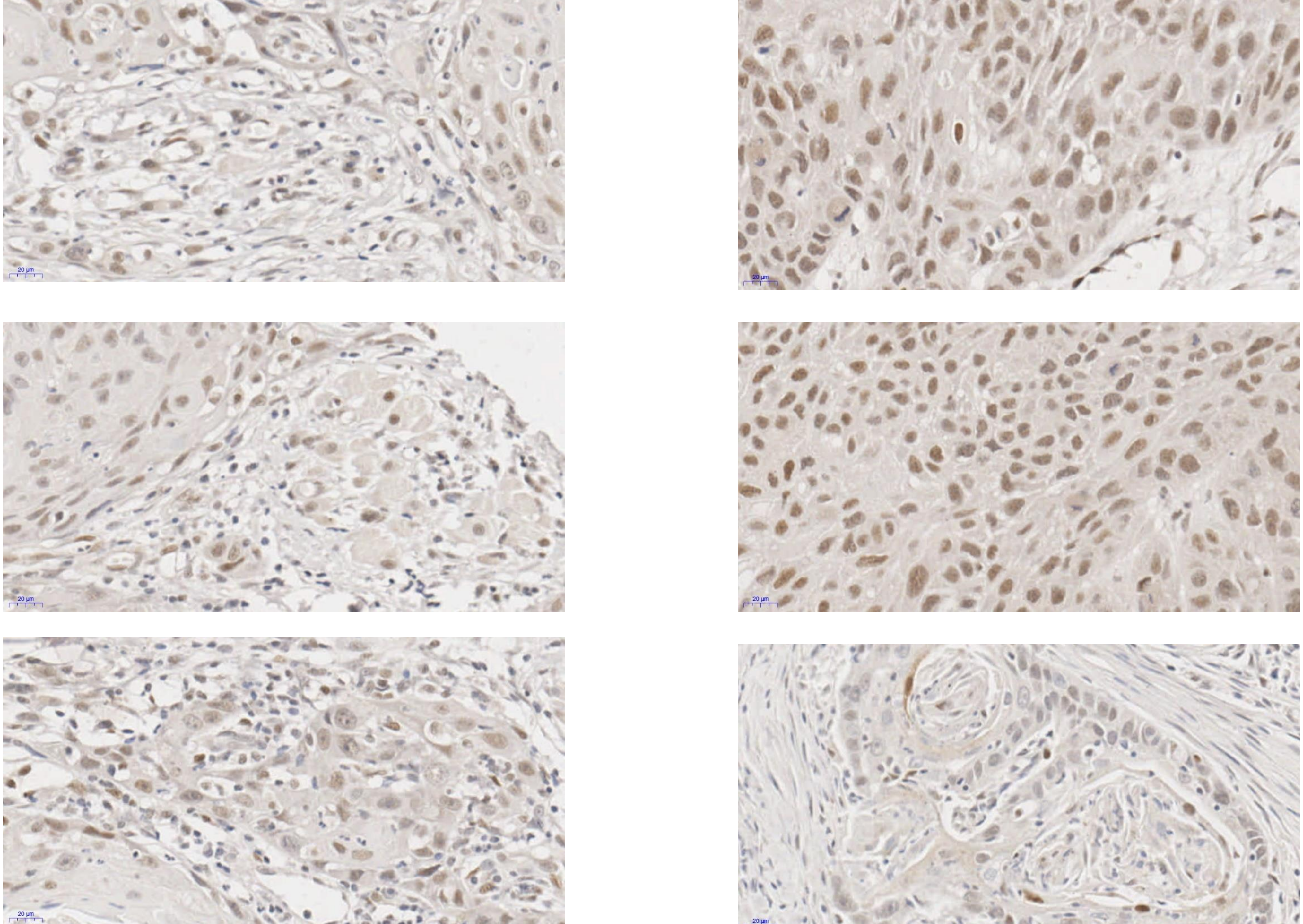
20 μm
20 μm
20 μm
20 μm
20 μm
20 μm

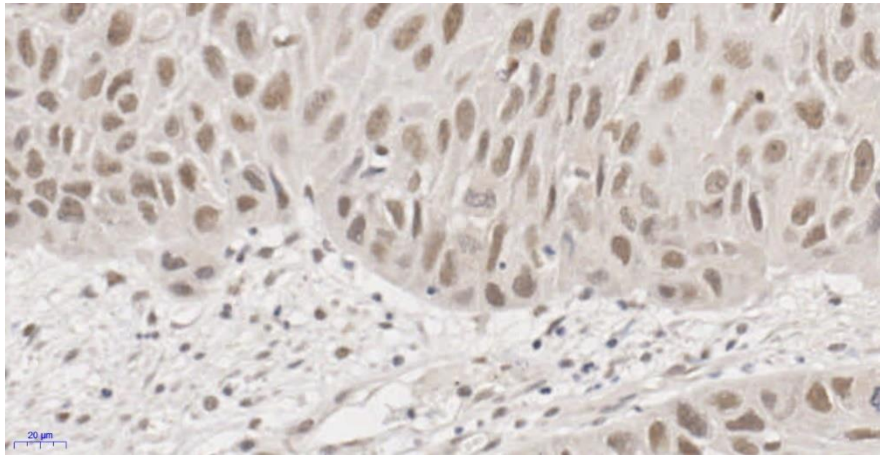
20 µm

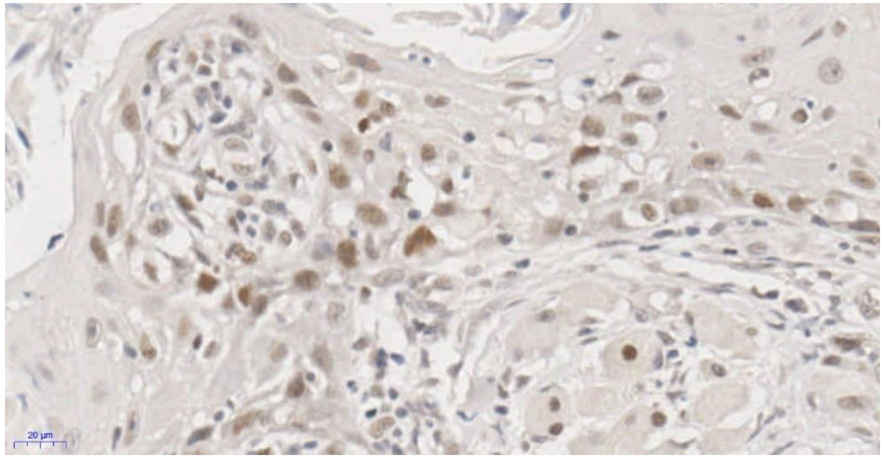
20 µm

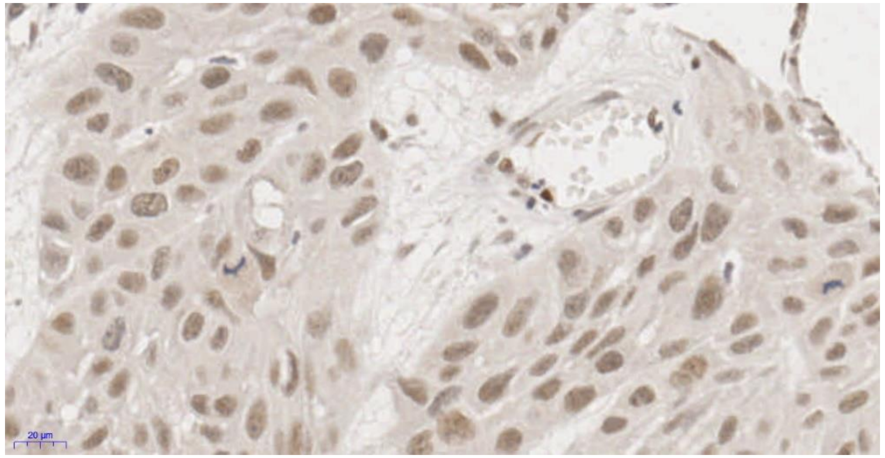
20 µm

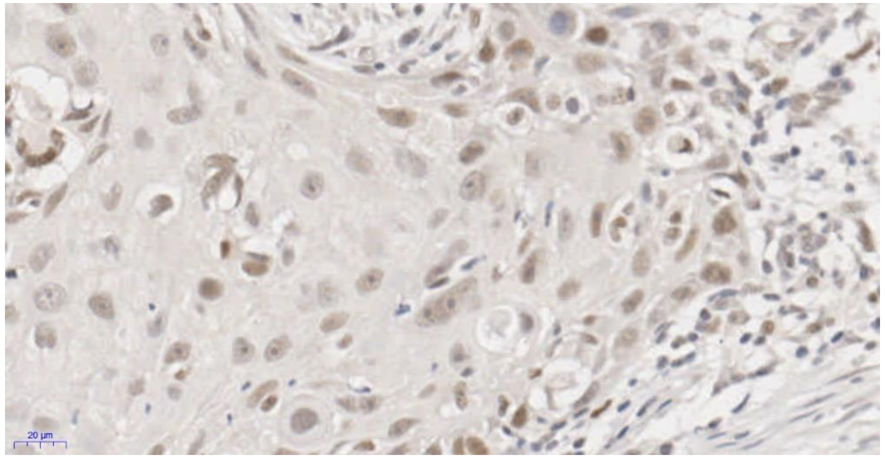
20 µm

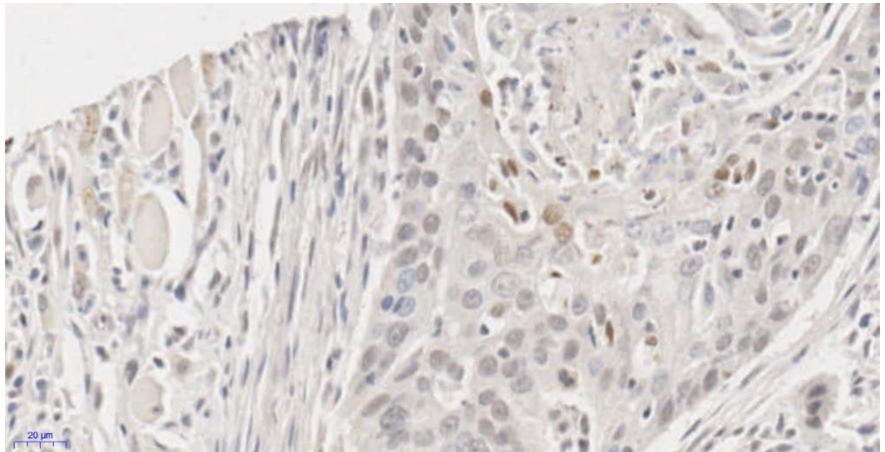
20 µm

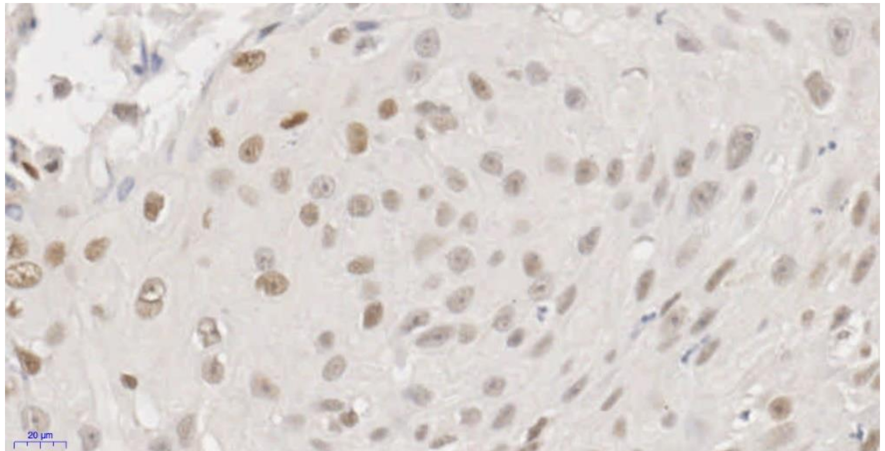
20 µm

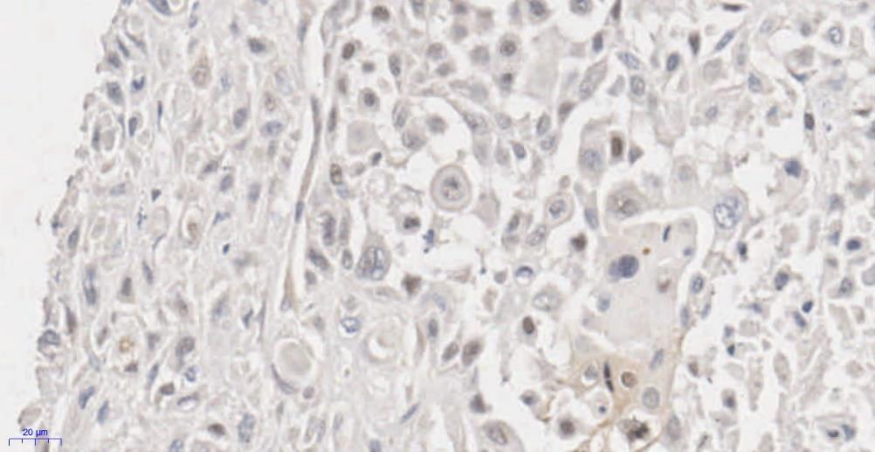
20 µm
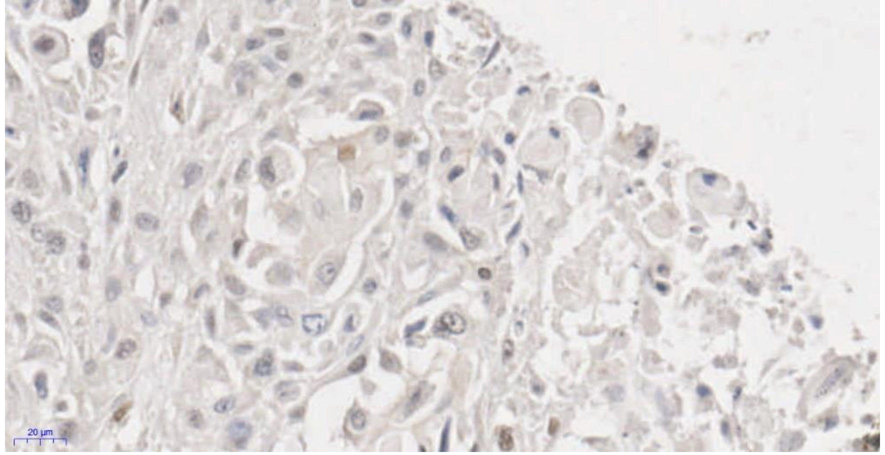
20 µm
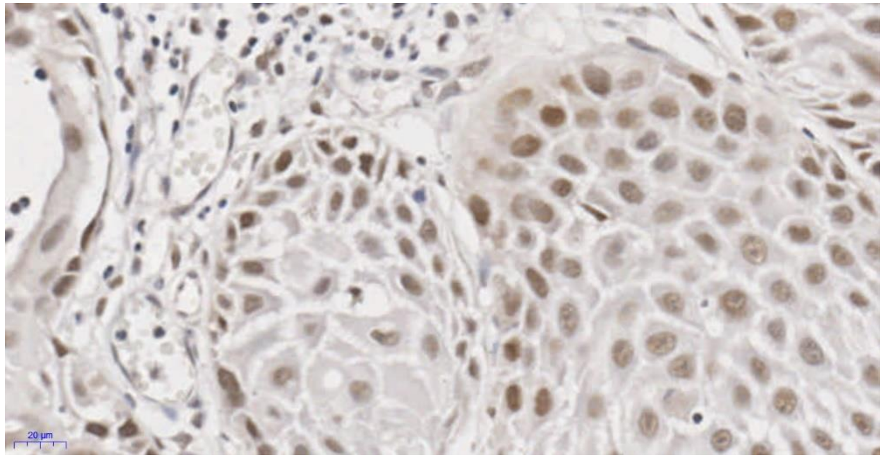
20 µm
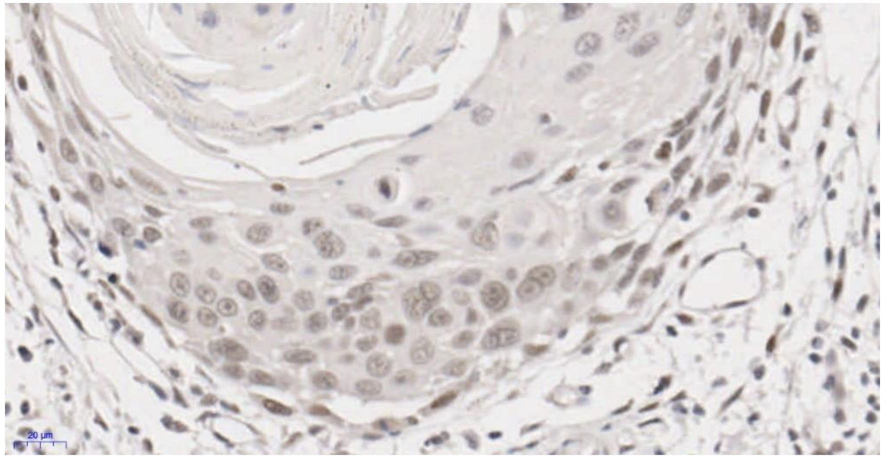
20 µm
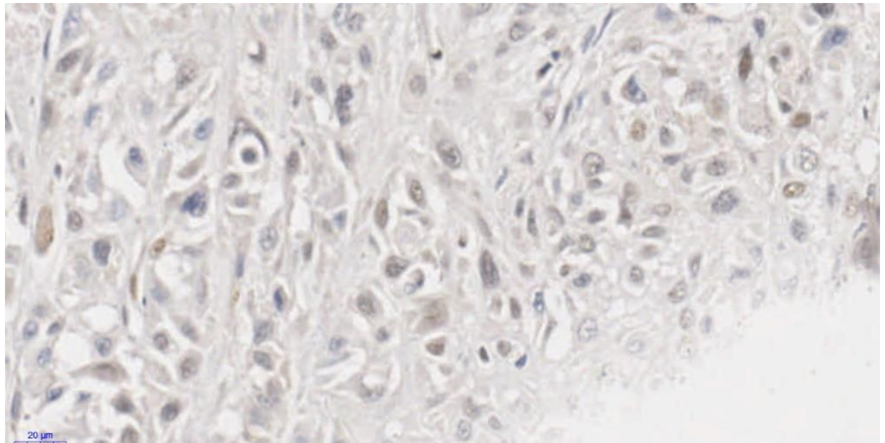
20 µm
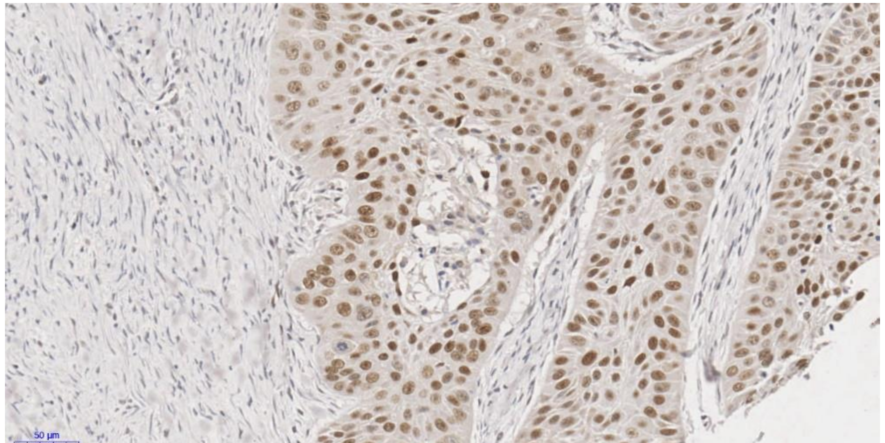
50 µm

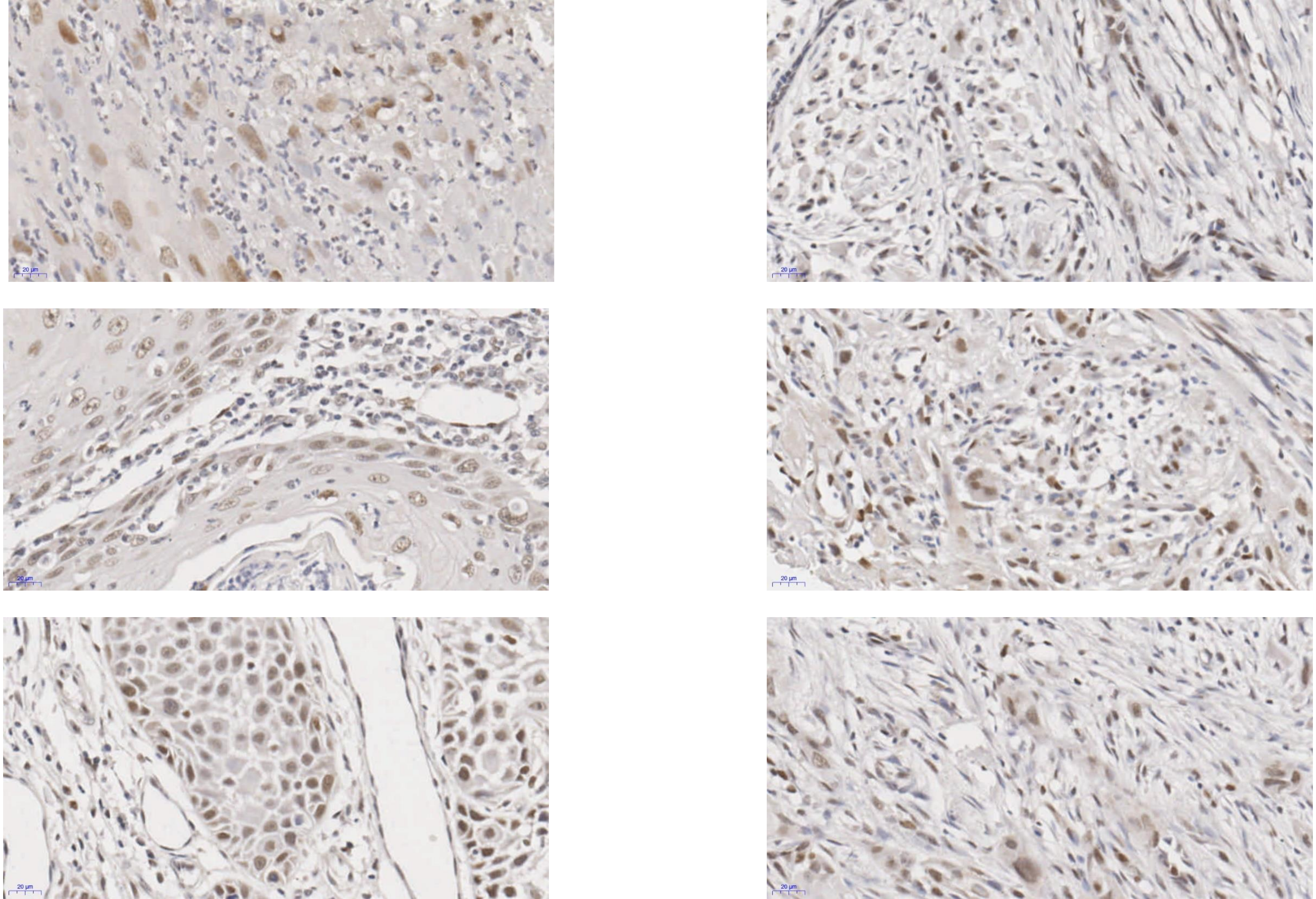
20 μm
20 μm
20 μm
20 μm
20 μm
20 μm

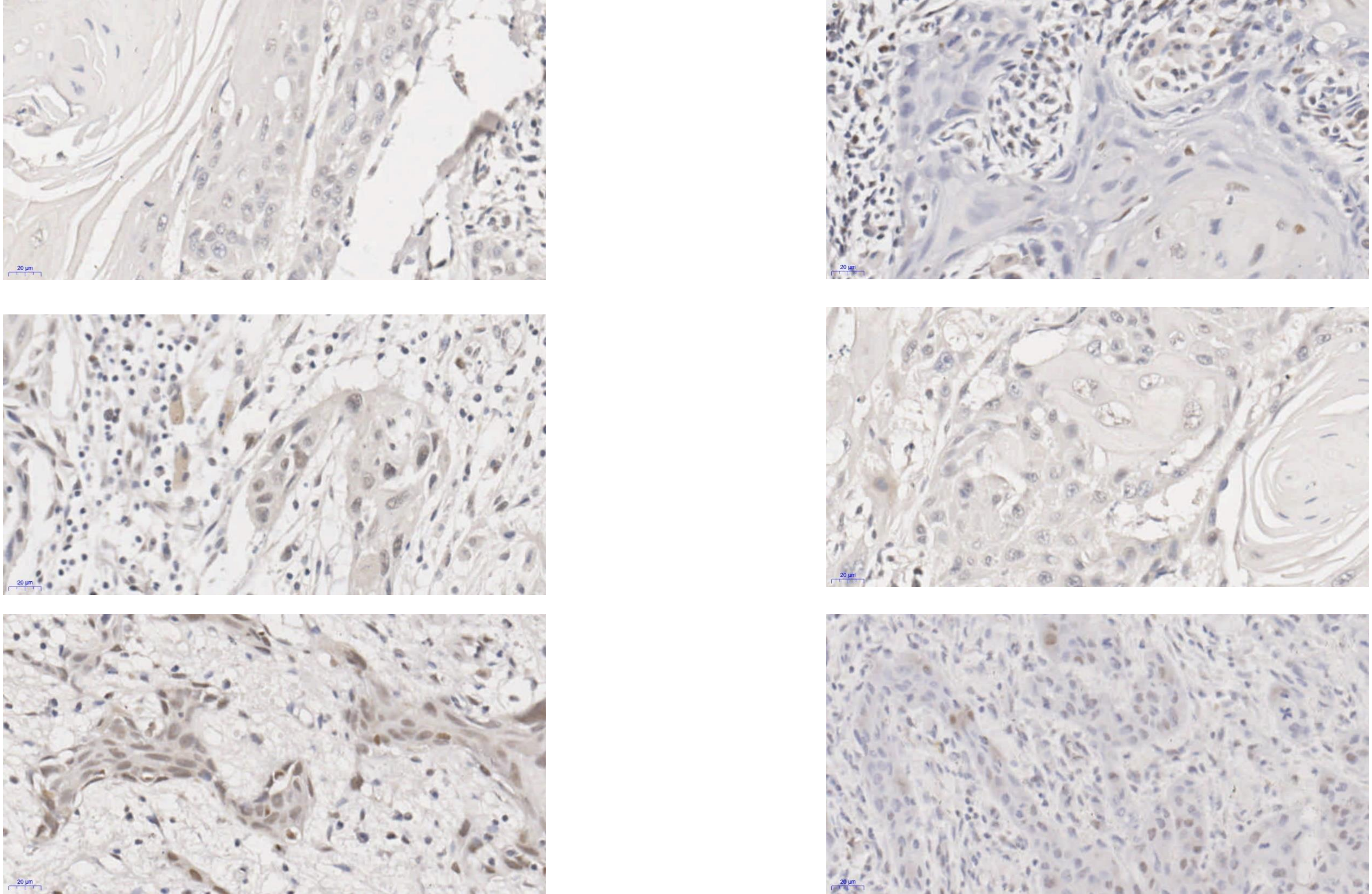
20 μm
20 μm
20 μm
20 μm
20 μm
20 μm

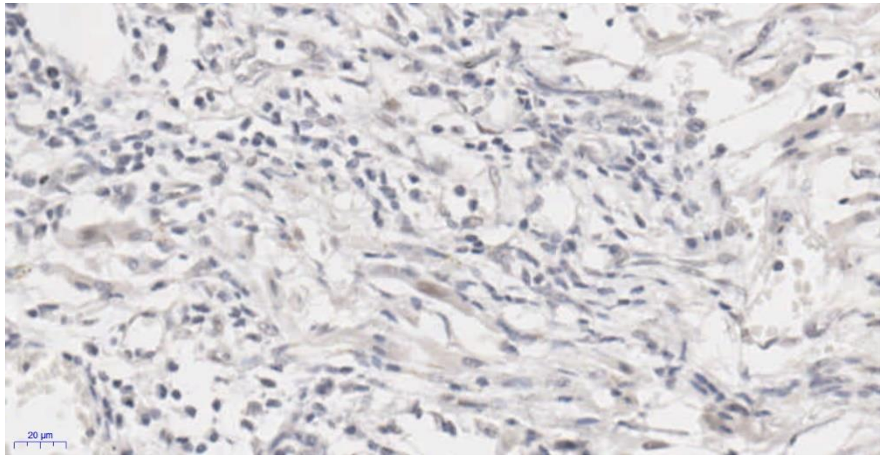
20 μm
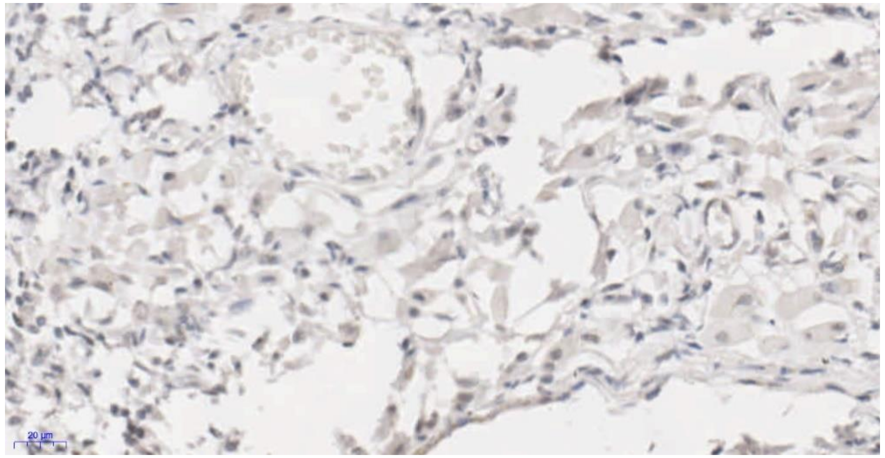
20 μm
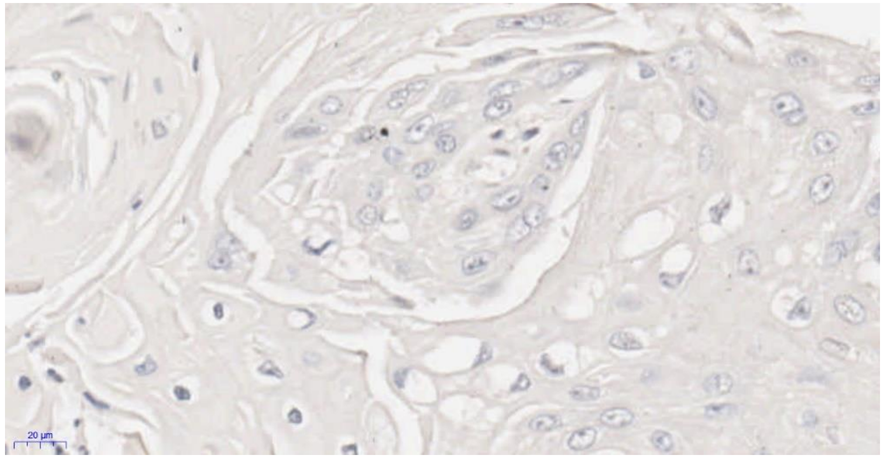
20 μm
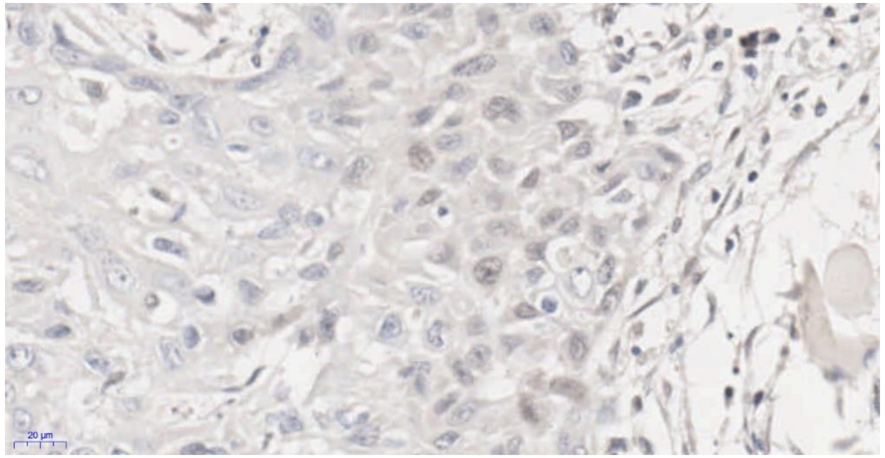
20 μm
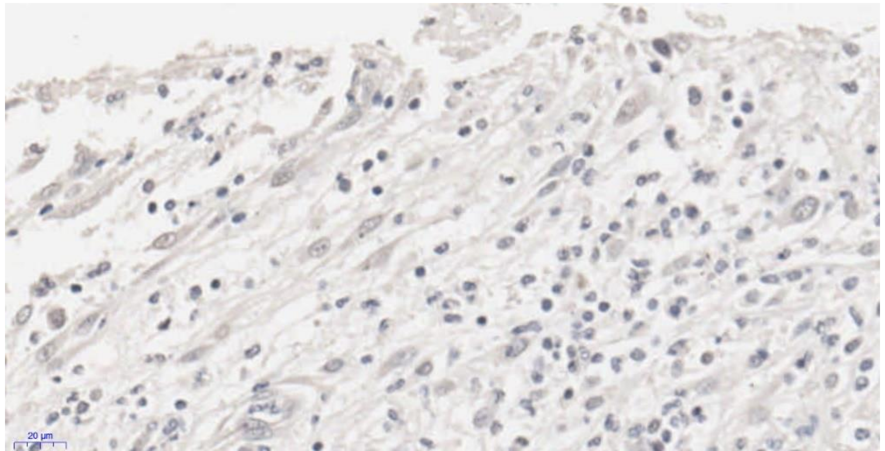
20 μm
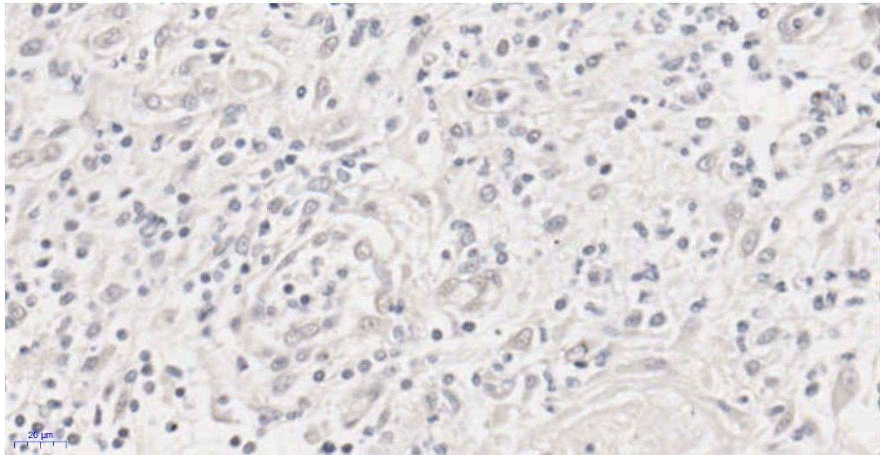
20 μm

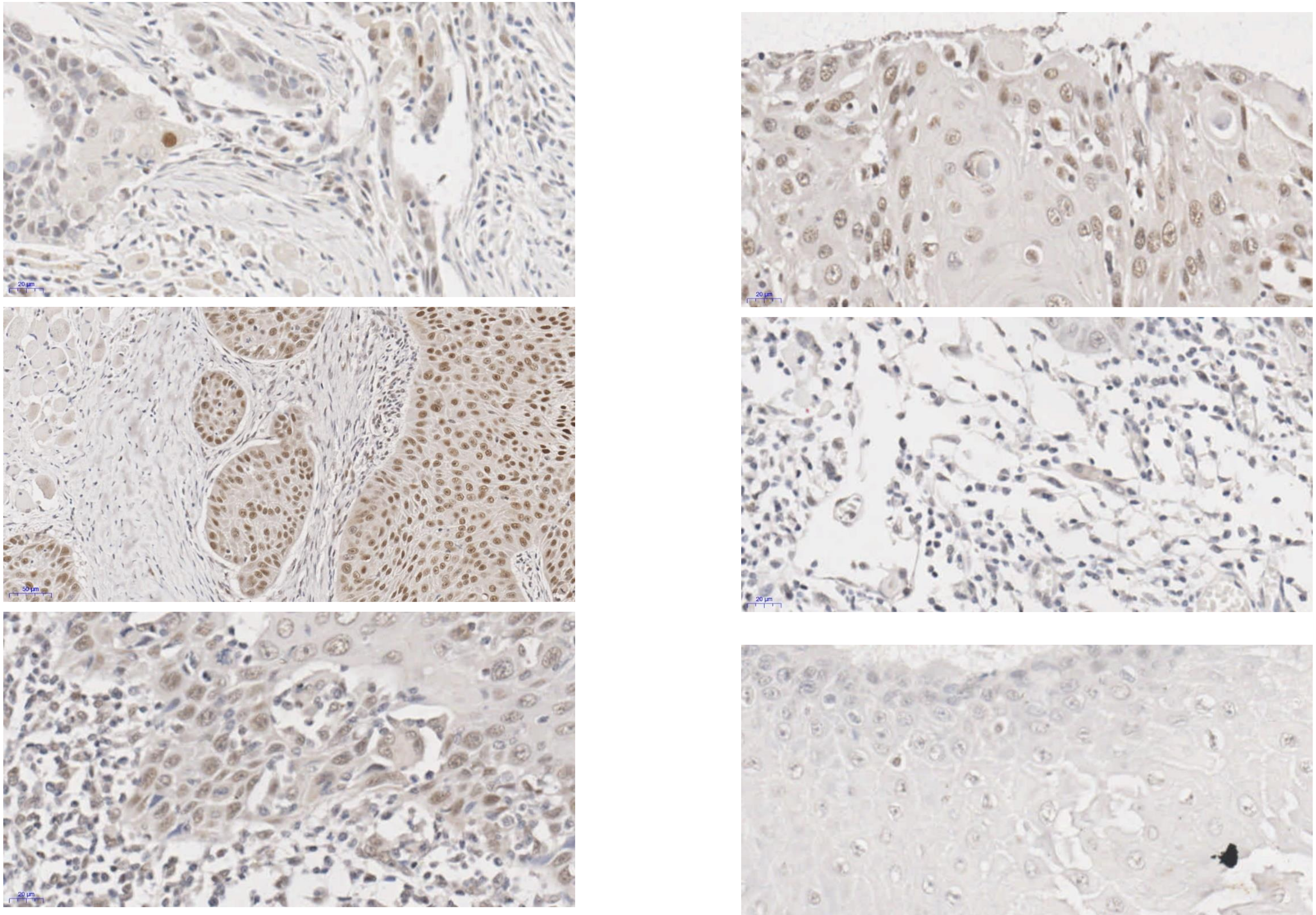
20 μm
20 μm
50 μm
20 μm
20 μm

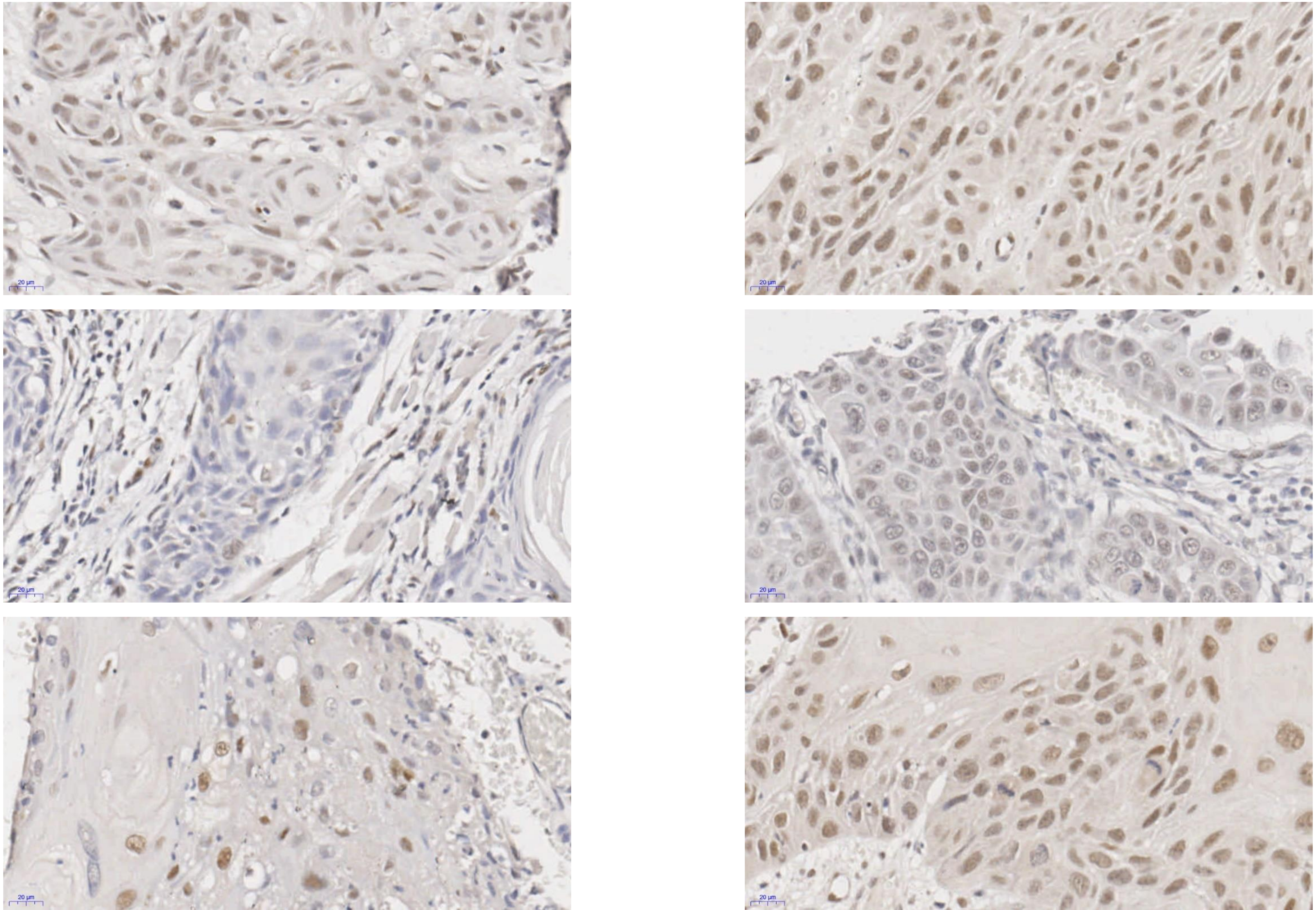
20 µm
20 µm
20 µm
20 µm
20 µm
20 µm

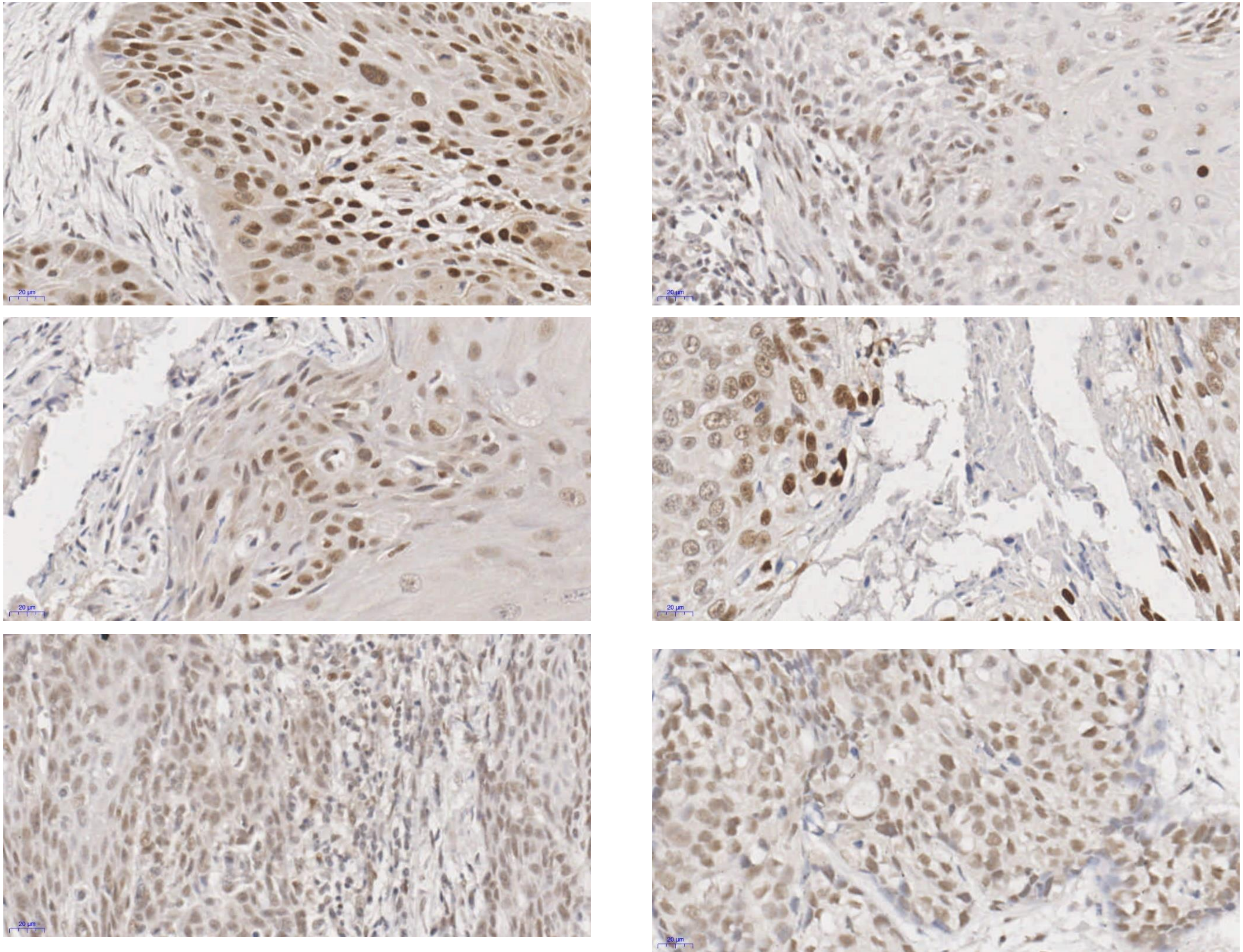
20 μm
20 μm
20 μm
20 μm
20 μm
20 μm

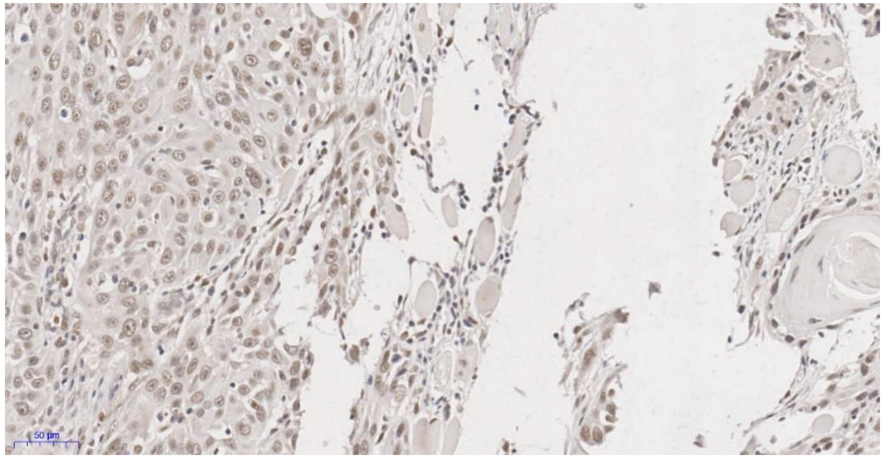
50 μm

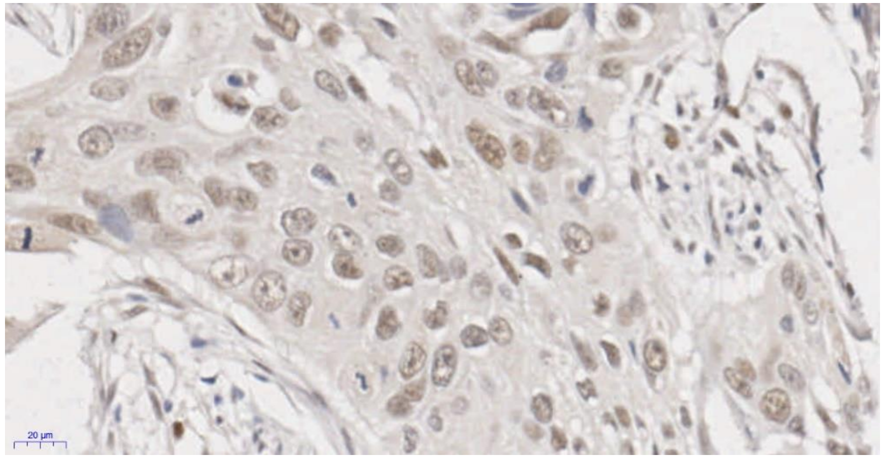
20 μm

SUV39H1
NM

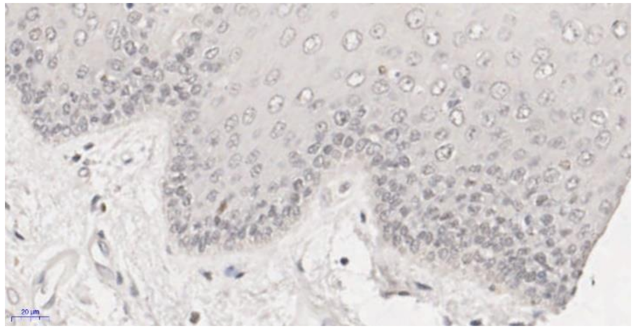
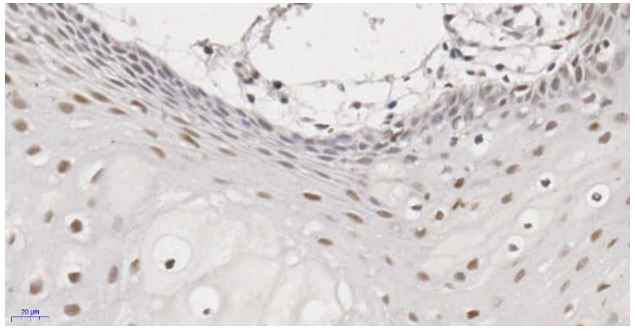
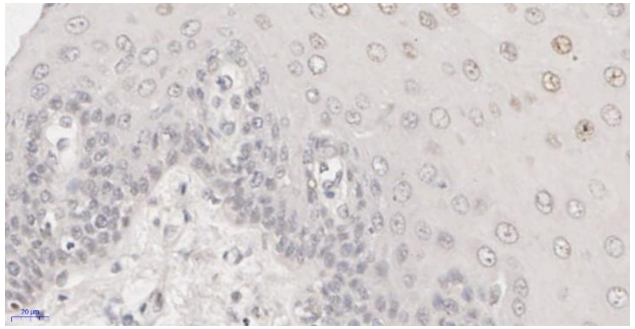
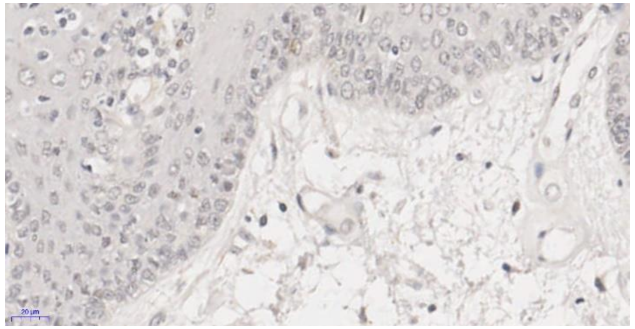
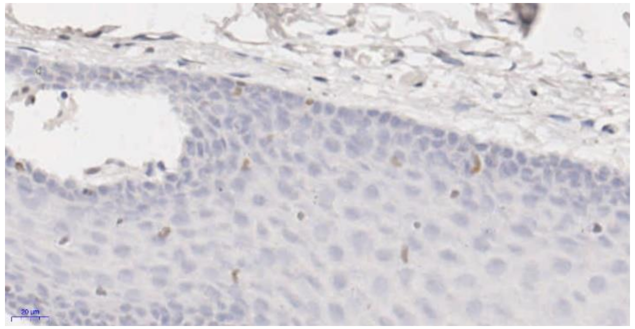
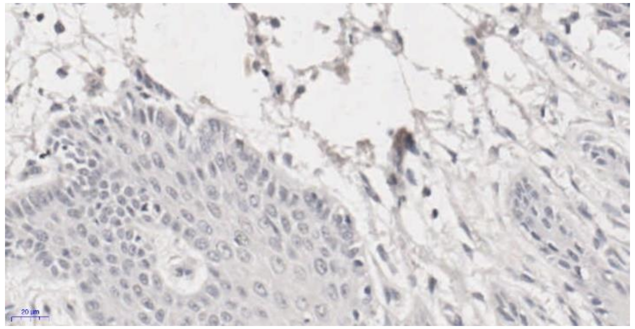
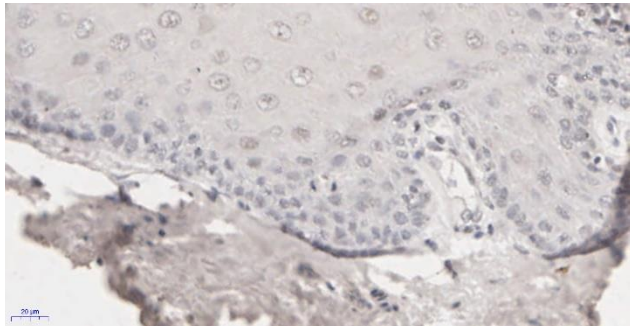
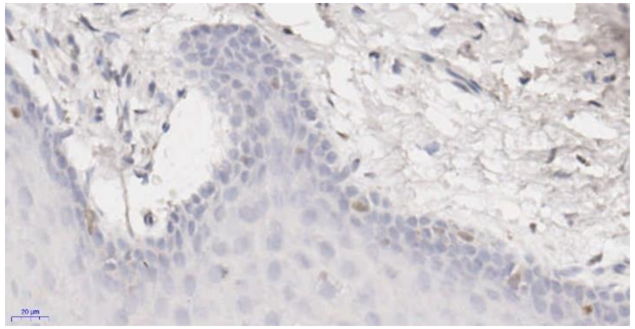

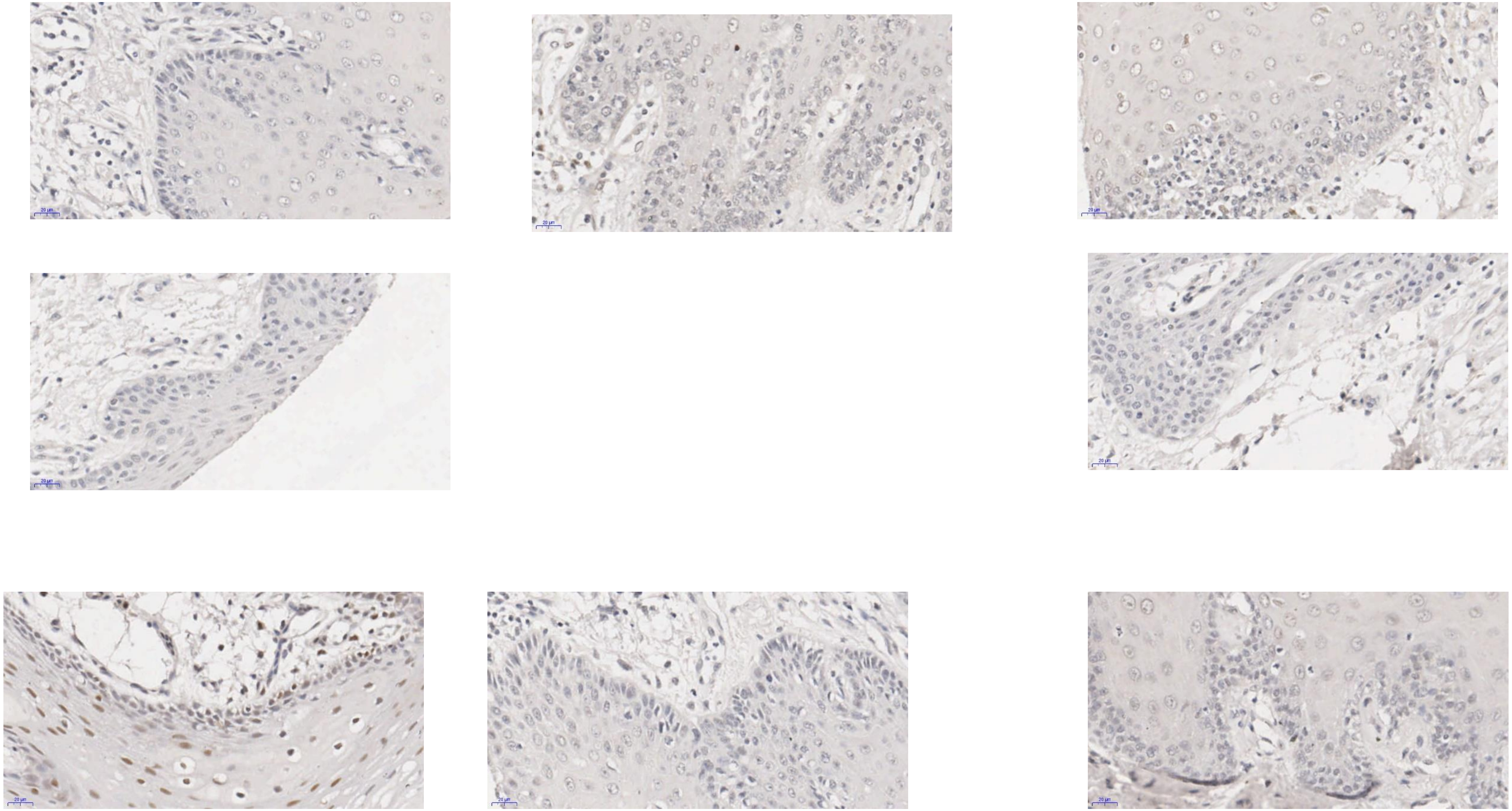

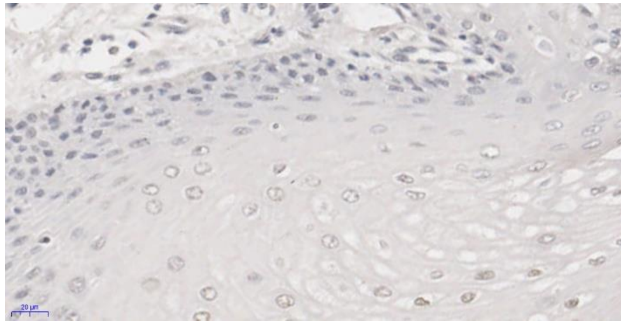
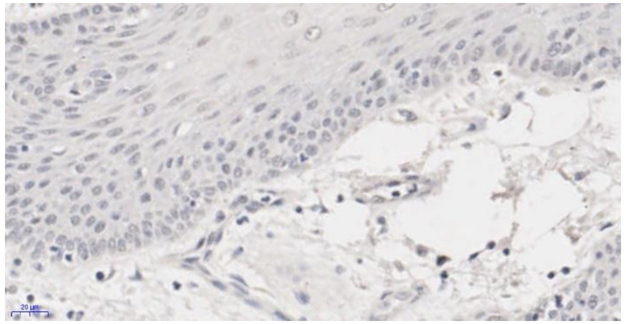
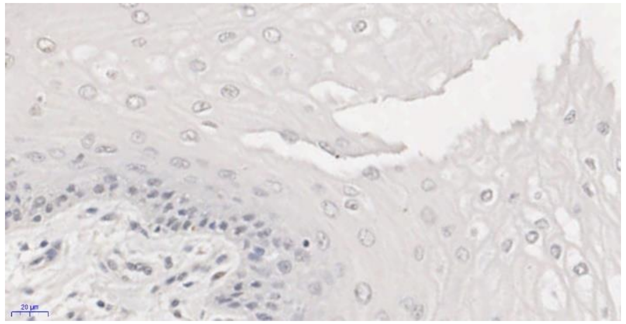
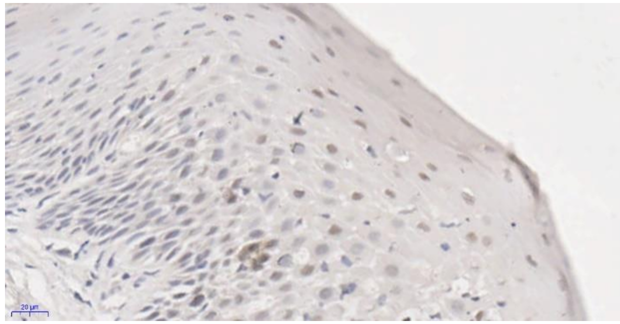
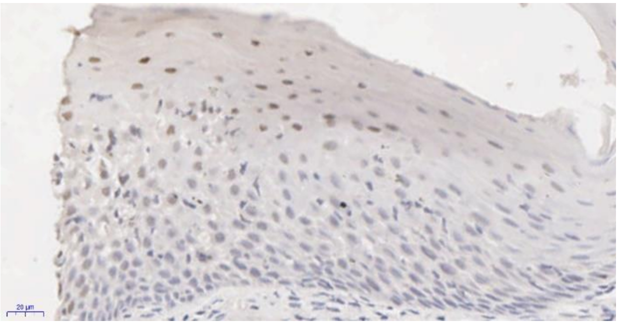
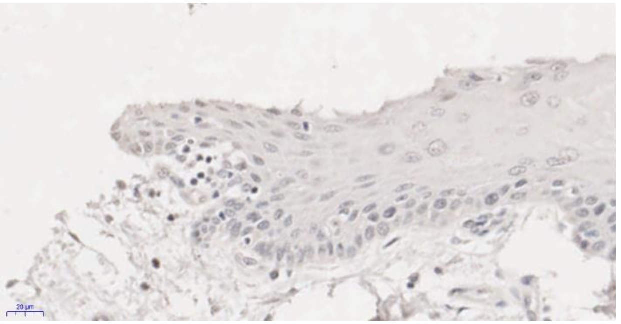
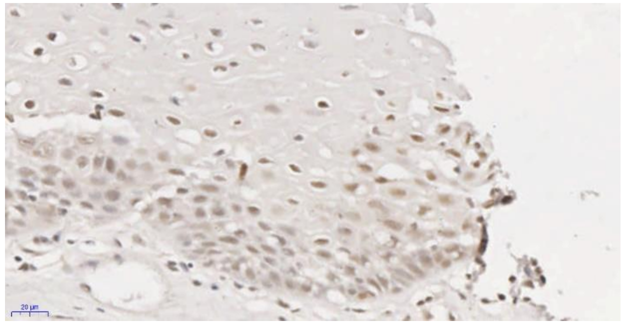
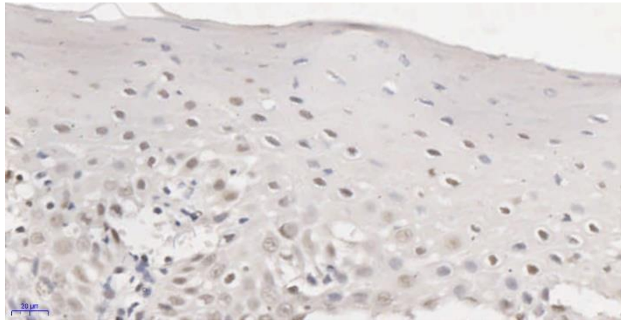

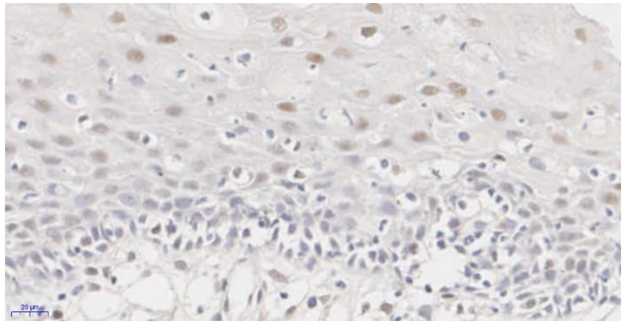
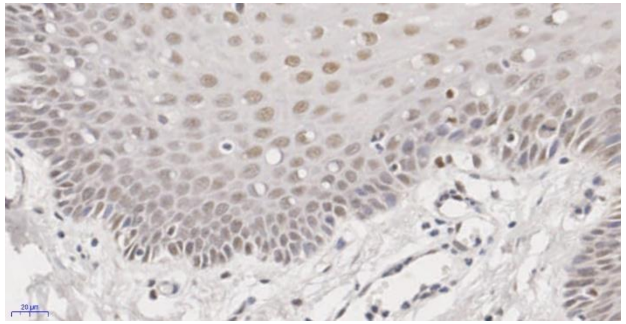
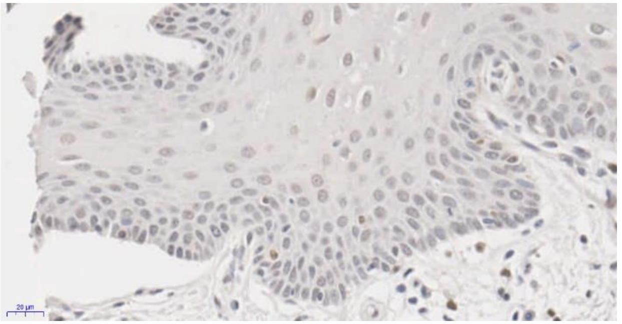
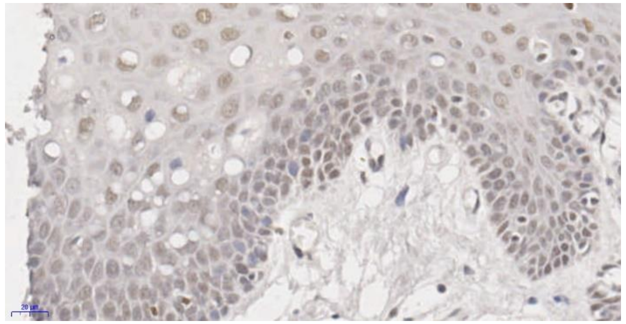
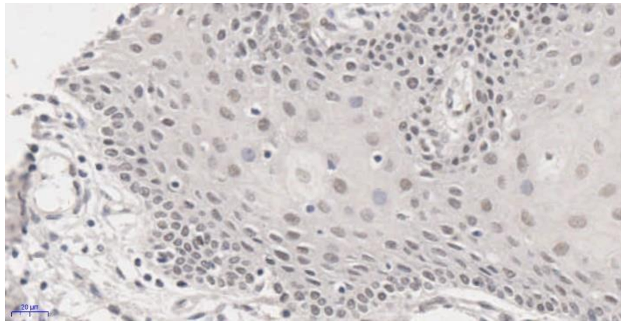
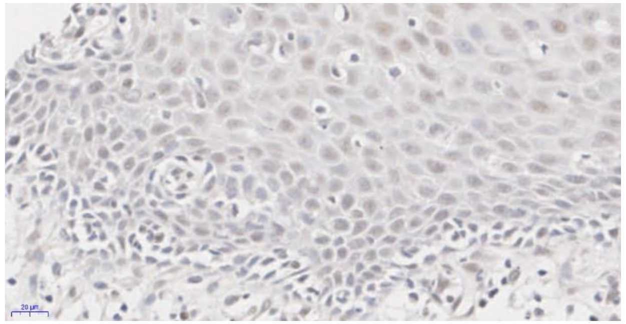
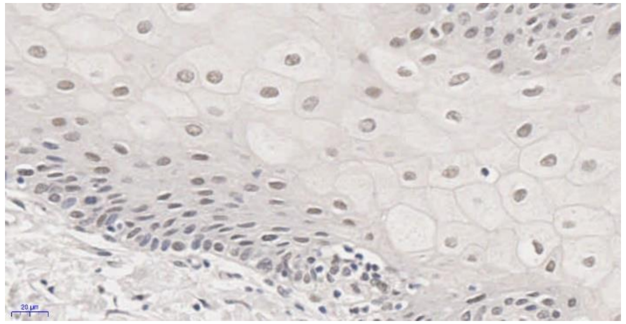
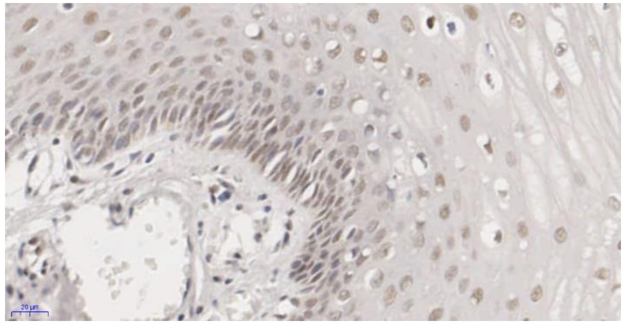

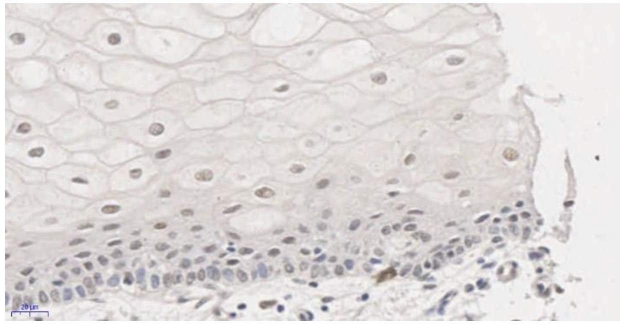
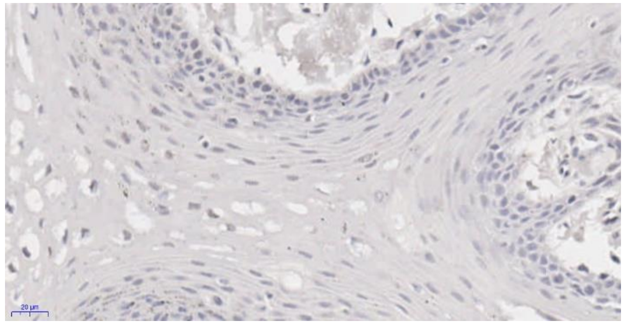
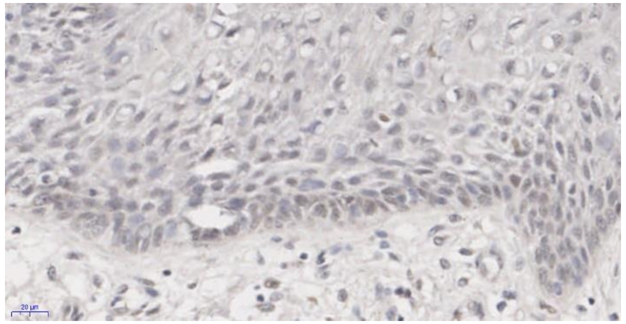
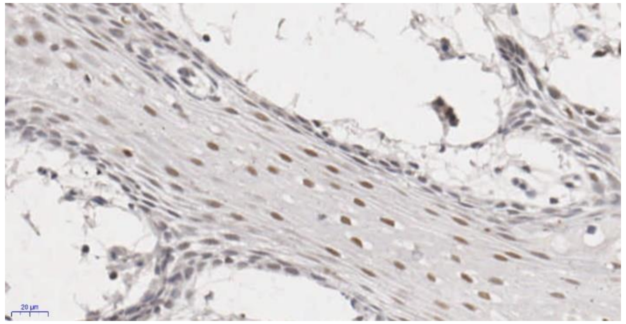
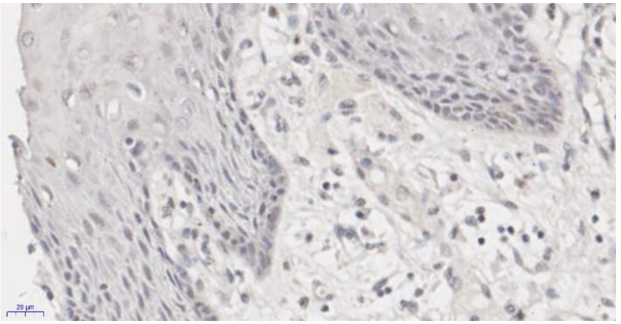
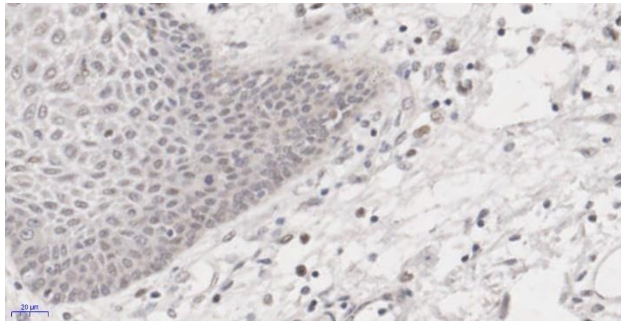
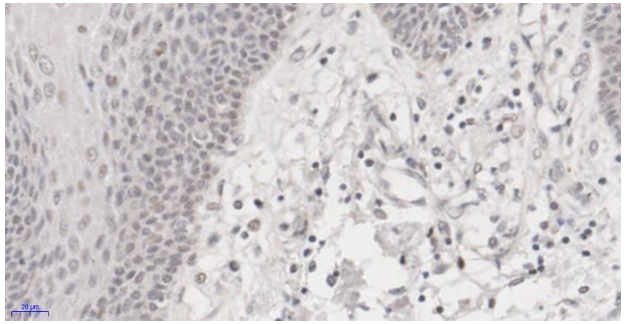
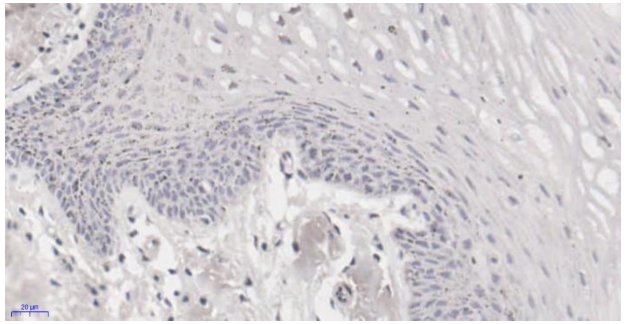

Notch1 OSCC

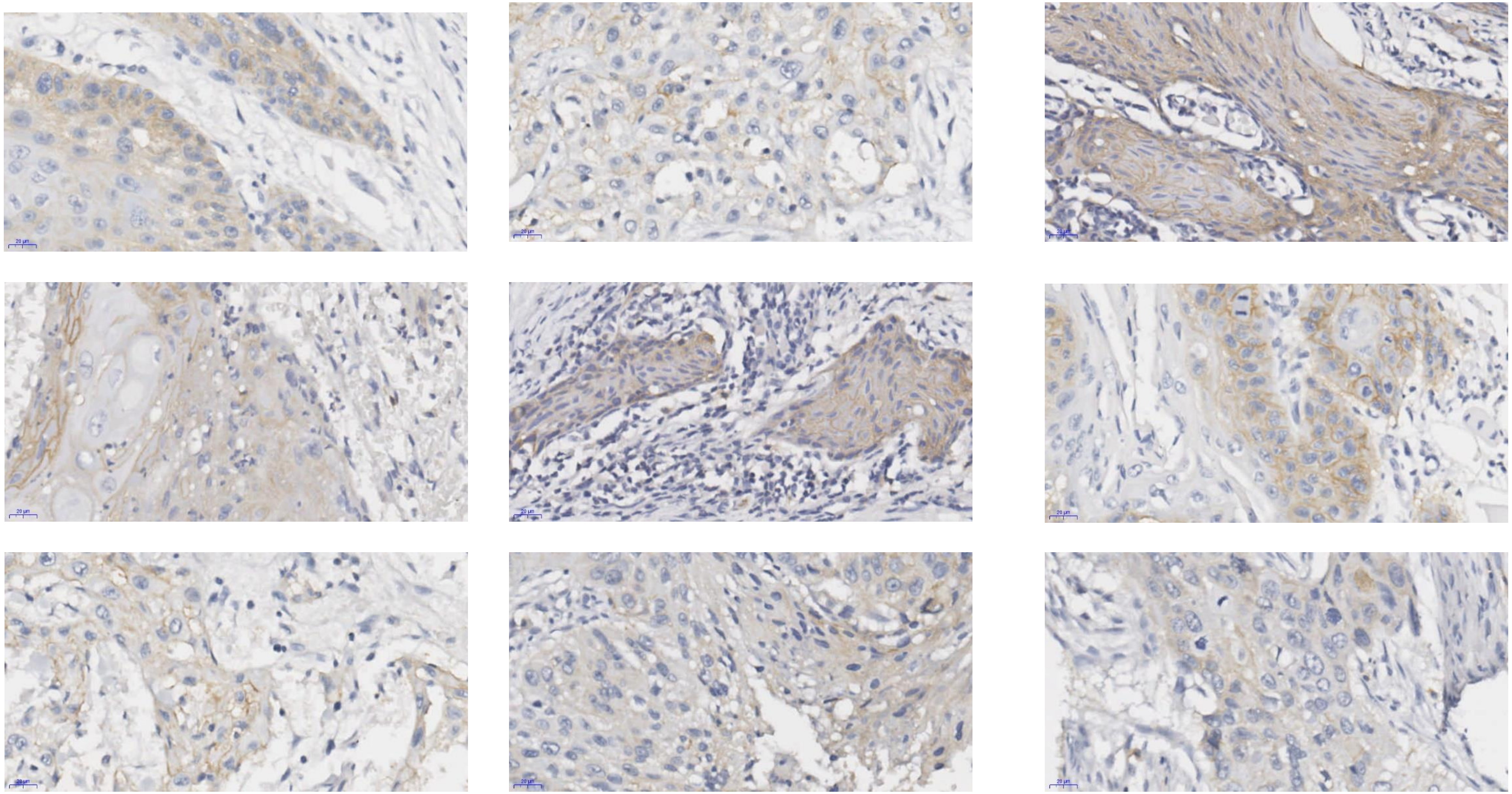

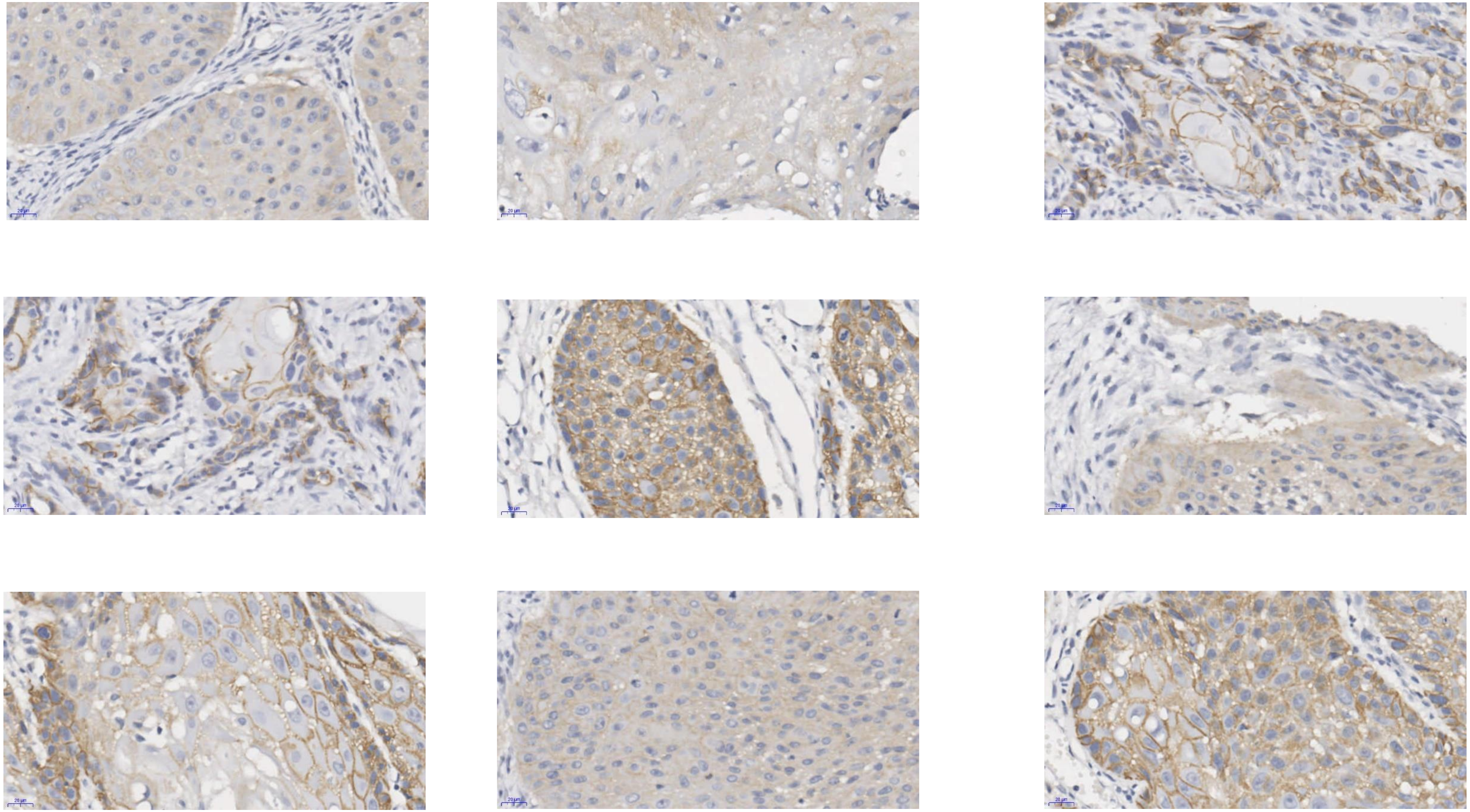

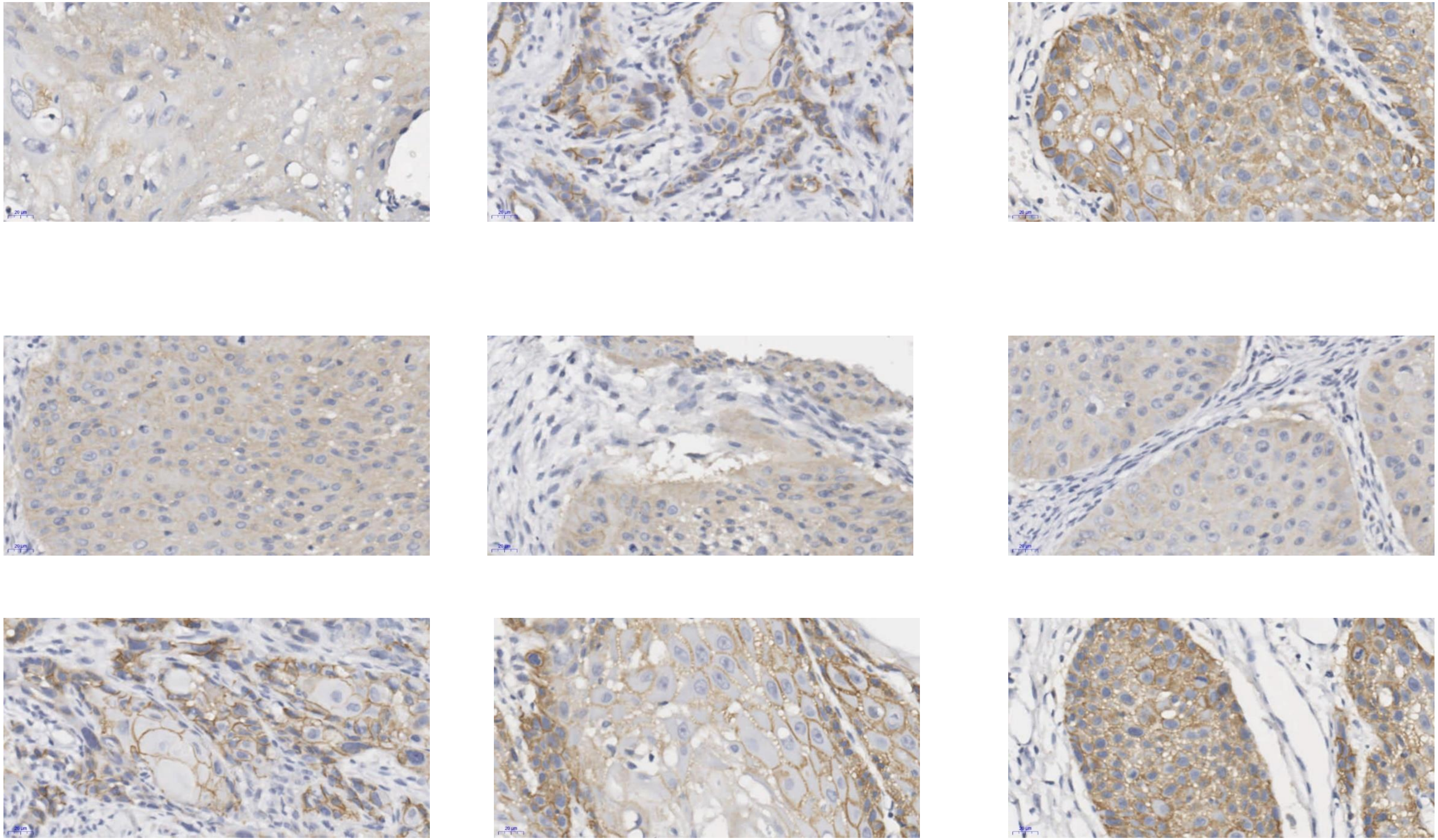

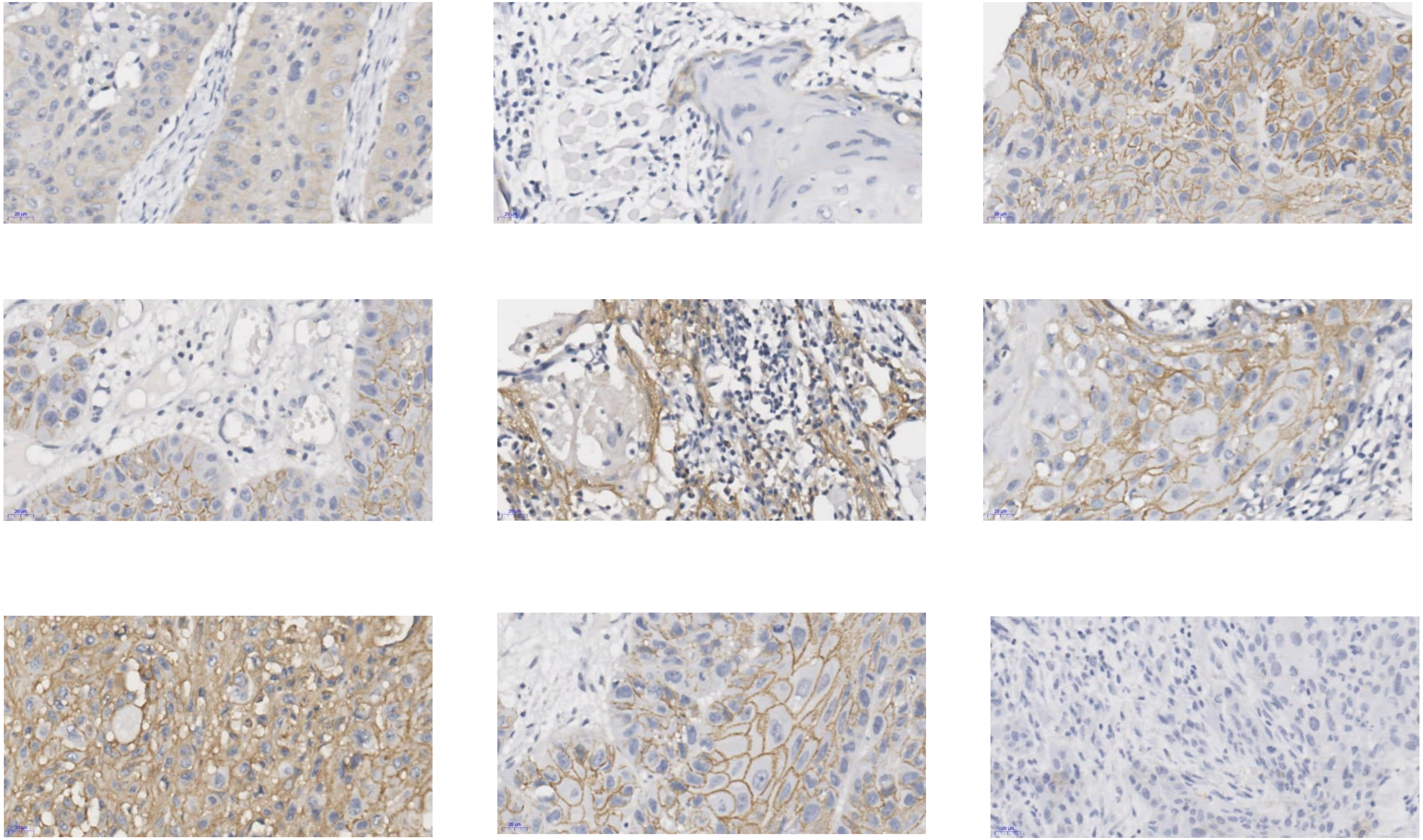

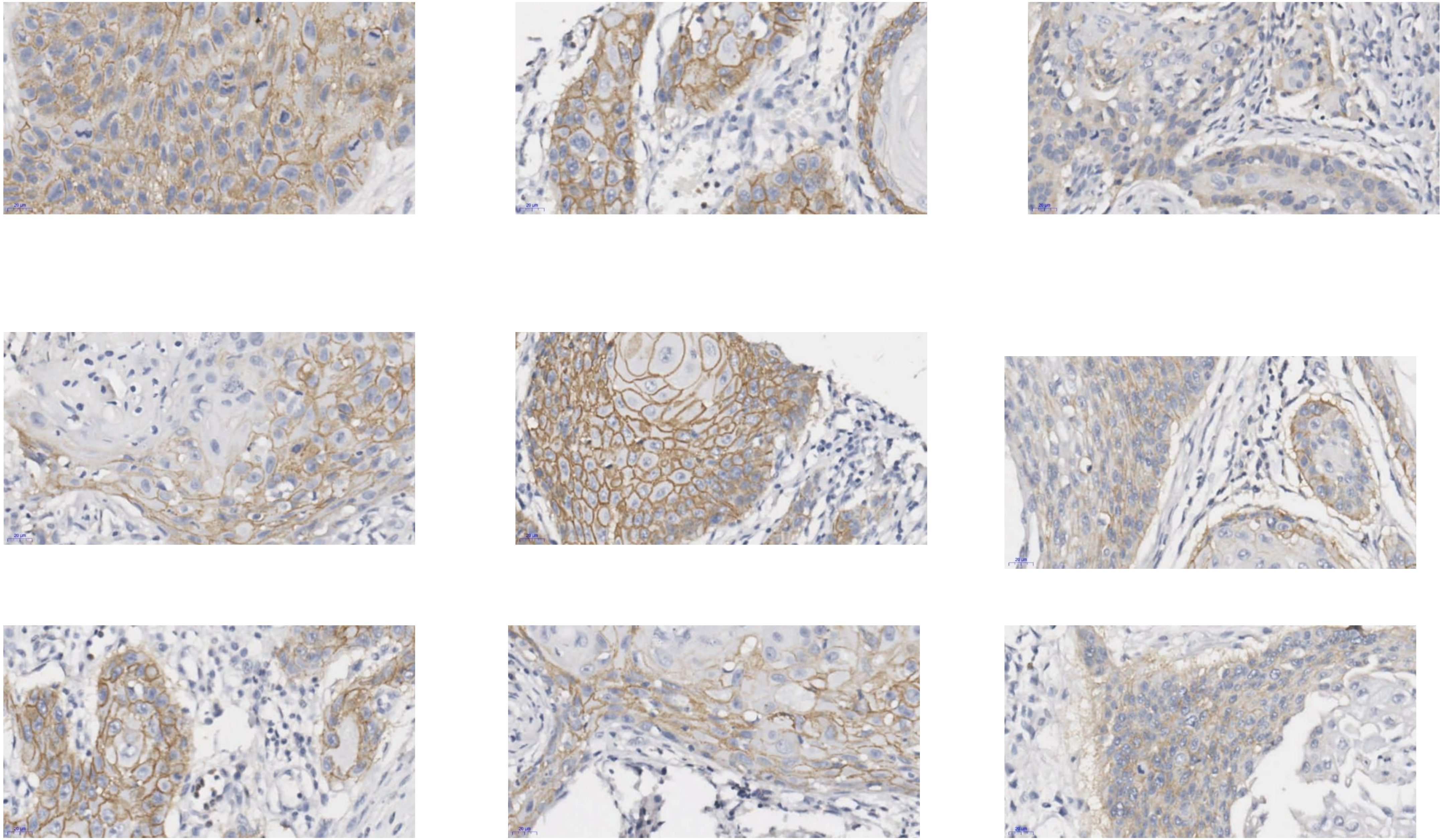

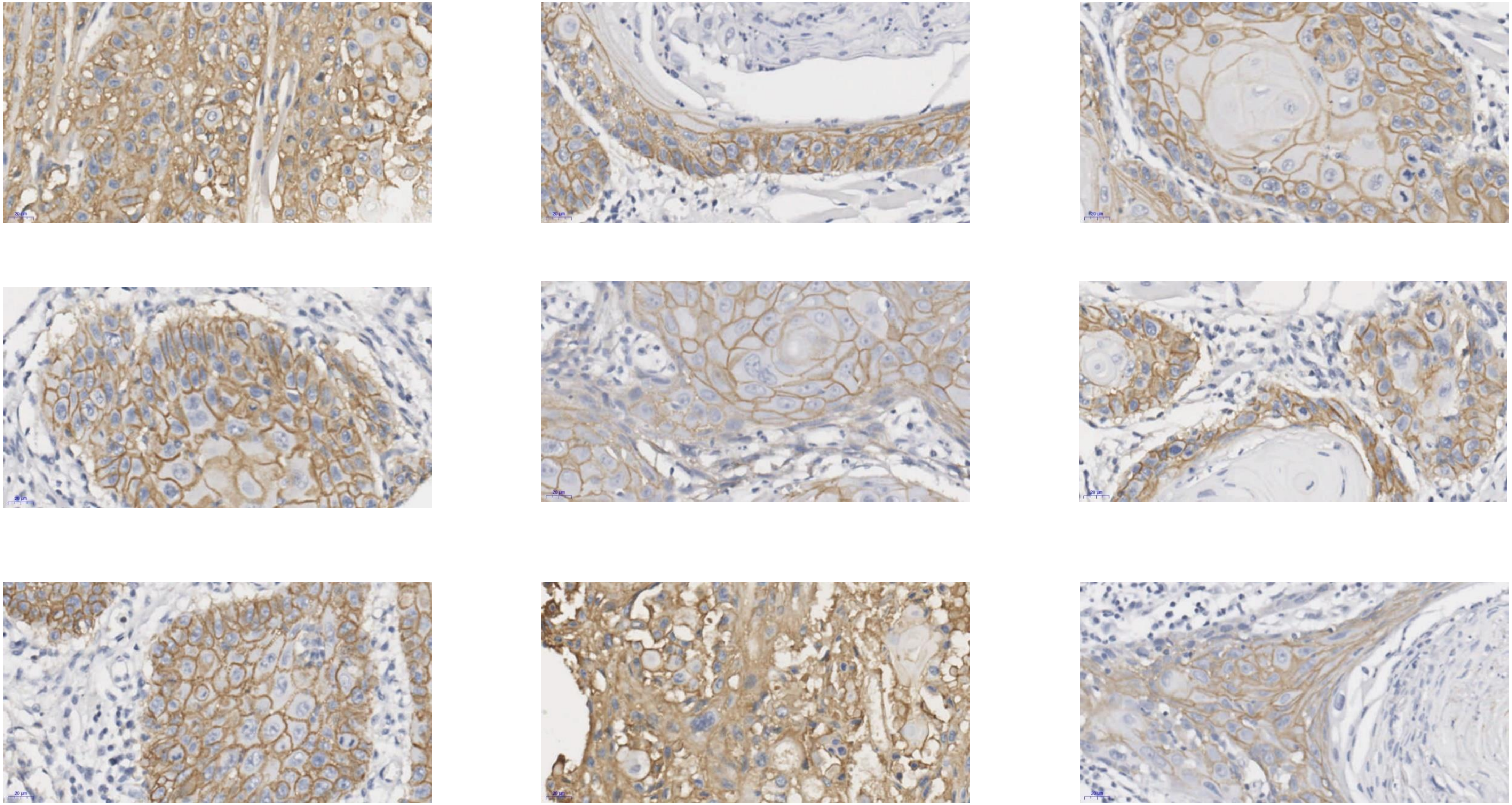

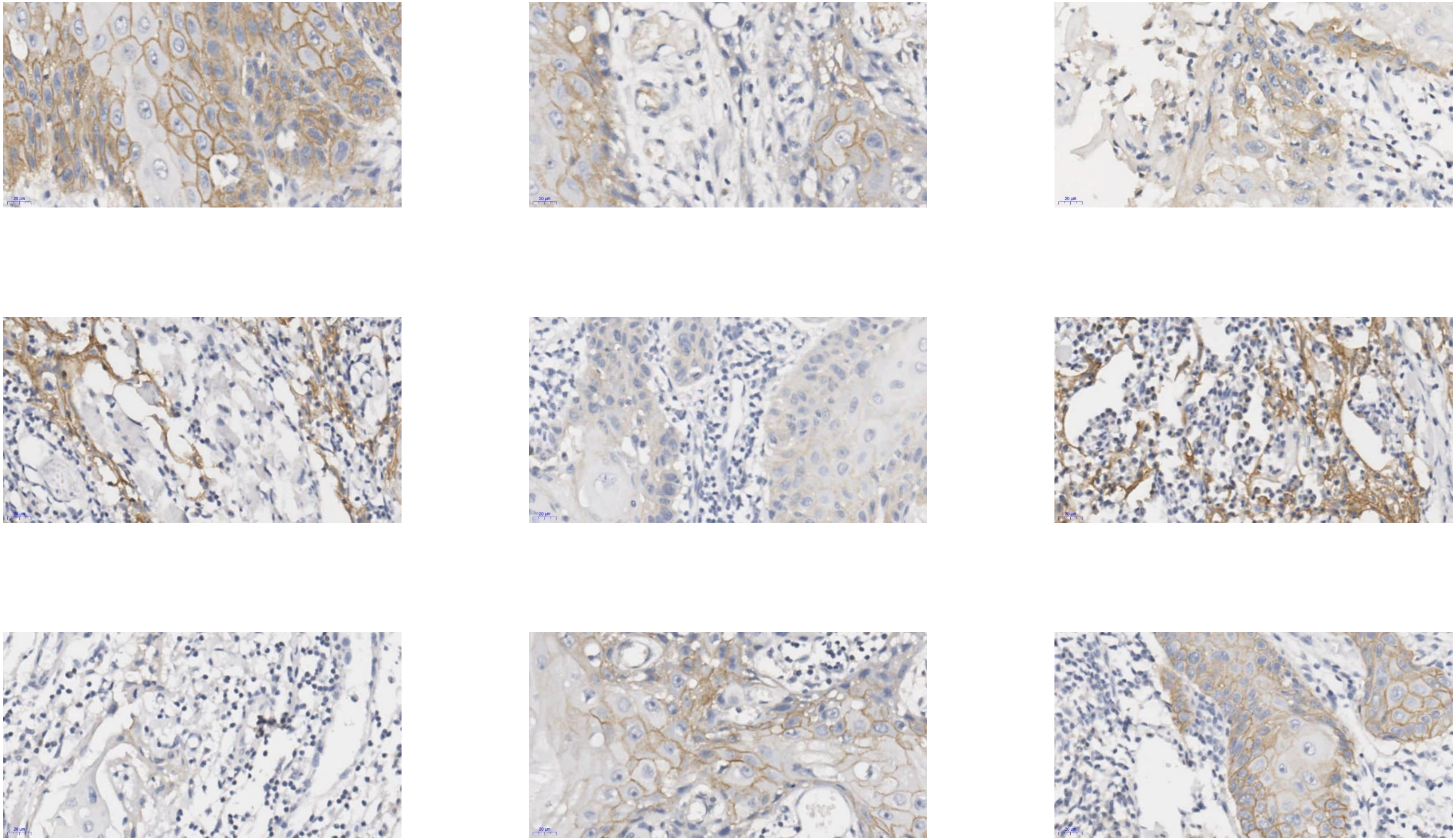

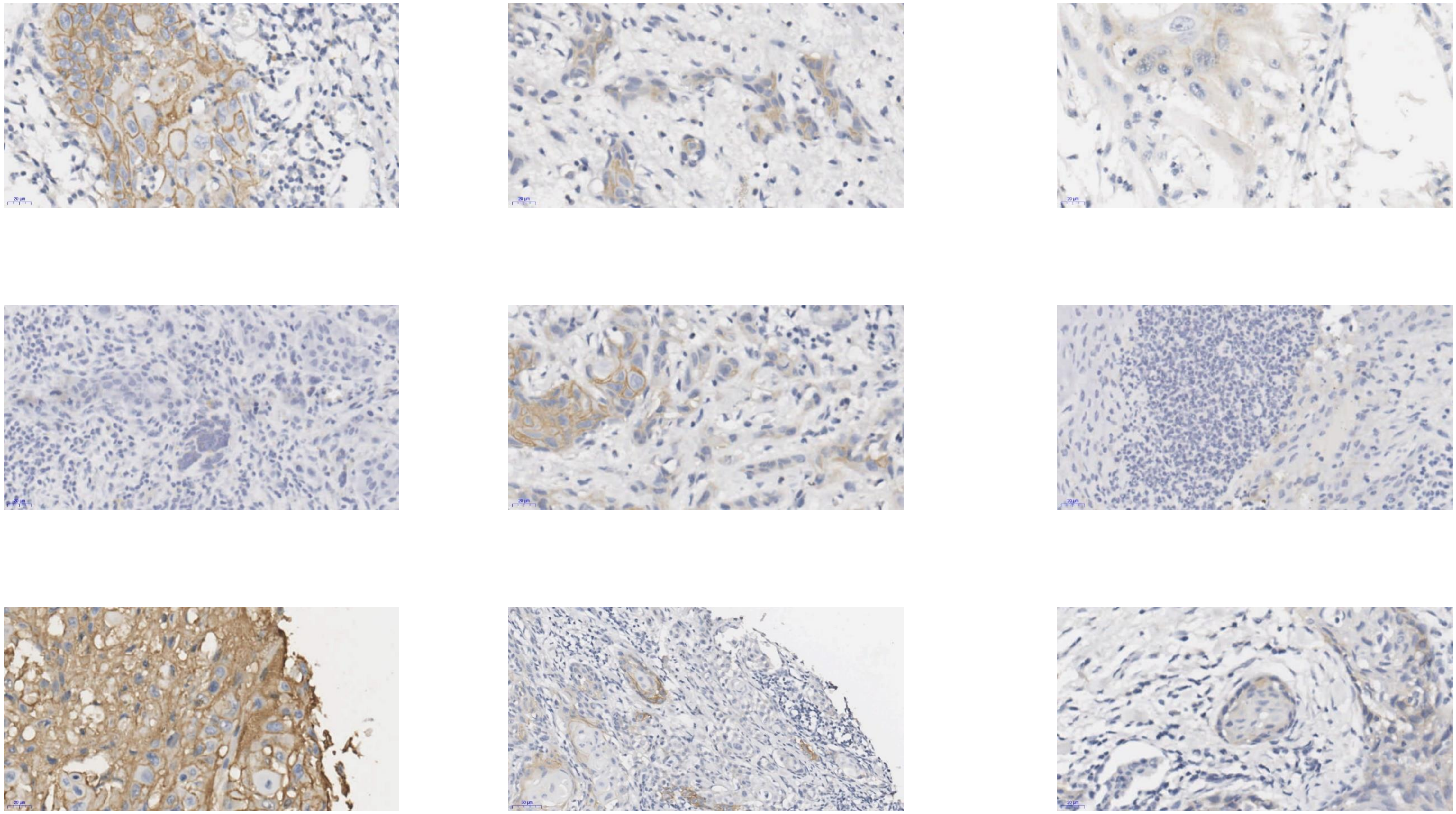

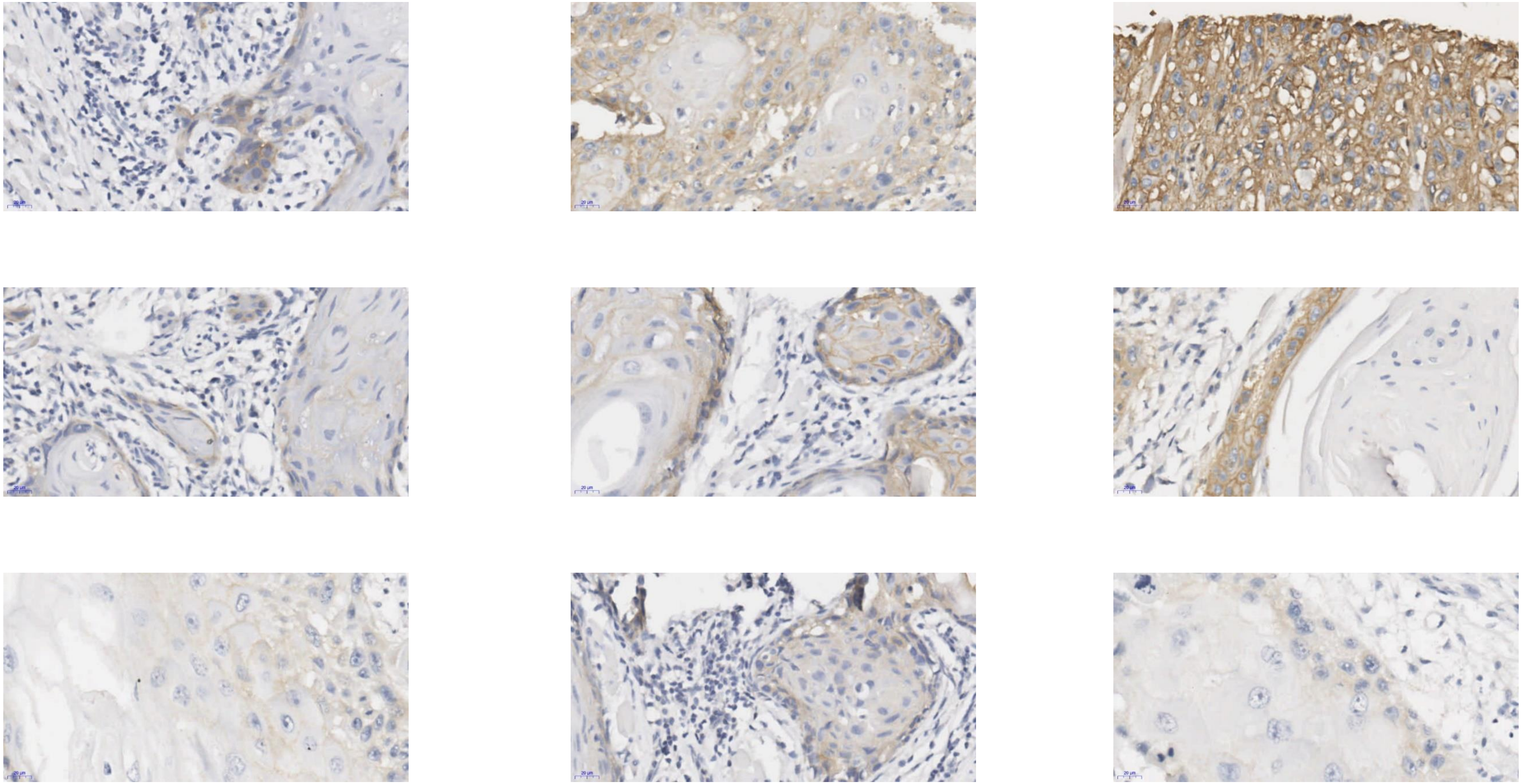

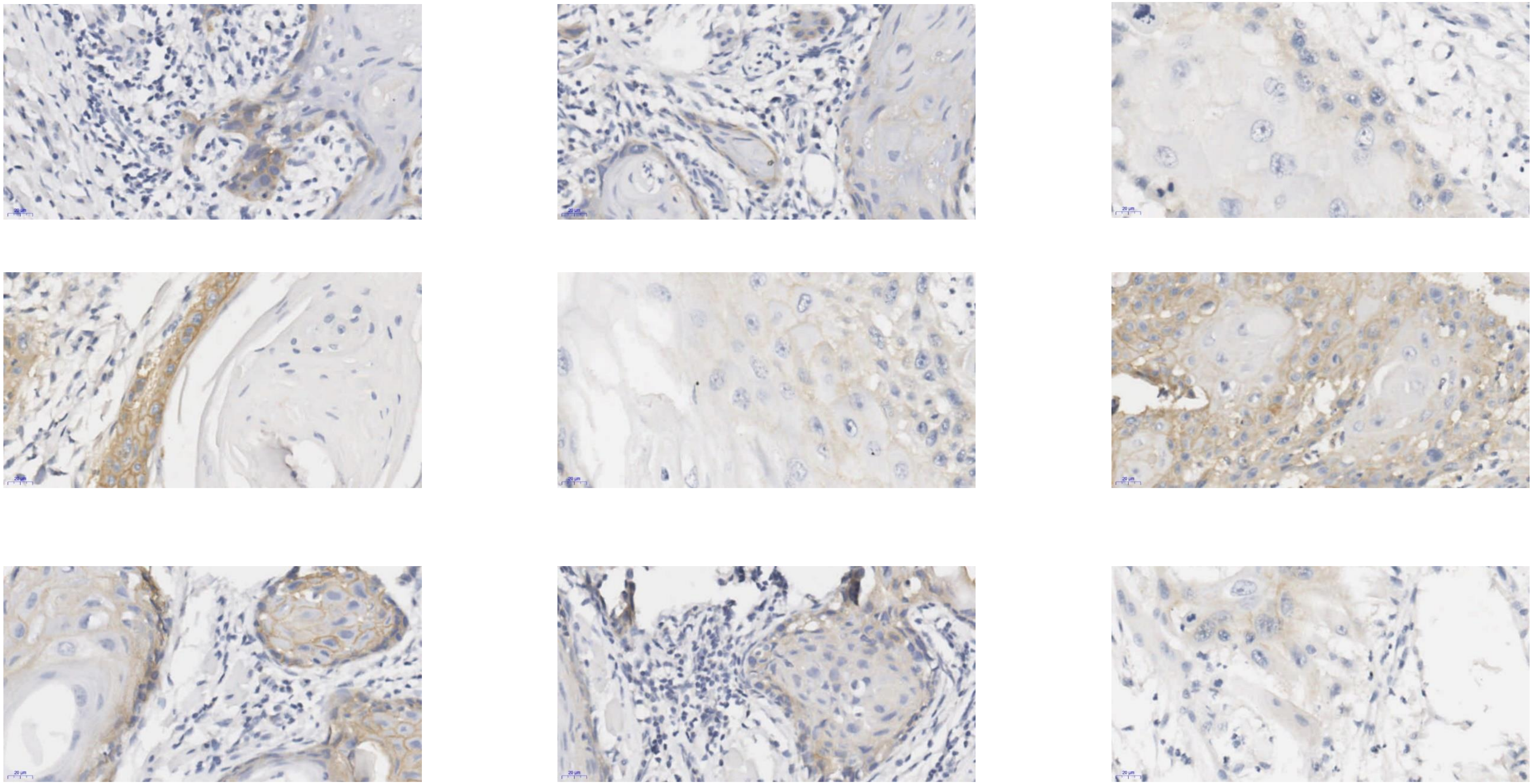

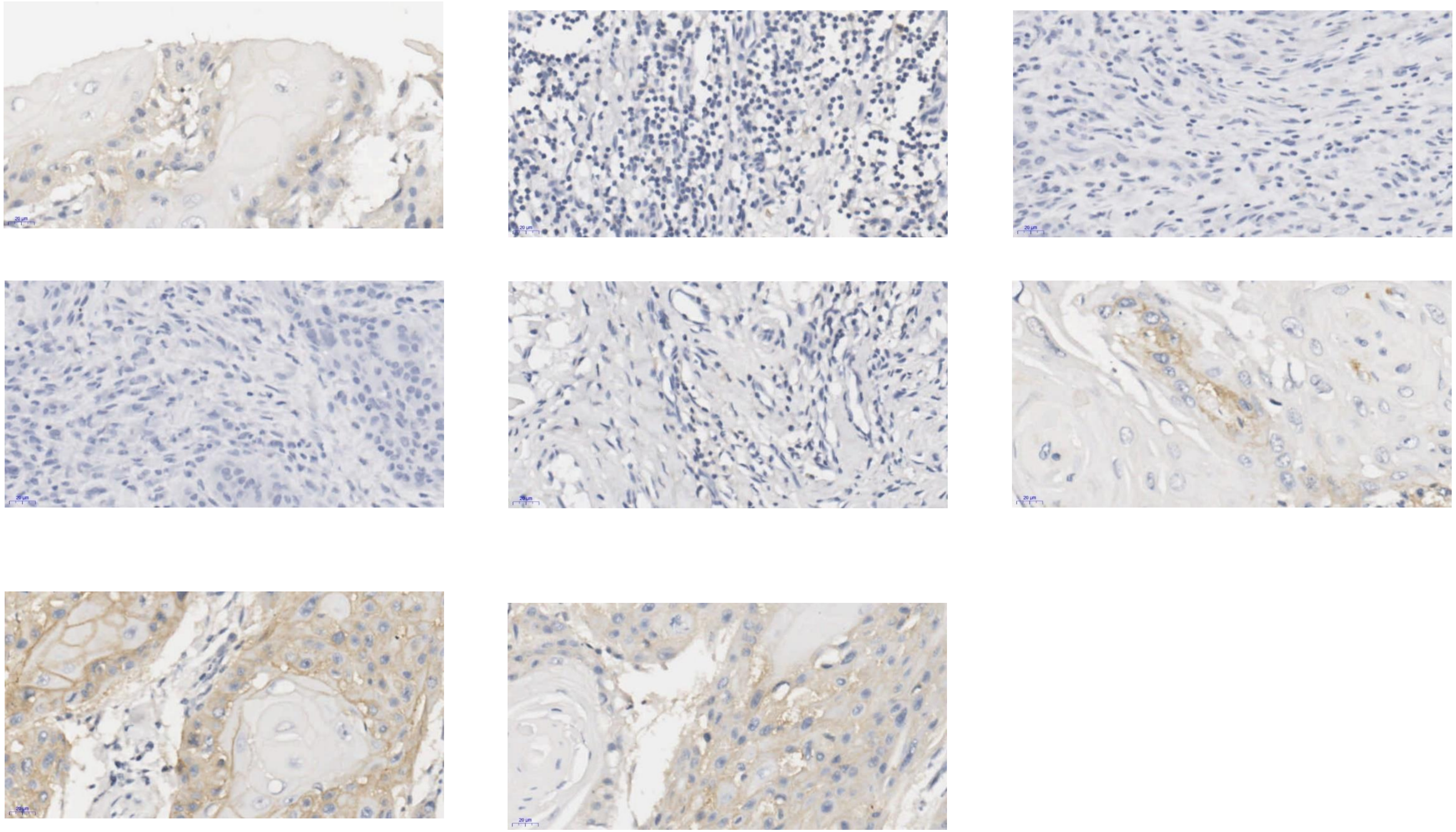

CD31 OSCC

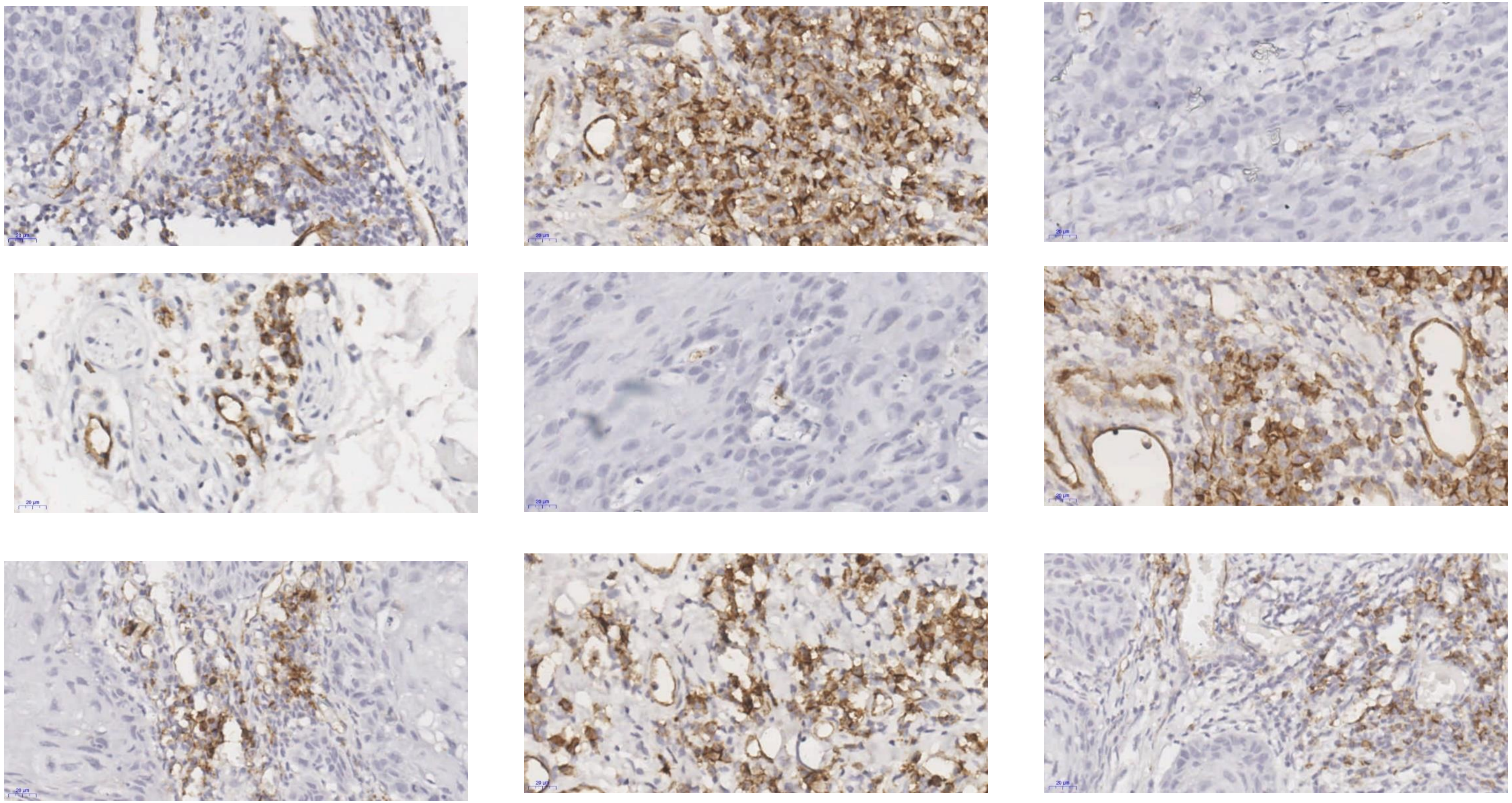

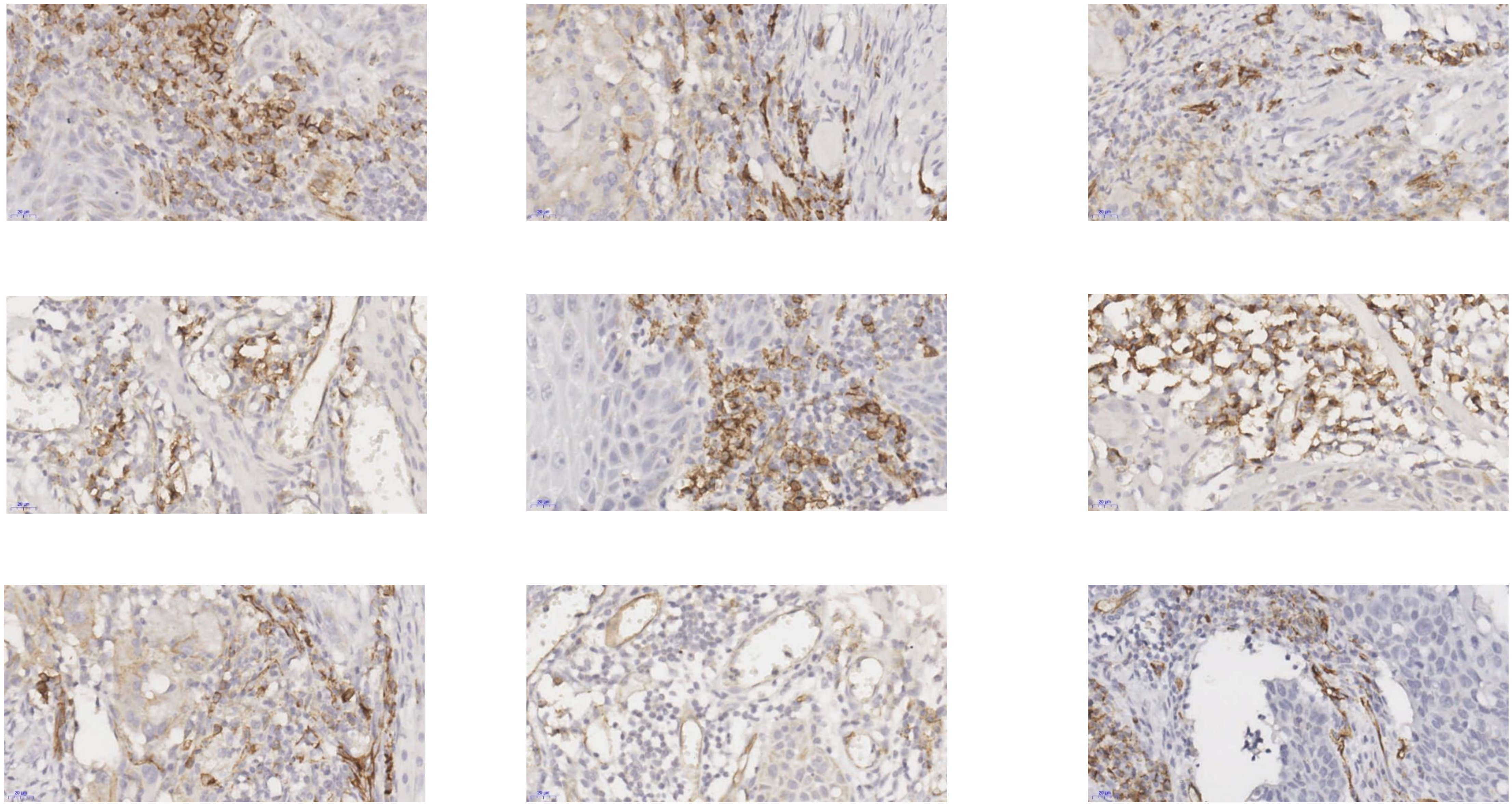

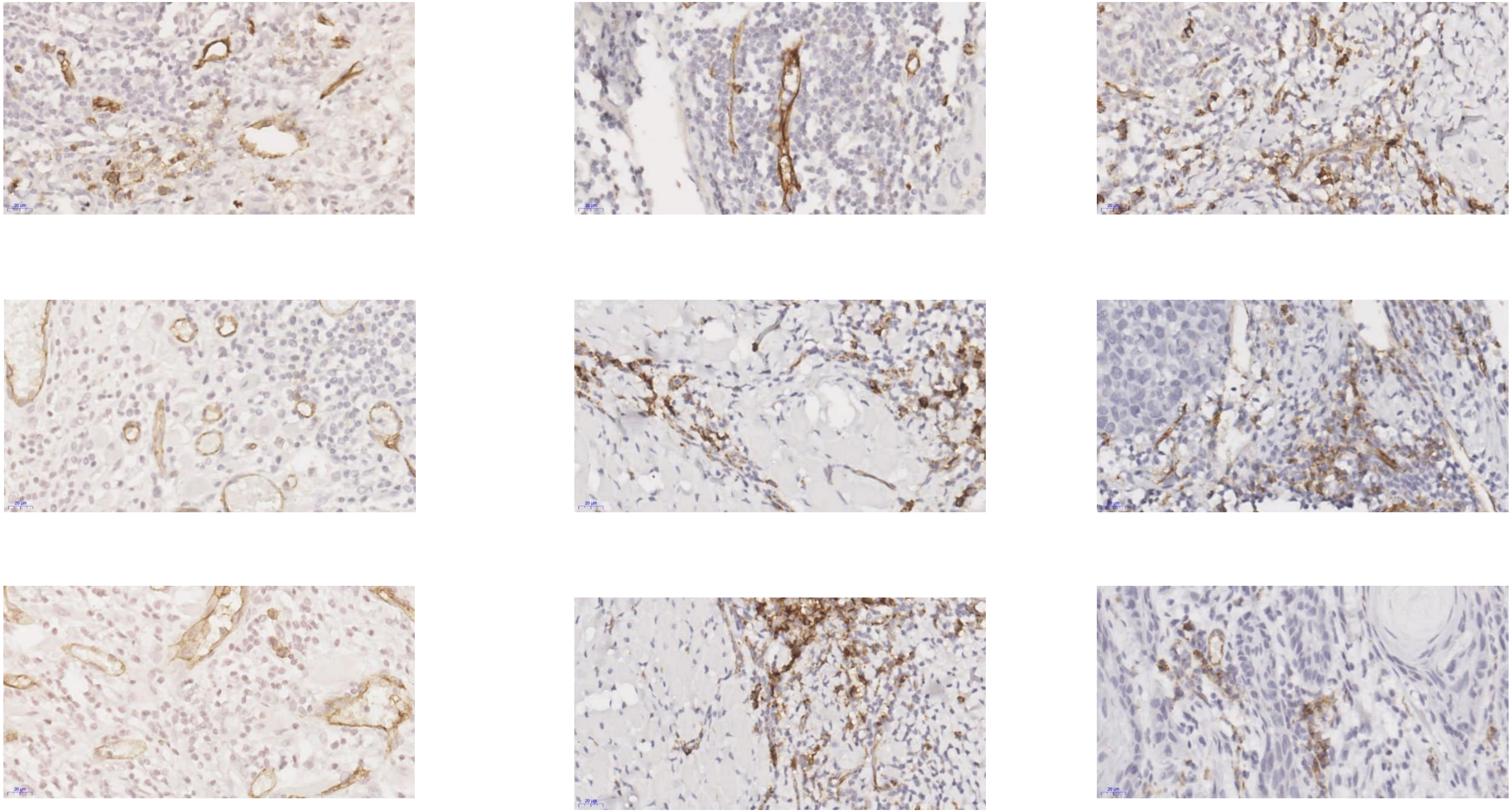

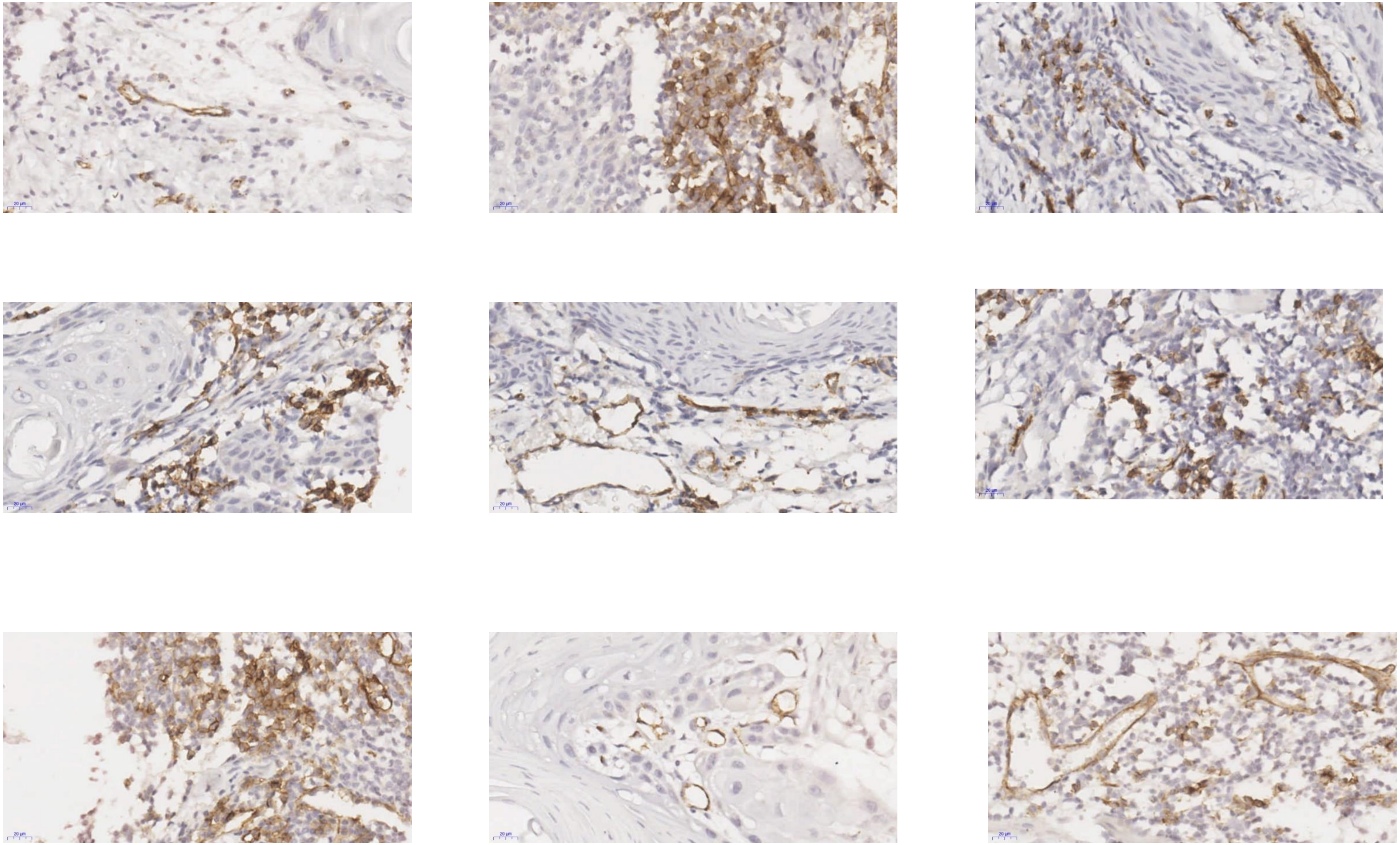

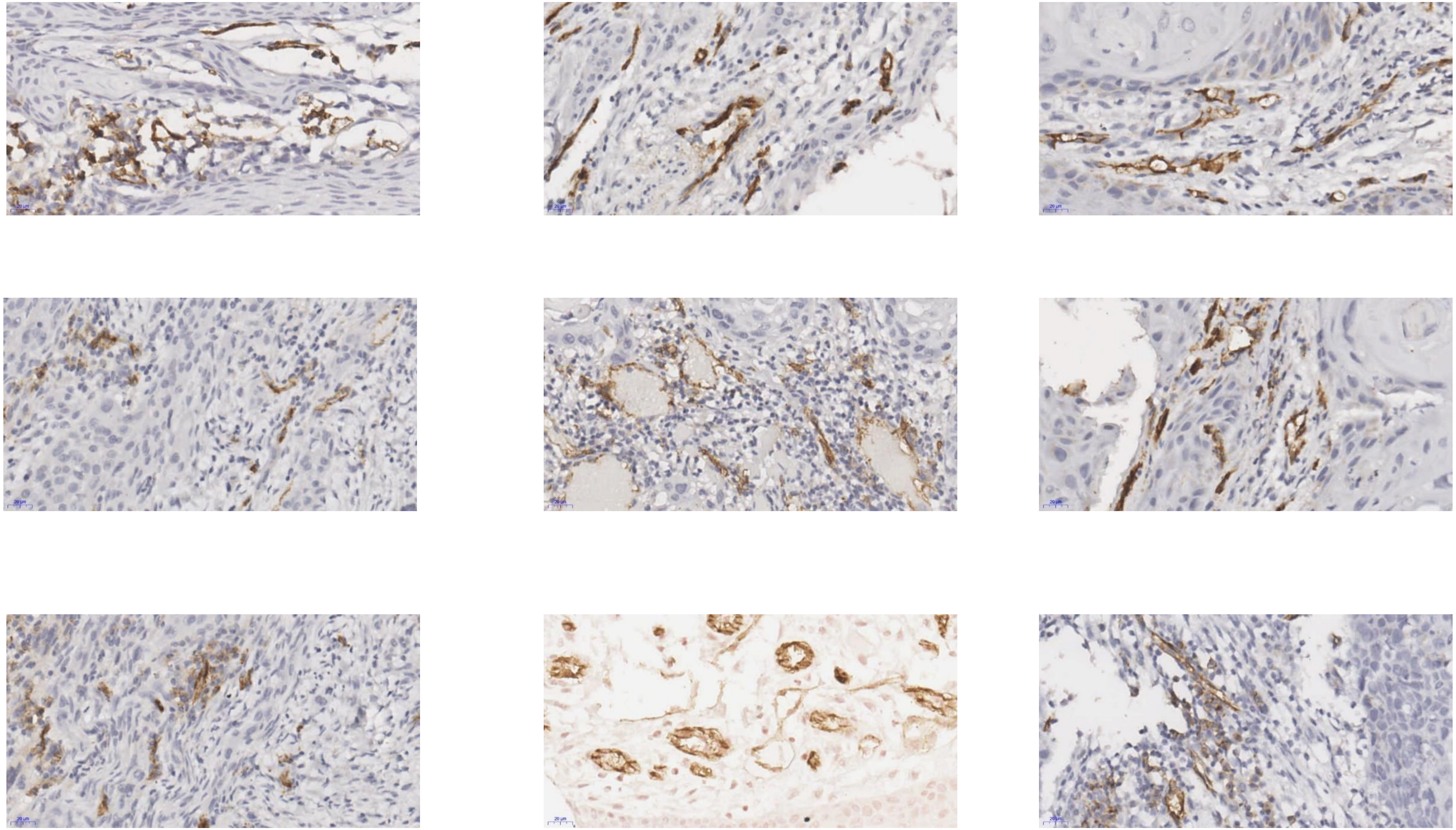

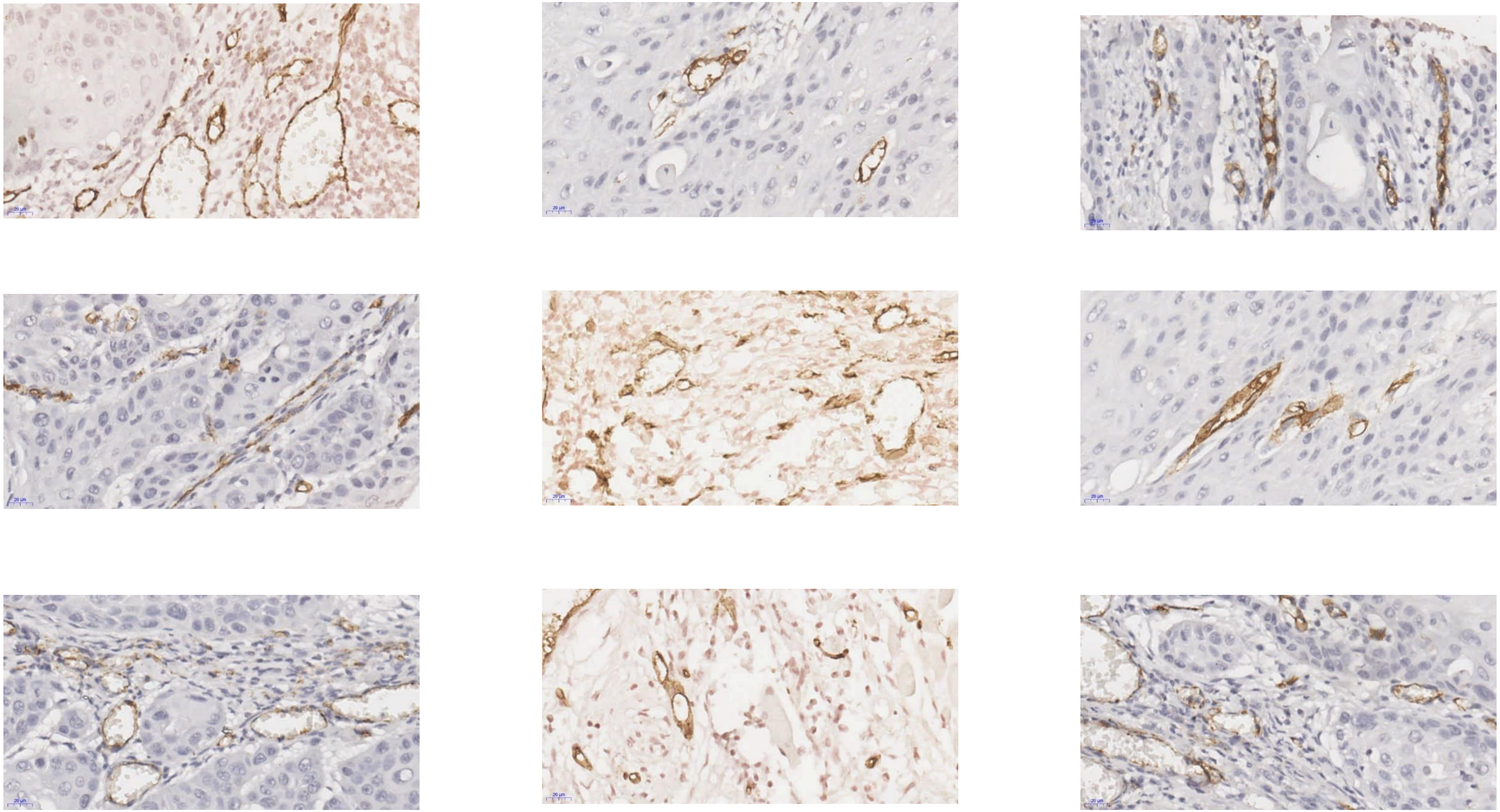

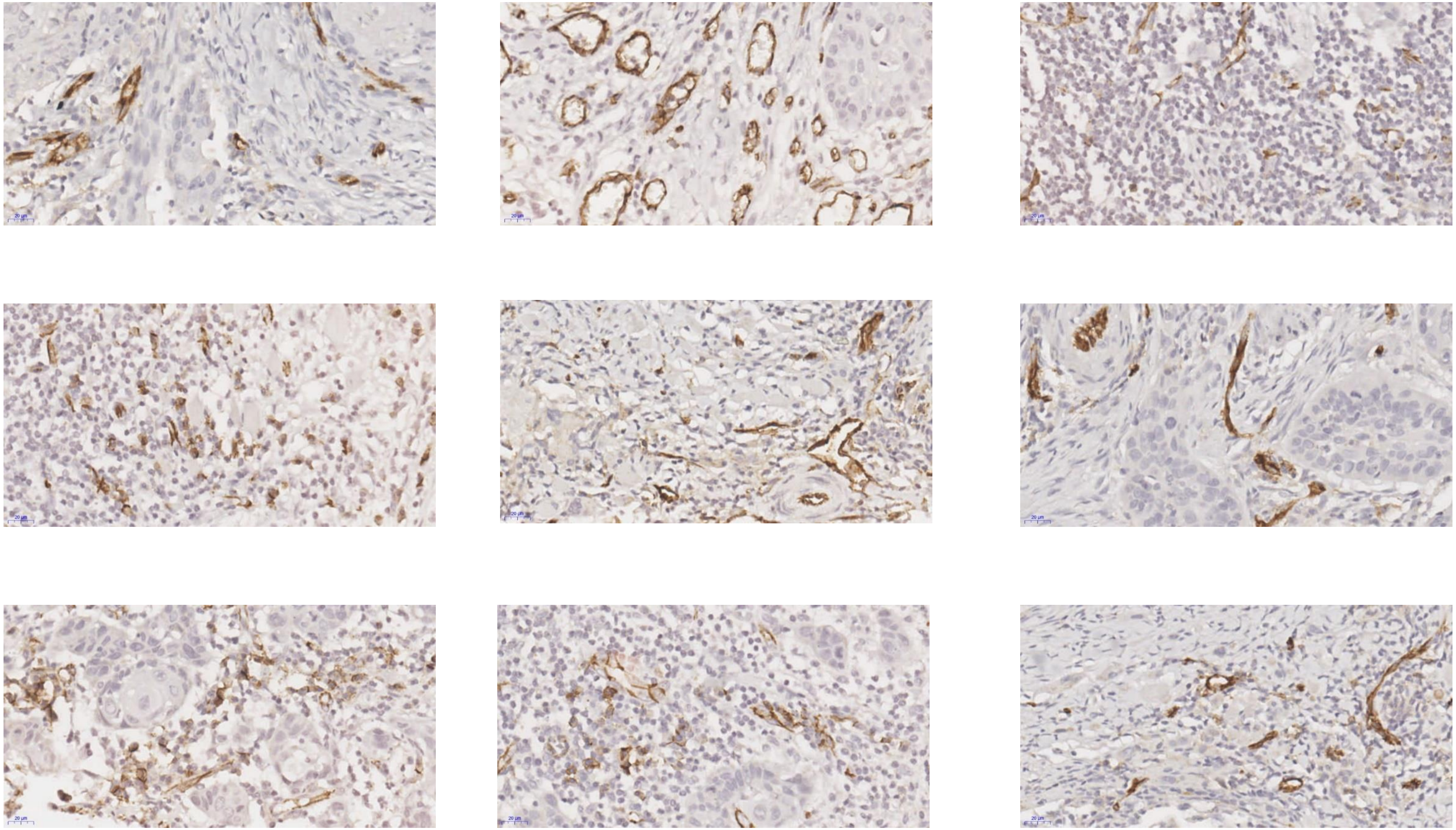

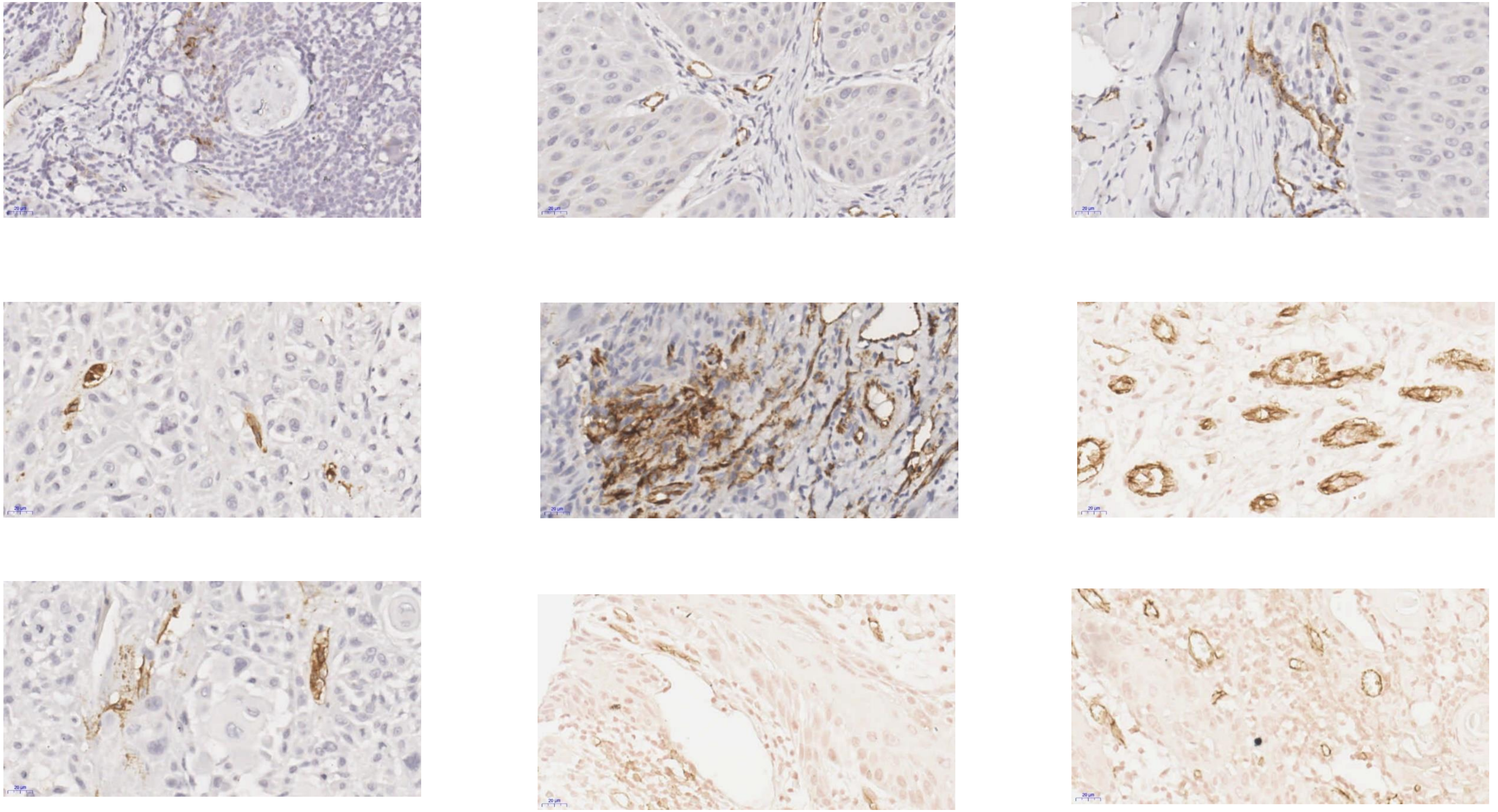

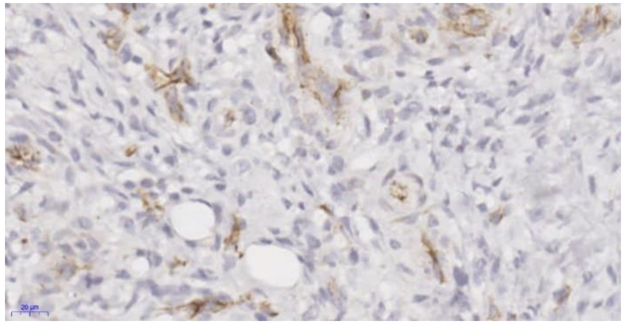
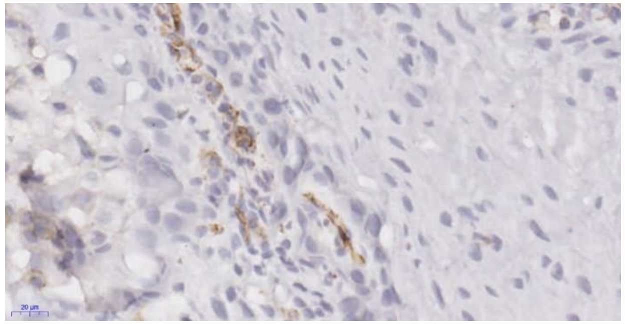
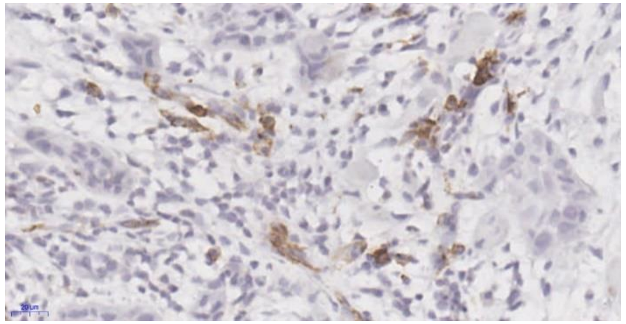
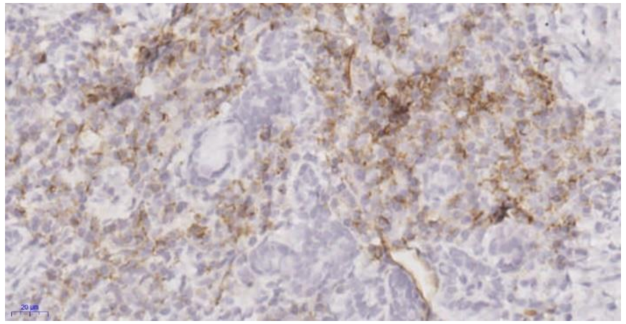
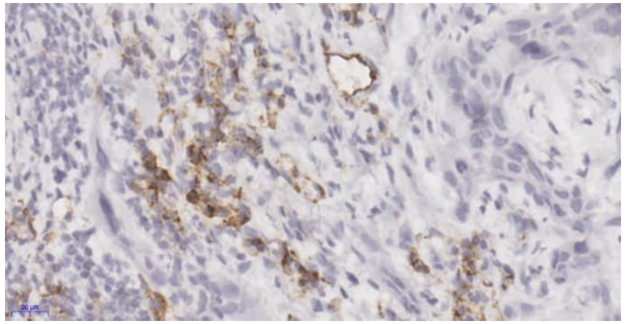
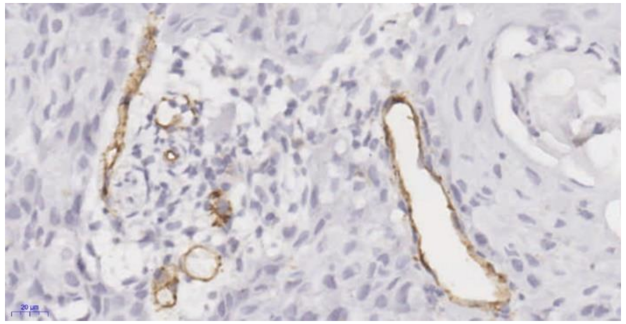
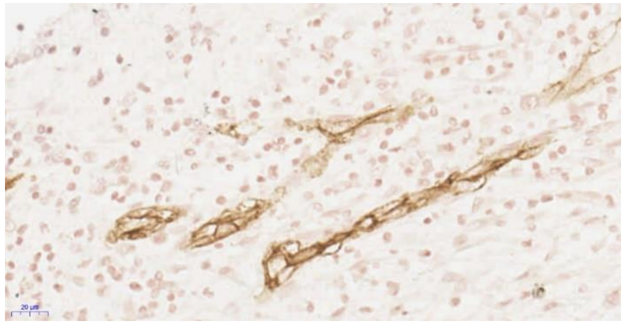
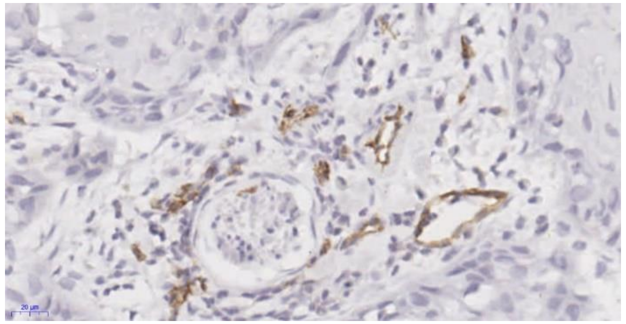
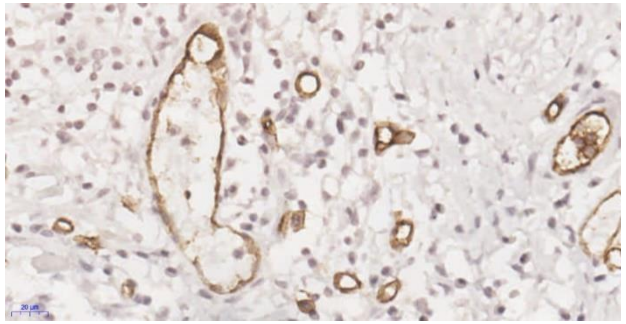

Supplement: Supplemental Information 6 [file peerj-12-17222-s006.pdf]
